# Epigenetic Effects Promoted by Neonicotinoid Thiacloprid Exposure

**DOI:** 10.3389/fcell.2021.691060

**Published:** 2021-07-06

**Authors:** Colin Hartman, Louis Legoff, Martina Capriati, Gwendoline Lecuyer, Pierre-Yves Kernanec, Sergei Tevosian, Shereen Cynthia D’Cruz, Fatima Smagulova

**Affiliations:** ^1^EHESP, Inserm, Institut de Recherche en Santé, Environnement et Travail – UMR_S 1085, Université de Rennes 1, Rennes, France; ^2^Department of Physiological Sciences, University of Florida, Gainesville, FL, United States

**Keywords:** neonicotinoids, thiacloprid, epigenetics, meiosis, testis

## Abstract

**Background:**

Neonicotinoids, a widely used class of insecticide, have attracted much attention because of their widespread use that has resulted in the decline of the bee population. Accumulating evidence suggests potential animal and human exposure to neonicotinoids, which is a cause of public concern.

**Objectives:**

In this study, we examined the effects of a neonicotinoid, *thiacloprid (thia)*, on the male reproductive system.

**Methods:**

The pregnant outbred Swiss female mice were exposed to *thia* at embryonic days E6.5 to E15.5 using “0,” “0.06,” “0.6,” and “6” mg/kg/day doses. Adult male progeny was analyzed for morphological and cytological defects in the testes using hematoxylin and eosin (H&E) staining. We also used immunofluorescence, Western blotting, RT-qPCR and RNA-seq techniques for the analyses of the effects of *thia* on testis.

**Results:**

We found that exposure to *thia* causes a decrease in spermatozoa at doses “0.6” and “6” and leads to telomere defects at all tested doses. At doses “0.6” and “6,” *thia* exposure leads to an increase in meiotic pachytene cells and a decrease in lumen size, these changes were accompanied by increased testis-to-body weight ratios at high dose. By using RNA-seq approach we found that genes encoding translation, ATP production, ATP-dependent proteins and chromatin-modifying enzymes were deregulated in testes. In addition, we found that exposure to *thia* results in a decrease in H3K9me3 levels in spermatocytes. The changes in H3K9me3 were associated with a dramatic increase in activity of retroelements.

**Conclusion:**

Our study suggests that gestational exposure to *thia* affects epigenetic mechanisms controlling meiosis which could lead to deleterious effects on male spermatogenesis.

## Introduction

*Neonicotinoids* (*neonics*) are the most common pesticides designed to control pest propagation in vegetables and fruits. They are now registered globally in more than 120 countries and are extensively used for seed, soil, and plant treatment (Elbert et al., 2008). The intensive use of *neonics* has led to a dramatic decline of non-target organisms such as bees (Rundlof et al., 2015), especially bumblebee (Whitehorn et al., 2012) colonies, worldwide. Presence of *thia*, a neonicotinoid, had been identified in 75% of the honey samples from Europe (Mitchell et al., 2017) as well as in the feathers of house sparrows (Humann-Guilleminot et al., 2019) indicating their widespread prevalence in the environment. Although *neonics* were originally thought to be safe, recent studies show that they cause developmental alterations in chicken embryos (*Gallus gallus domesticus*) (Salvaggio et al., 2018), affect common carp (*Cyprinus carpio*) growth rates (Velisek and Stara, 2018), and illicit abnormal foraging behavior in carpenter ants (*Camponotus japonicus*) (Jung et al., 2018). Potential health concerns for humans and other mammals have also been indicated (Han et al., 2018). Though neonics were designed to act exclusively on nicotinic acetylcholine receptors in insects, they can bind to a specific subtype of cholinergic receptors (Tomizawa and Casida, 2003) in the nervous system of mammals. The deregulation of these receptors has been linked to Alzheimer disease (Ferreira-Vieira et al., 2016), Parkinson’s disease (Jurado-Coronel et al., 2016), and depression (Hurst et al., 2013).

According to the US Environmental Protection Agency (US EPA), *thia* is likely a carcinogen based on a study that reported thyroid tumors in male rats and uterine and ovarian tumors in mice after a 30-day exposure to a high dose of 112.5 mg/kg/day of *thia* (U.S. Environmental Protection Agency; Sekeroglu et al., 2014). In our study, we hypothesized that exposure to *thia* during gestation, where cellular proliferation is very high and when somatic and germline cell lineages are formed, could be a susceptible period. During early gestation, a short reprogramming window known as somatic-to-germline reprograming takes place, wherein embryonic germ cells reset most of their epigenetic marks at many regions of the genome. This period is known to be particularly vulnerable to several environmental assaults (Hao et al., 2016; Gely-Pernot et al., 2018).

During puberty, the male germ cells enter meiosis, a special type of cell division that produce genetically distinct haploid cells through homologous recombination. During meiosis, programmed double-stranded breaks (DSBs) are formed throughout the genome to initiate homologous pairing of chromosomes, following which they get repaired. The introduction and repair of DSBs is a very dynamic process; the precise repair of DSBs is essential for genome integrity and perturbations in this process could lead to aneuploidy and loss of genetic information. The meiotic DNA repair requires the coordinated action of numerous DNA repair and epigenetic factors, including distinct (Kota and Feil, 2010) histone posttranslational modifications. For example, H3K4me3 and H3K36me3 methylation marks introduced by PRDM9 are essential for proper DSB formation (Powers et al., 2016), whereas di- and tri- methylation of H3K9 are required for proper organization of transcriptionally repressive heterochromatin region (Takada et al., 2011). The assembly of heterochromatin at centromeres is essential for the accurate segregation of chromosomes during cell division, and the formation of such specialized structures at telomeres protects chromosomes from degradation and from aberrant chromosomal fusions (Grewal and Elgin, 2002). Moreover, repetitive DNA sequences such as transposable elements are often assembled into heterochromatin that, in addition to its role in transcriptional repression, maintains genome integrity by suppressing recombination between repetitive elements (reviewed in Hauer and Gasser, 2017).

Thus, formation of heterochromatin during embryonic development is critical for the control of retroelements, and H3K9me3 marks introduced by SUV39h1/h2 and/or SETDB1 appear to be a major epigenetic mechanism regulating retroelement silencing in embryonic cells (Lehnertz et al., 2003; Karimi et al., 2011).

Our previous studies showed that gestational exposure to environmental toxicants affects germ cell reprogramming and leads to alterations in many organs, including brain, liver, testis and prostate. Given the fact that several neonicotinoids could be identified in the urine of pregnant women (López-García et al., 2017) and children (Ikenaka et al., 2019), we hypothesized that the delicate gestational epigenetic reprogramming window could be susceptible to the effects of *thia*. To test our hypothesis, we administered *thia* to pregnant mice and assessed its effects on the reproductive processes of the immediate generation at their adult age with the focus on epigenetic mechanisms essential for heterochromatin establishment.

## Materials and Methods

### Ethics Statement

The animal facility used for the present study is licensed by the French Ministry of Agriculture (agreement D35–238–19). All animal procedures were performed according to the Ethics Committee of the Ministry of the Research of France (agreement number: 17473-2018110914399411). All experimental procedures followed the ethical principles outlined in the Ministry of Research Guide for Care and Use of the Laboratory Animals and were approved by the local Animal Experimentation Ethics Committee (C2EA-07).

### Mice Treatment and Dissection

Pregnant, outbred, Swiss (RjOrl), female mice were treated with *thia* (0.06, 0.6, and 6 mg/kg/day) by administering the compound down the esophagus and into the stomach using a gavage needle from embryonic day E6.5 until E15.5, which corresponds to the somatic-to-germline transition window. For each dose, a minimum of four unrelated pregnant female mice were treated, and for each assay, a minimum of four males from different litters were used. *thia* was suspended in olive oil; the control mice received only oil. F1 generation male mice derived from treated and control groups at the age of 35 days or 4 months were euthanized via cervical dislocation. Both testes and epididymides were dissected and immediately frozen using liquid nitrogen to avoid protein and RNA degradation and stored at –80°C for the further analysis. One testis from each animal was fixed in paraformaldehyde and embedded in paraffin blocks. The schematic details of the experiments are presented in Supplementary Figure 1.

### Morphology Analysis and Immunofluorescence on Paraffin Sections

For morphology analysis and immunofluorescence experiments, the testes from control and *thia*-treated groups were fixed in 4% (w/v) PFA solution for 16 h, dehydrated and embedded in paraffin. The sections were cut with a microtome with the thickness of 5 μm. The sections were deparaffinized, rehydrated, and stained with Hematoxylin and Eosin (H&E) as per the standard protocol. The images were taken with NanoZoomer and quantitative analysis was performed using ImageJ. Images with tubules showing the presence of all cell types including the presence of elongated spermatids (stage VI or VII) were used for cell counts using ImageJ application. For the seminiferous tubule stage classification, we used the binary decision key for staging in mouse provided in Meistrich and Hess (2013). Cells were enumerated and divided by the total area of the tubule. We counted cells in a minimum of 10 tubules using four biological replicates. For the analyses of each testis section, stages with all cell types (spermatocytes, round spermatids, and some elongated spermatocytes) tubules were selected and the total area of each tubule was measured in square micrometers using Image J. Cells were counted in a quarter of the section of each tubule, and the result was expressed as the number of cells per 1000 μm^2^, obtained by applying the formula: *n* = (number of cells counted^∗^4/total area of the tubule) ^∗^1000. The data were averaged and plotted as compared to the control group (±SD). Statistical significance was assessed with Kruskal–Wallis test.

For the lumen size, we measured the diameter in the center of all tubules. We used round and unbroken tubules for analysis. We measured diameters in all tubules found in each section disregarding their stage. The data was averaged for each replicate and presented as lumen size compared to control. Statistical significance was assessed with a non-parametric test.

For immunofluorescence, the epitopes were unmasked in 0.01 M citrate buffer, pH 6 at 80°C for 20 min. After blocking in BSA containing 1X PBS-0.05% Tween (PBS-T), the sections were stained with antibodies against mouse anti-γH2AX (1:500, Millipore 05-636) and rabbit anti DDX4 (1:200, Abcam ab13840) antibodies. The sections with primary antibodies were incubated overnight at 4°C in a humidified chamber. After washing in PBS-Kodak (0.04%), the sections were incubated with appropriate fluorescent secondary antibodies (1:250) for 1 h in a humidified chamber at room temperature. The sections were mounted using Vectashield solution containing 0.001% (v/v) 4,6-diamidino-2-phenylindole dihydrochloride (DAPI). The images were taken using an AxioImager microscope equipped with an AxioCam MRc5 camera and AxioVision software version 4.8.2 (Zeiss, Le Pecq, France) with a 20X or 40X objective lens. We analyzed cells in *pachytene-diplotene* stage where γH2AX localized mainly in sex chromosomes, in *leptotene-zygotene* (strong staining all over the nucleus) and in round spermatids without gH2AX staining (round cells). The schematic presentation of γH2AX in cell type shown in Supplementary Figure 2. We analyzed a minimum of 10 tubules from four control and four *thia* “6” exposed animals. Cells at the corresponding stage were enumerated and presented as average number of cells per tubule ± SD. Statistical significance was assessed with a non-parametric test.

For quantitative analysis of H3K9me3 in section, we immunostained slides with rabbit anti-H3K9me3 (1:500, Abcam, ab8898) and co-stained the slide with mouse anti-γH2AX (1:500, Millipore 05-636). We analyzed immunofluorescence in zygotene and pachytene stages which were distinguished according to the pattern of γH2AX staining (schematic representation of H3K9me3 is shown in Supplementary Figure 2). *Leptotene-zygotene* and *pachytene-diplotene* stages exhibit high H3K9me3 presence, whereas in spermatids, the signal is low. Immunofluorescence was analyzed in a minimum of 20 cells in four sections for each cell type. The contour of DAPI staining was used for analysis of the nucleus area and for subtracting the background signal from each image. The data was averaged per replicate and presented as averaged corrected fluorescence for control and exposed group for each cell type. Statistical significance was assessed with a non-parametric test.

For quantitative analysis of Sertoli cells in sections, we immunostained slides with mouse anti-GATA1 (1:500, Santa Cruz, Santa Cruz sc-265). The GATA1 positive cells were enumerated per tubule and the number was divided by the total area of the tubule. The data were presented as average cell number per tubules. Statistical significance was assessed with a non-parametric test.

### Spermatozoa Extraction and Quantification

Epididymides collected from 35 day-old and 4-month-old mice were used for spermatozoa quantification. Briefly, epididymides were suspended in 1 mL of 0.05% Triton buffer, incised using small scissors, and homogenized for 45 s using a homogenizer (POLYTRON^®^ PT 2500 E). The homogenate was transferred to a 15 mL conical tube and stored on ice. One-mL aliquots of 0.05% Triton-X100 buffer were added individually to the original homogenizing vessel, homogenized, and added to the original homogenate volume. This process was repeated five times for each sample to completely obtain all spermatozoa in a final volume of 6 mL. Spermatozoa from each sample were then enumerated using a hemocytometer. Data was obtained by taking the average of ten squares from each biological replicate and plotted in Excel, presented as average number of spermatozoa per epididymis. Approximately ten biological replicates were used for each group, a Kruskal–Wallis test and pairwise Mann–Whitney comparisons were applied to establish statistical significance.

### Histone Extraction

Histone extraction was conducted to analyze epigenetic mark occupancy using Western blotting techniques. Extraction was performed using a histone extraction kit (Abcam, ab113476) according to the protocol provided by the manufacturer. Testes stored at –80°C were used for histone extraction. Briefly, testes were homogenized with a Dounce homogenizer and cell extracts were pelleted via centrifugation. The pelleted cells were then suspended in a pre-lysis buffer and incubated on ice for 10 min. Cells were pelleted again with centrifugation, resuspended in three volumes of lysis buffer, and incubated on ice for 30 min. The supernatant was collected and 0.3 volumes of DTT (ab113476) were added. Histone protein concentrations were quantified using an optical density (OD) reading at 600 nm using Pierce’s solution.

### Western Blotting

Western blots were prepared for the control group and *thia* dose of 6 mg/kg/day using the following antibodies: anti-histone H3K9me3 (Abcam, ab8898, 1:1000), H3K4me3 (Milipore 07-473, 1:1000) or unmodified histone H3 (Abcam, ab1791, 1:2000). Histone proteins purified through histone extraction were used for Western blotting. Concentrations of each histone protein sample, obtained from OD readings, were used to determine an exact volume containing 10 μg of histone protein for each sample. Aliquots of 20 μL containing 25 mM Tris buffer, Laemmli 4X buffer, and sample volumes containing 10 μg of histone proteins were denatured at 96°C for 10 min and run on a 4-15% gradient acrylamide gel (Mini-PROTEAN^®^ TGX^TM^ Precast Protein Gels). Proteins were transferred onto Polyvinylidene difluoride (PVDF) membranes (Millipore, France) using an electro-blotter system (TE77X; Hoefer, United States) and modified Towbin buffer [48 mM Tris base, 40 mM glycine and 0.1% (wt/vol) SDS] and methanol [20% (vol/vol) anode; 5% (vol/vol) cathode] for 1.15 h. Ponceau Red staining was conducted to determine the relative abundance of total histone proteins. Imaging was conducted using a molecular imager (ChemiDoc^TM^ XRS + System with Image Lab^TM^ Software). Blocking was conducted using a 5% milk in 1X TBS Tween 0.05%. The primary antibody was diluted 1:1000 in 10 mL of the blocking solution and introduced to each membrane in small plastic bags, which were incubated overnight at 4°C. After three, 10-min washes with 1X TBS, each membrane was incubated for 1 h in 40 mL of the blocking solution containing the corresponding secondary antibodies (GE Healthcare, United States). After another three 10-min washes with 1X TBS, Western blotting detection reagents (Amersham ECL^TM^ Prime Western Blotting Detection Reagents) were used to coat each membrane for the development of the first and secondary antibody complex. Specific protein expression for each antibody was then photographed using a molecular imager. Histone H3 immunoblot pictures showing the intensity of total histone abundances were used to normalize the occupancy of each specific epigenetic marker to the total amount of histone proteins for each sample. Intensity of the bands were measured in Fiji: ImageJ software (Schindelin et al., 2012). Western blotting signals were presented as averaged normalized values.

### RNA Extraction and RT-qPCR

Testes stored at –80°C from 35-day-old mice from the control group and from the 6 mg/kg/day treatment group were used for RNA extraction. Extraction was performed using RNeasy Plus Mini Kit (QIAGEN^®^). Approximately 30 mg of each testis was used. Testes samples were lysed and subsequently homogenized using a 26-gauge syringe. DNA was removed by passing the solution through a DNA elimination column. One volume of 70% ethanol was added to the lysate to provide ideal binding conditions. The lysate was then loaded onto the RNeasy silica membrane. RNA bound to the silica membrane was then washed with RW1 and RPE solutions to remove impurities. Purified RNA was then eluted in 50 μL of RNase-free water. Six biological replicates of control and treated groups were used for RT-qPCR. RT was performed using 1 μg of total RNA with either iScript (BioRad, 1708891) or QuantiTect Reverse Transcription Kit (Qiagen, 204443) adhering to the Minimum Information for Publication of Quantitative Real-Time PCR Experiments (MIQE) guidelines (Bustin et al., 2009). The kit includes a DNA elimination step prior to the RT reaction. A no-RT reaction was performed and analyzed by qPCR for all the tested genes. The reactions showed no presence of PCR products. We used *Rpl37a* as housekeeping gene as it showed no variation between replicates, based on RNA-seq data. The data were presented as fold change compared to control ± SD. Primers for this study were selected using Primer-Blast program from ncbi.nih.gov and most of them include exon-to-exon junctions. Primers used in this study are listed in Supplementary Table 1. A non-parametric Mann–Whitney test was used for statistical significance.

### RNA Sequencing and Data Processing

A strand-specific library preparation protocol (NEBNext Ultra II Directional RNA Library) for RNA sequencing was performed in the lab according to the protocol provided by manufacture by using 1 μg of total RNA. Quality control and the genome-wide sequencing was performed at GenomEast platform at the Institute of Genetic, Molecular and Cellular Biology (IGBMC), Strasbourg, France. We used three biological replicates for these experiments. The sequencing was performed in massive parallel sequencing paired-end mode, and the size of the sequencing tag was 100 bp.

Reads in FASTQ format were processed for quality control using the FastQC tool^1^. An average of 217 million sequencing reads per sample was processed. The exact number of reads per sample is provided in Supplementary Table 2. The reads were mapped to the reference genome [*Mus musculus* Ensembl mm10 sequence] using Hisat2 alignment program with default parameters, and the alignment files were generated as BAM files. These files were used as the input for StringTie (v1.3.5), a complementary method used to generate assembled transcripts for each condition, and to calculate the transcript abundance. Differential gene expression was assessed using the R package DESeq2 (v1.25.0). To identify transcripts that were differentially expressed between thiacloprid and control-derived samples, we first selected the cases for which we obtained values greater than the 50th centile of all values in at least one condition, and we then filtered the transcripts for which there was a more than a 1.5-fold difference between the *thia*- and control-derived samples. The false-discovery rate (FDR) threshold was set at 5%. Genes with higher FDR were not considered as differentially expressed. RNA-seq data were visualized using the Integrated Genomics Viewer (version 2.4.2) (Thorvaldsdottir et al., 2013). Functional annotation of differentially expressed genes (DEGs) was performed with DAVID program (Huang da et al., 2009) and GSEA (Subramanian et al., 2005) using default parameters.

RNA sequencing was performed in whole testis. To get insight into potential cell types contributing to gene expression, we used recently published datasets from testes cell types using single cell sequencing (Green et al., 2018). For each cell type, we calculated the Fold Change (FC) of gene expression in the considered cell type compared to the average of all the other cell types to estimate expression level in each cell type. Then, to determine whether the gene expressions are specific to a cell type compared to the others, we compared the average value of all cell types and the average value for a given cell type. We considered the gene to be mostly expressed in the given cell type if the ratio is above 2. Thus, we were able to associate each DEG with possible cell type origin.

### Meiotic Surface Spreads Preparation for Double-Strand Breaks and Defective Chromosomal Synapsing Assays

Meiotic surface spreads were prepared from testes of 35-day-old mice according to the previously described protocol (Peters et al., 1997). Samples were placed in PBS, pH 7.4, at room temperature. The *tunica albuginea* was removed, and extra tubular tissue was removed by washing the seminiferous tubules with PBS. Some tubules were placed in a hypotonic extraction buffer containing 30 mM Tris, 50 mM sucrose, 17 mM trisodium citrate dihydrate, 5 mM EDTA, 0.5 mM DTT and 0.5 mM phenylmethylsulfonyl fluoride (PMSF), pH 8.2, for 30–60 min. A suspension was created in 40 mL of 100 mM sucrose, pH 8.2, on a glass slide. Tubules approximately 2–3 cm in length were broken into pieces with forceps in 20 mL of sucrose solution. The volume was increased to 40 mL and a 10 mL pipette was used to mix the solution until cloudy. The tubular remains were removed, and each suspension was divided into two clean glass slides that had just been dipped in 1% paraformaldehyde solution containing 0.15% Triton X-100. The cell suspension was then placed in a corner of the slide and dispersed across the entire slide, while exposing the cells to the fixative. Nuclei were dried overnight in a humidity chamber at room temperature. Slides were then washed four times for 1 min each in 0.4% Kodak Photo-Flo in PBS and dried at room temperature. The slides were then stored at –80°C until use.

Meiotic surface spread slides were incubated in a humidity chamber for 20 min at 37°C with a 1X antibody-dissolving buffer (ADB) buffer containing 1% donkey serum, 0.3% bovine serum albumin (BSA), and 0.005% Triton X-100 in PBS. Primary antibodies, anti-SYCP3 (Santa Cruz, sc-74569) and anti-DMC1 (Santa Cruz, sc-22768), were then diluted 1:100 in 1X ADB solution. Slides were then incubated at 4°C overnight with 50 μL of primary antibody solution with a coverslip. Two, 5-min washes were then performed with 0.4% Kodak Photo-Flo in PBS. Secondary antibodies, Alexa594 goat anti-mouse and Alexa488 chicken anti-rabbit were diluted 1:250 in 1X ADB solution. Slides were then incubated at room temperature for 20 min with 50 μL of secondary antibody solution with a coverslip. Two, 5-min washes were then performed with 0.4% Kodak Photo-Flo in PBS followed by two washes with 0.4% Kodak Photo-Flo in water. Slides were air-dried and then 35 μL of Vectashield^®^ Mounting Medium with DAPI (Vector Laboratories) was added to each slide with a coverslip in order to stain nuclear DNA. Slides were then stored at –20°C until use.

Double-strand breaks (DSBs) were visualized with fluorescence of DMC1, which is a recombinase protein involved in repairing DSBs in meiotic homologous recombination that binds to single-stranded DNA (ssDNA) (Li et al., 1997). SYCP3 (synaptonemal complex protein 3 fluorescence) was used to visualize chromosome structure since this complex is involved in synapsis, recombination, and segregation of meiotic chromosomes (Kobayashi et al., 2017). Early *pachytene*, have a higher number of breaks, and we used the manuscript (Page et al., 2012) for discrimination of *pachytene* from *diplotene* based on synapsing pattern of sex chromosomes. The synapsed sex chromosomes in early *pachytene* stage start to de-synapse in mid *pachytene* stage. DMC1 foci were counted in all autosomes and sex chromosomes separately in a minimum of 15 cells and the average number of foci per four biological replicate per group was compared for all tested groups. The statistical significance was assessed using a meta-analysis relying on z-tests implemented in the metafor R package (v2.4).

Anti-rabbit SYCP1 (1:200, Abcam, ab15090) and anti-mouse SYCP3 (1:200, Santa Cruz, sc-74569) were also used for the examination of synaptonemal complexes to observe synapsing defects of chromosomes. A minimum of 75 images of cell in *pachytene* stage were taken using fluorescent microscope from a minimum of four biological replicates from exposed group “6” and control group. Synapsing defects that were enumerated in control and treatment groups showed incomplete synapsing and multiple connections (more than one chromosome joined together). Cells were examined and scored accordingly. Normal chromosomes were those that were fully synapsed and SYCP1 and SYCP3 were fully colocalized except sex chromosomes, where only partial colocalization was detected. Cells containing more than one type of defect were scored in each defect category present. The statistical significance was assessed using a meta-analysis relying on *z*-tests implemented in the metafor R package (v2.4). This method allows comparing each dose against the control group taking into account the intra-group variability.

Telomere defects were analyzed as follows. The slides with DMC1 and SYCP3 immunostaining were used for this analysis. The defect was considered as an end-to-end defect if two different chromosomes were close, and as ring chromosome when both ends of the sex chromosomes form the ring. Telomere defects were enumerated in 75 pachytene cells for four biological replicate per group and presented as averaged value in percent to a total analyzed cell, the statistical significance was assessed using a meta-analysis relying on *z*-tests implemented in the metafor R package (v2.4).

Telomere protein analysis was completed using mouse TERF1 antibody (Santa Cruz, SC-56807, 1:20) and rabbit SYCP3 (Santa Cruz, SC-33195, 1:100). Images were analyzed using Fiji and the intensity of fluorescence at each telomere was calculated and average TERF1 intensity was calculated for each cell. Statistical significance was assessed by a non-parametric test. Finally, we compared the average signal of TERF1 in normal and fused telomere cells from the *thia* exposed group dose “6.”

### Preparation and Immunostaining of Structurally Preserved Nuclei

Structurally preserved nuclei (SPN) for three-dimensional analysis were prepared by cutting testis tissues in DMEM medium (Life Technologies, GIBCO) with 0.5% protease inhibitor. The cell suspensions were mixed with equal amounts of 3.7% (vol/vol) paraformaldehyde and 0.1 M sucrose and spread on glass slides. The slides were air-dried and kept at –80°C. The slides were then washed several times with PBS and 2 min with 0.1M glycine in PBS to remove the traces of paraformaldehyde before use. Slides were permeabilized during a 30 min incubation in PBS/0.5%Triton at RT, washed with PBS, and blocked for 30 min in a solution containing 0.1% (v/v) donkey serum, 0.03% (w/v) BSA, and 0.005% (v/v) Triton X-100 in PBS. SPN were immunostained with rabbit H3K9me3 (Abcam, ab8898, 1:500) or mouse monoclonal SYCP3 (1:200, Santa Cruz, SC-33195) antibodies at 4°C overnight followed by several washes and incubation with Fluorescent Alexa antibodies. Z-stacks were acquired with 500 nm steps. Twenty one planes were taken for each individual channel, DAPI (Blue, 350 nm), SYCP3 (Red, 594 nm) and H3K9me3 (Green, 488 nm) using Zen Pro (version 2.3). All images for control and exposed samples were taken with fixed exposure times. Deconvolution was performed using the “Fast Iterative” algorithm provided by Zen Pro. The sum intensity images were generated for each *z*-stack and the resulting images were analyzed in ImageJ v1.52n. We used the lasso tool for nucleus contouring, and the integrated density immunofluorescence for each nucleus was calculated. The background area was subtracted from each image. We analyzed four independent biological replicates for control and treated group “6,” and at least 15 cells for each replicate at the pachytene stage were analyzed. The data were plotted in Excel and presented as corrected total cell fluorescence (CTCF) of normalized fluorescence for nucleus compared to control ± SEM, ^∗^*p* < 0.05, Mann–Whitney test.

### Statistical Analyses

We used the minimum number of animals according to the requirements of the EU ethic committee. The number of animals used was specified for each experimental procedure. We performed the non-parametric Wilcoxon–Mann–Whitney test to assess statistical significance in body weight measurements, qPCR experiments, immunofluorescence and Western blots quantifications. Telomere defects and DMC1 foci were assessed by a meta-analysis relying on *z*-tests implemented in the metafor R package (v2.4). The difference in spermatozoa number counts was assessed by the non-parametric Kruskal–Wallis test and by pair-wise comparisons using the Wilcoxon–Mann–Whitney test. *P*-values were adjusted using the Benjamini and Hochberg method. For the statistical significance of RNA-seq data, Wald’s test for generalized linear models implemented in R package DESeq2 was used.

## Results

### Gestational Exposure to *thia* Leads to Decrease in Body Weight in Male Progeny

This study aimed to reveal the effects of gestational exposure to a neonicotinoid, thiacloprid on male reproductive system with the focus on epigenetic mechanisms essential for meiosis. We chose the developmental window from embryonic day E6.5 to E15.5 due to the importance of germ cell establishment during this period. We tested four doses of *thia* “0,” 0.06,” “0.6,” and “6” mg/kg/day.

We exposed mice to four different doses of *thia* during the critical somatic to germline transition period. The F1 progeny mice were analyzed at 35 days when the first wave of spermatogenesis is accomplished. The first wave of spermatogenesis could affect the other waves, and thus, play an important role in reproductive functions (Busada et al., 2016).

**FIGURE 1:**
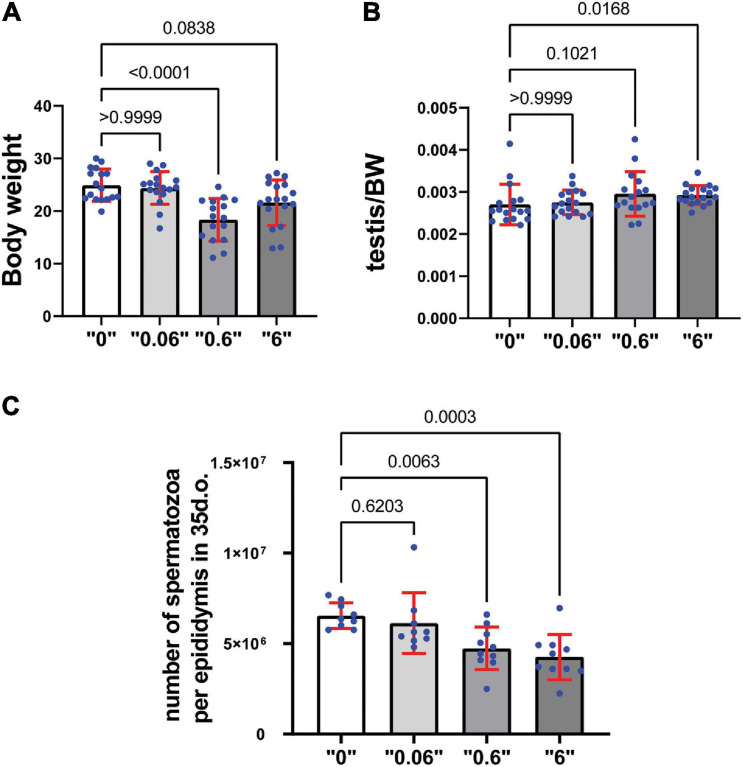
Gestational exposure to *thia* leads to alteration in reproductive parameters in 35-day-old mice: **(A)** Body weights (minimum *n* = 16 for each group), **(B)** Relative testis-to-body weight (minimum *n* = 16 for each group), and **(C)** Mean spermatozoa per epididymis, (*n* = 10 for each tested group). Exact *p*-values are indicated on the bars, Kruskal–Wallis test. Error bars represent standard deviation.

We observed a decrease in body weight (BW) at dose “0.6” and a tendency to decrease in dose “6,” by 26 and 14% respectively in 35-day-old mice (Figure 1A). To analyze *thia*’s effect on reproductive organs, the weights of testes from 35-day old mice were measured and expressed as a percentage compared to the total body weight of the animal. Testis to body weights ratios showed a tendency to increase in “0.6” *thia*-treated group and significantly increased in dose “6,” by 9 and 6% respectively (Figure 1B). No significant changes were observed in epididymis-to- BW ratios (Supplementary Figure 3).

Spermatozoa count is an important parameter that determines the normal functioning of the male reproductive system. To assess the effects of *thia* on spermatogenesis, a quantitative analysis of spermatozoa numbers was conducted for each *thia* treatment group and compared to the control group (Figure 1C) in 35-day-old. Statistical analyses indicated a significant decrease in sperm count in the experimental groups compared to control. A 24% reduction was observed for dose 0.6 mg/kg/day, a 32% reduction for dose 6 mg/kg/day in 35-day-old mice.

To assess the effects of *thia* in older animals, we also analyzed mice at 4 months of age as reproductive defects could be more apparent at an adult stage. The analysis revealed no changes in body weight (Supplementary Figure 4A). However, a significant increase in the testis/body weight ratio was detected in *thia*-treated groups at doses 0.06 and 6 mg/kg/day (Supplementary Figure 4B) compared to the control group, suggesting the observed alteration were persistent in older animals. However, no significant changes were observed in epididymis/body weight ratio (Supplementary Figure 4C). Spermatozoa number decrease 28, 34, and 47% at doses “0.06,” “0.6,” and “6,” respectively compared to the control group (Supplementary Figure 4D) suggesting that the toxic effects imposed during development was not rescued even at adulthood. In conclusion, gestational exposure to *thia* leads to an increase in relative testis weight and causes spermatozoa decline in exposed groups, suggesting deleterious effects on the male reproductive system.

### Morphological Analysis of Testis Revealed a Decrease in Lumen Size and an Increase in Cell Density in Exposed Groups

**FIGURE 2:**
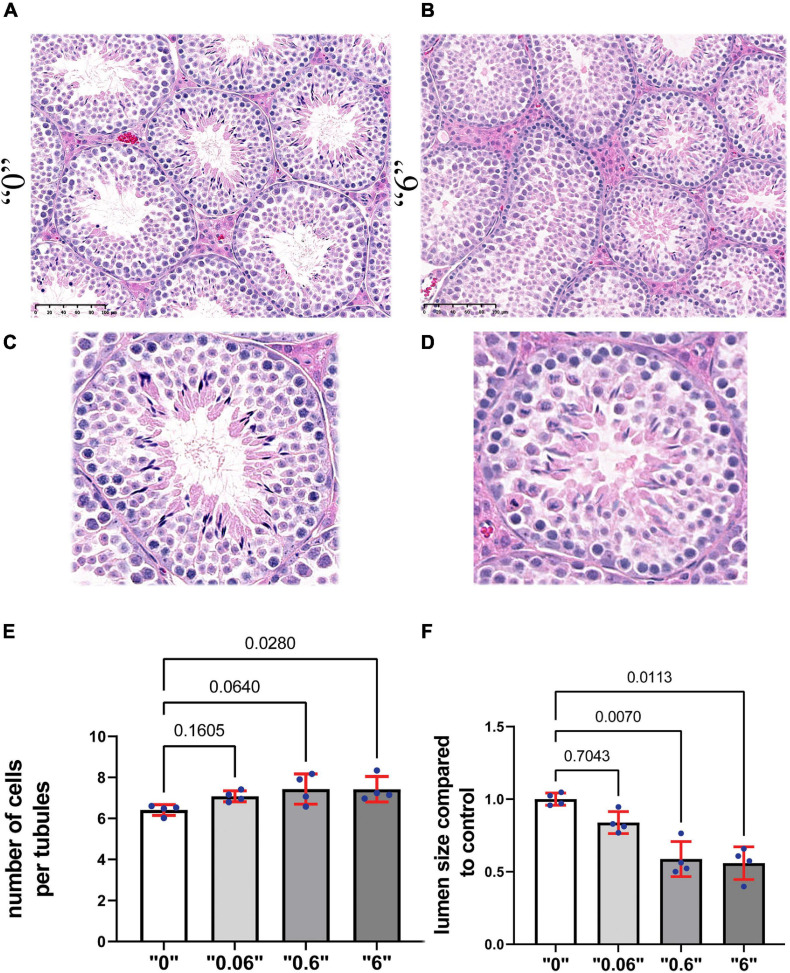
Gestational exposure to *thia* leads to alteration of testis morphology in 35-day-old mice. Testis sections from **(A)** “0” and **(B)** dose “6” (20X magnification, scale bar represents 100 μm). In “0,” several layers of cells and a well-developed lumen could be observed. **(C,D)** The magnified images are presented in the second row. **(E)** Quantitative analysis of cells in the area per tubule. Ten tubules were analyzed for each group (*n* = 4). **(F)** The size of the lumen measured “0” and thia-exposed groups. Exact *p*-values are indicated on the bars, Kruskal–Wallis test, Mann–Whitney pairwise comparisons were used for statistical test. Error bars represent standard deviation.

To reveal the effects on testis morphology, we prepared paraffin sections from 35-day old mice and stained them with hematoxylin and eosin (Figures 2A–D). We counted the number of cells in the tubules that are in stage VI or VII and calculated the total number of cells in the area of tubules per square micrometer (μm^2^). The results were averaged and presented as number of cells per 1000 μm^2^. We detected a 15% increase in cell number at dose “0.6” and “6” compared to control group suggesting that the number of cells in the tubules could be affected by the higher dose treatment (Figure 2E). We detected a significant decrease in lumen size 1.7 and 1.8 times at doses “0.6” and “6,” respectively (Figure 2F). Both circular and elongated tubules could be observed in control as well as *thia* dose “6” group (Supplementary Figure 5). Moreover, we occasionally detected the presence of vacuoles in male progeny at the highest dose, “6,” data not shown.

**FIGURE 3:**
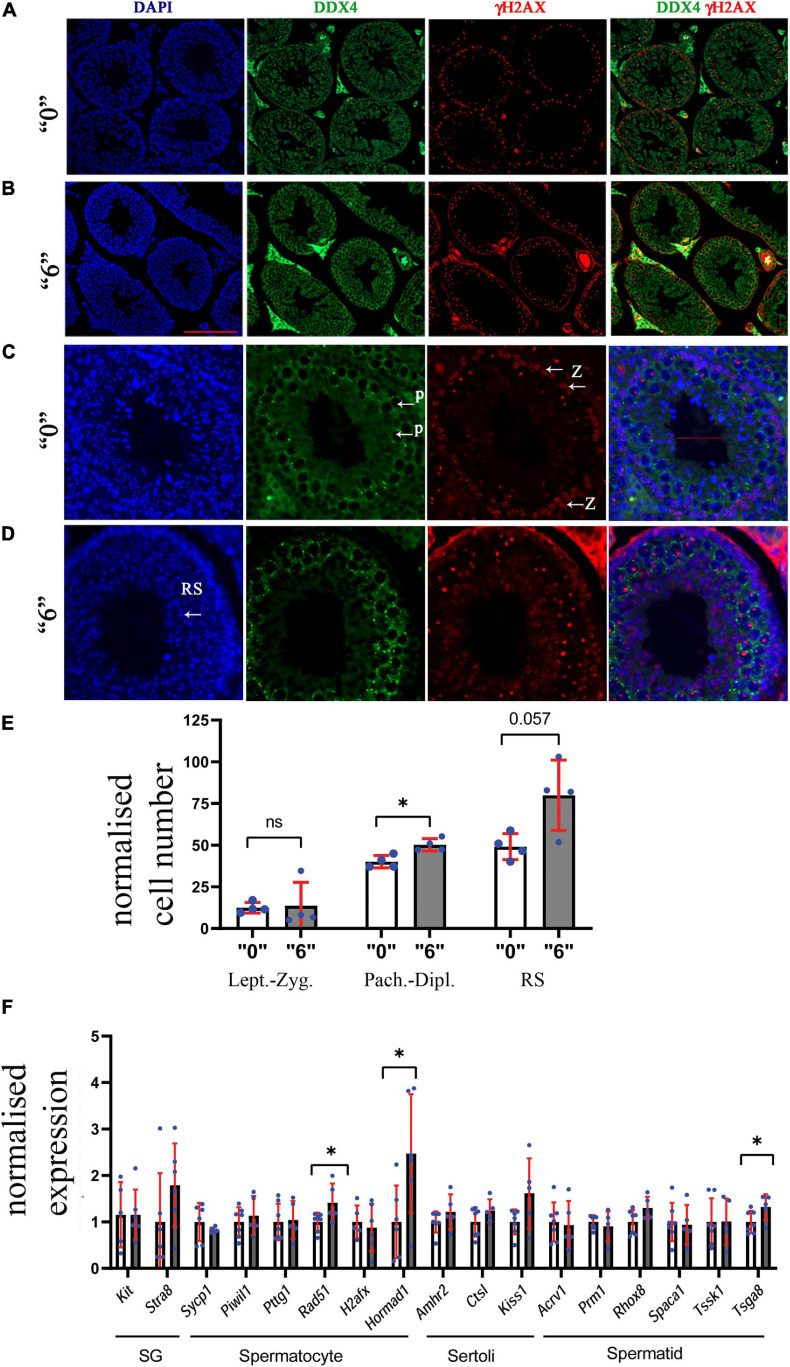
Thirty-five days old mice testis sections immunostained by γH2AX (red, a double-strand DNA break marker) and DDX4 (green, a marker of germ cells) in **(A)** “0” (20X magnification, scale bar-50 μm) **(B)** *thia* dose “6” (20X magnification) and **(C)** magnified images (40X, scale bar-50 μm) from control and **(D)** *thia* dose “6” showing cells at *leptotene-zygotene* (Z) and *pachytene-diplotene* (P) stages that were used to differentiate meiotic cell types, and round spermatids (RS) used for quantitative analyses. **(E)** Quantitative analysis showed alterations in cell type distribution (RS-Round spermatids). We counted a minimum of 10 tubules for four biological replicates per group. **(F)** Quantitative analysis of cell-specific gene expression in the testis of “0” (white bars) and *thia*-exposed at dose “6” (gray bars) in 35-day-old mice. mRNA was extracted, and analyzed by RT-qPCR using primers that are specific for spermatogonia (SG), spermatocytes (SC), Sertoli cells and spermatids (ST). Gene expression was normalized to the housekeeping gene *Rpl37a*. Six biological replicates were used for each group, (^∗^*p* < 0.05, non-parametric Mann–Whitney test, error bars represent standard deviation).

To further assess the effects of thiacloprid on germ cell number, we co-immunostained the sections with antibodies against DDX4, a marker of germ cells and γH2AX, a marker of double-strand DNA breaks. We performed analysis in control and in the male progeny derived from highest treated dose “6,” as the effects were observable at this high-dose. DDX4 is expressed in the cytoplasm, whereas γH2AX is seen as bright staining all over the nuclei in *leptotene- zygotene* and in *pachytene- diplotene* cells it is visible in sex chromosomes, the staining is normally absent in haploid cells such as round spermatids. Round spermatids were identified by DAPI staining (Figures 3A–D). The quantitative analysis showed that the number of *zygotene-leptotene* cells were not significantly affected, in contrast, the *pachytene-diplotene* cells were increased by 25%, and round spermatids by 63% per tubule (Figure 3E).

Next, to determine whether exposure to *thia* affects the somatic cell numbers, we immunostained the sections against GATA1 antibody. In Sertoli cells, GATA1 localizes in the nucleus, Supplementary Figures 6A,B. The quantitative analysis of GATA1 positive cells per tubule did not show significant alterations (Supplementary Figure 6C).

To further evaluate if there are cell type-specific changes, we performed RT-qPCR analysis using cell type-specific markers. We designed primers and performed analysis for markers specific for spermatogonia (SG), spermatocytes (SC), spermatids (ST), and Sertoli cells. As a specific marker database source, we used single cell sequencing data from Green et al. (2018). Analysis showed that SC markers such as *Rad51* and *Hormad1* have a significant increase in gene expression 1.4 and 2.4 times, respectively, suggesting possible increase in spermatocyte population. In contrast, we did not observe significant changes in spermatid-specific genes except for *Tsga8*, which had increased expression 1.2 times; no Sertoli or SG cell-specific gene expression was found to be altered (Figure 3F). We also extracted information about the gene expression that we tested in RT-qPCR from the RNA-seq data and have included it in Supplementary Figures 7A,B. *Rpl37a* amplification curve (Supplementary Figure 7C) showed reproducibility in RT-QPCR for both tested groups.

Thus, gestational exposure to thiacloprid leads to increase in testis cell density in adult male progeny.

### A Persistence in Meiotic DSBs and Chromosomes With Telomere Connection and Synapsing Defects Were Observed Following Exposure to *thia*

**FIGURE 4:**
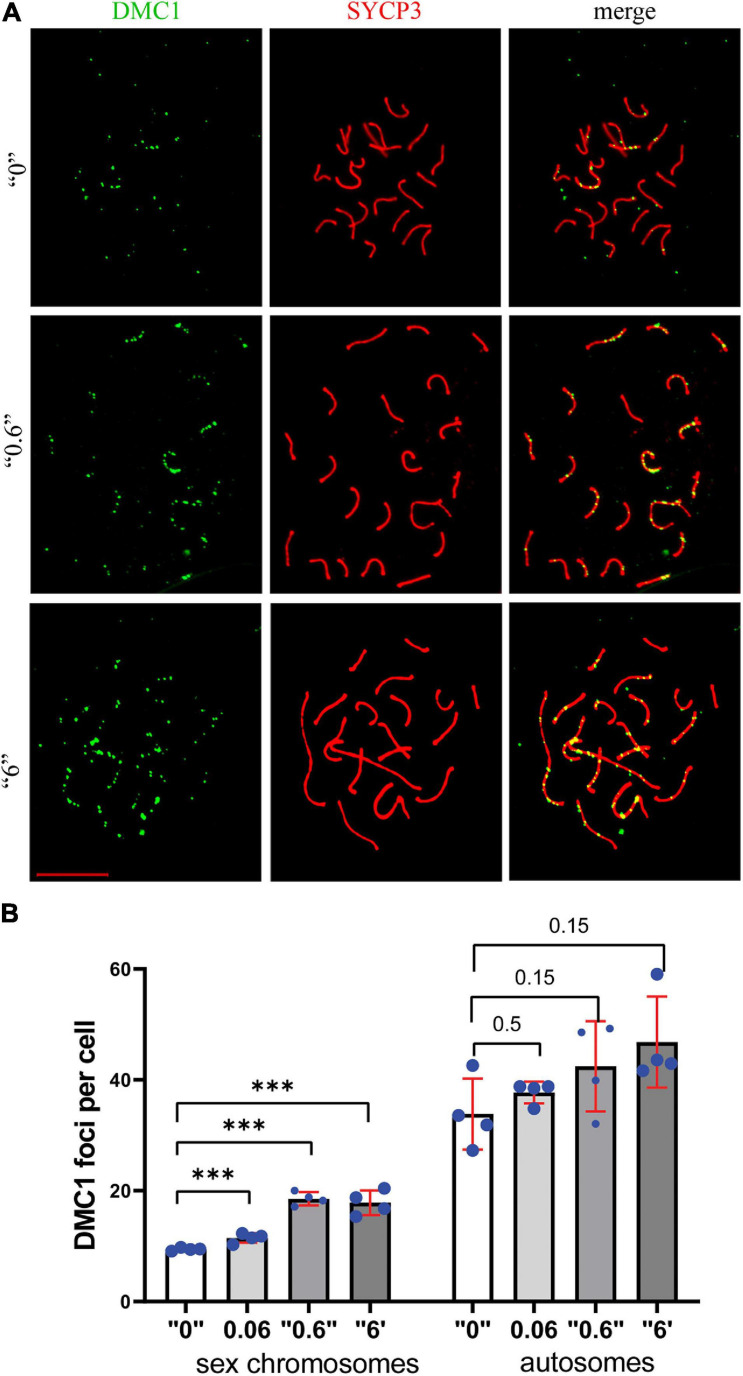
The meiotic recombination protein, DMC1 foci in *thia*-exposed male mice persists at PND 35 days. **(A)** The assembly of meiotic recombination complexes were visualized by DMC1- immunostaining foci (green), and chromosomes were visualized by immunostaining using SYCP3 (red). **(B)** DMC1 foci were counted separately in sex chromosomes and autosomes in a minimum of 15 early pachytene cells for four biological replicate per group in control group or treatment group. Error bars represent standard deviation, ^∗∗∗^*p* < 0.001, Meta-analysis relying on *z*-tests implemented in the metafor R package (v2.4).

In order to assess DNA repair efficiency between the control and treatment groups, we prepared surface spreads using 35-day-old testes and immunostained the slides against SYCP3 (red, a meiotic chromosome marker), and DMC1 (green, a marker of meiosis-specific recombinase that binds to double-strand breaks) proteins (Figure 4A). DMC1 foci were enumerated for control and exposed groups. For the analysis, we chose cells that were in early *pachytene* stage (defined as *pachytene* based on; Page et al., 2012), visually fully synapsed, and had more than ten breaks. We counted the DMC1 foci in sex and autosomal chromosomes (Figure 4B) separately to see whether there is a chromosome specific alteration. We found a higher number of DMC1 foci in sex chromosomes 1.2, 2, and 1.9 times at doses, “0.06,” “0.6,” and “6,” respectively. In autosomes, the changes were 1.1, 1.3, and 1.4 at doses, “0.06,” “0.6,” and “6,” respectively. This suggests that the increase in DMC1 foci is mainly due to a persistence of breaks in sex chromosomes.

**FIGURE 5:**
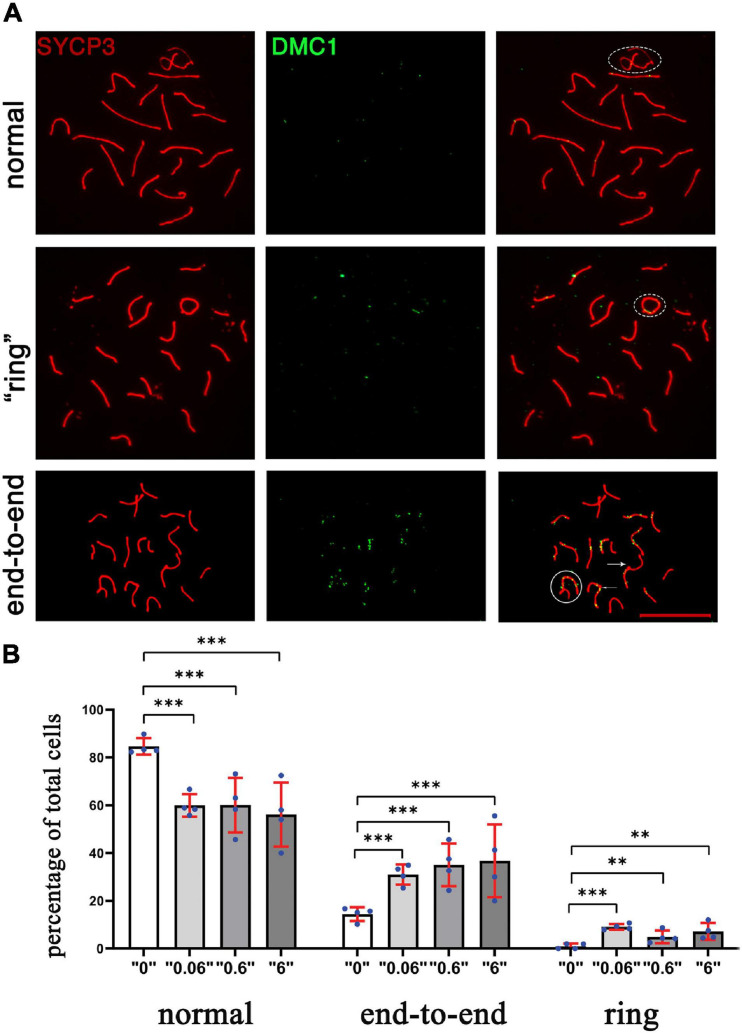
The chromosomal defects in *thia*-exposed male mice have increased. **(A)** Chromosomes were scored according to defects, including ring (second row), end-to-end (third row, marked by arrow. The sex chromosomes were outlined by oval dashed lines, the defects were marked by arrows. **(B)** Quantitative analyses showed a significant increase in end-to-end connections and ring chromosomes. We counted a minimum of 75 cells for the analyses for four biological replicate per group. [^∗∗^*p* < 0.01, ^∗∗∗^*p* < 0.001, Meta-analysis relying on *z*-tests implemented in the metafor R package (v2.4)]. Error bars represent standard deviation.

To determine whether the changes in DSB repair are associated with telomere defects, the synaptonemal complexes were analyzed and scored using a minimum of 75 cells for each replicate (Figure 5A). We identified telomere defects such as formation of “ring sex chromosomes,” and telomere end-to-end connections. All defects were tabulated and compared (Figure 5B). The statistical meta-analysis determined that there was a significant increase in the number of synapsing defects in all treatment groups versus the control group.

**FIGURE 6:**
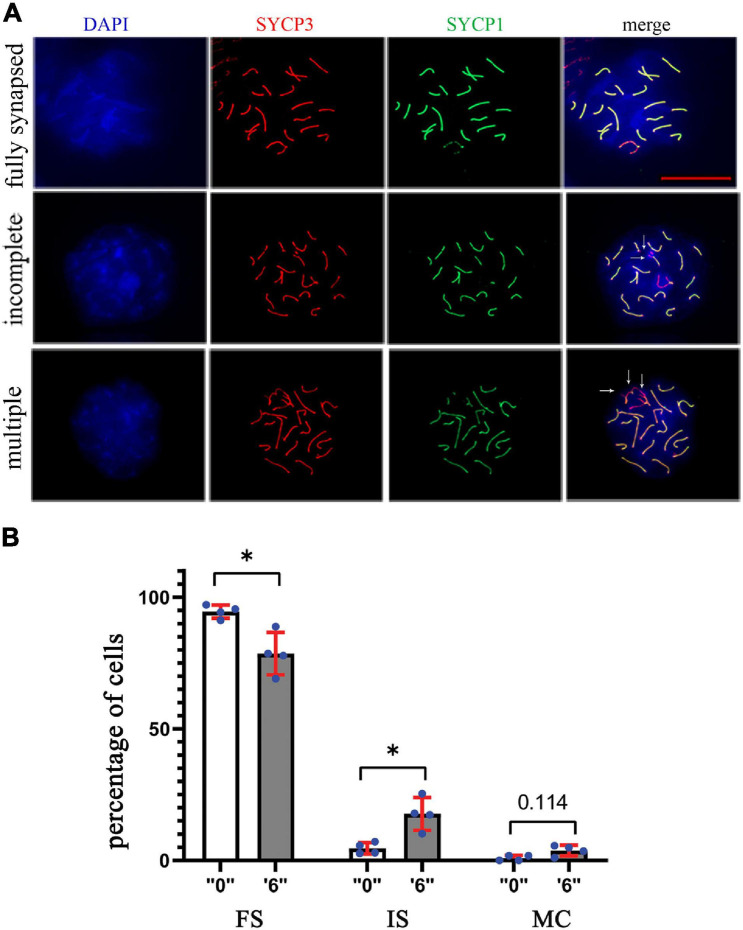
The synapsing defects increased in *thia*-exposed male mice. **(A)** The meiotic spreads from “0” and *thia* dose “6” were stained using SYCP1 (a marker of synapsis) and SYCP3 (a marker of meiotic chromosomes), 63X magnification, scale bar represents 20 μm. Chromosomes were scored and categorized as fully synapsed (first row), incomplete synapsing (second row, marked by arrows), and multiple connections (third row, marked by arrows). **(B)** Quantitative analyses showed a significant decrease in fully synapsed chromosomes and an increase in incomplete synapsing in the treated group compared to the control (FS, fully synapsed; IS, incomplete synapsing; MC, multiple connections). We counted a minimum of 75 cells for the analyses in control and *thia* dose “6” groups. (^∗^*p* < 0.05, Mann–Whitney pairwise comparisons). Error bars represent standard deviation.

In order to assess the synapsing efficiency between the control and treatment groups, we immunostained the surface spread slides against SYCP3 (meiotic chromosome marker), and SYCP1 (a marker of totally synapsed chromosomes) proteins in dose “6” (Figure 6A). SYCP1 and SYCP3 were analyzed for a minimum of 75 *pachytene* cells from control and exposed groups. Compared to the control group, we observed 3.8 times increase in incomplete synapsed chromosomes at a dose of 6 mg/kg/day (Figure 6B). Multiple connections when more than one chromosome was connected were detected 4.2 times more in the exposed group.

In conclusion, gestational exposure to *thia* leads to persistence of DSBs in sex chromosomes, induces telomere and synapsing defects.

### The End-to-End Telomere Connections in Exposed Mice Are Associated With Decreased TERF1 Protein

**FIGURE 7:**
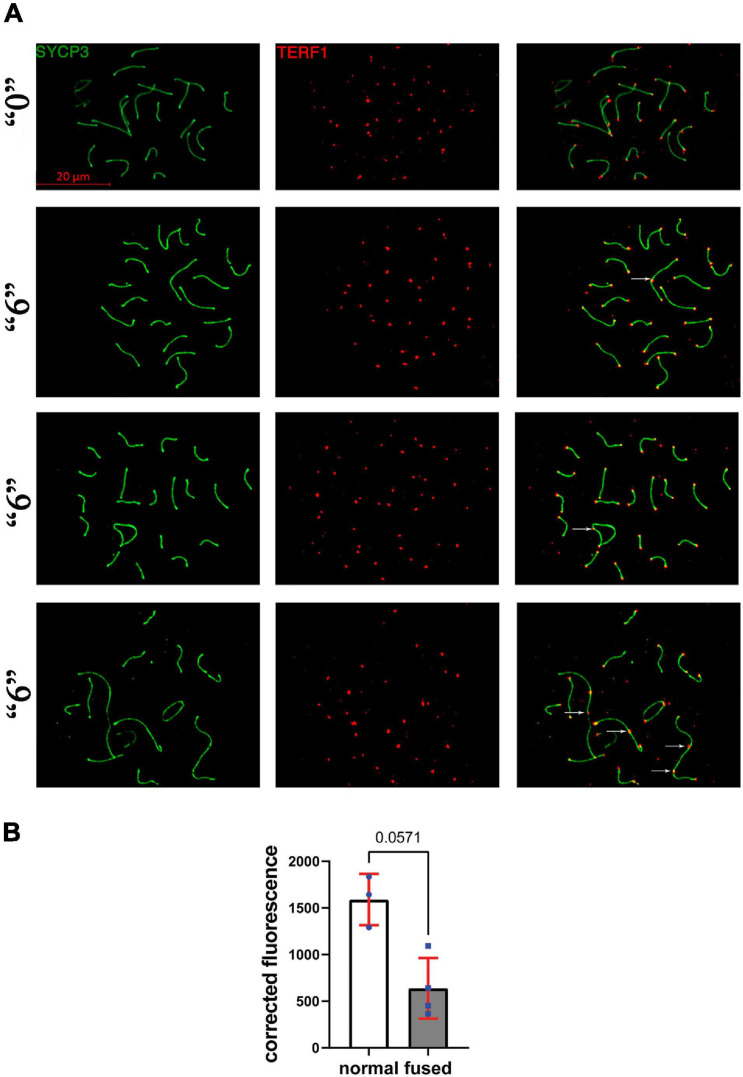
TERF1 in *thia*-exposed male mice was decreased in *thia* exposed animals compared to control animals. **(A)** Telomeres in *thia*-exposed male mice were visualized by TERF1 (telomeres, red) and SYCP3 (chromosomes, green) immunostaining, scale bars represent 20 μm. **(B)** Quantification of TERF1 fluorescence signal associated with the chromosome axes (merged image) showed that TERF1 had decreased in fused chromosomes in the “6” dose treatment group (*n* = 4) compared to control group (*n* = 3). For the analyses, we counted all the TERF1 foci that are associated with the chromosome axes in a minimum of 20 cells per group, *p < 0.05 Mann–Whitney test, error bars represent standard deviation.

Since we observed several synapsing defects in the treated groups, including telomere-to-telomere connections and abnormal expression of several telomere maintenance-related genes, we asked whether telomere-associated proteins such as TERF1 have altered function at telomeric ends. To this end, we immunostained the surface spreads against SYCP3 and TERF1 (Figure 7A). TERF1 staining appeared as a dot at each chromosome end. We performed a quantitative analysis of the intensity of TERF1 fluorescence in all telomeres in cells with fused chromosomes. We observed an average decrease of 2.5-times of TERF1 at telomeres in fused chromosomes of *thia* exposed animals compared to control group, *p* = 0.057, Mann–Whitney test (Figure 7B).

In conclusion, gestational exposure to *thia* affects telomere TERF1 level in exposed males, which could be accountable for telomere connection defects.

### Genes Deregulated in *thia*-Exposed Animals Encode for Proteins Implicated in Translation, ATP Synthesis and Cytoskeleton Morphology

**FIGURE 8:**
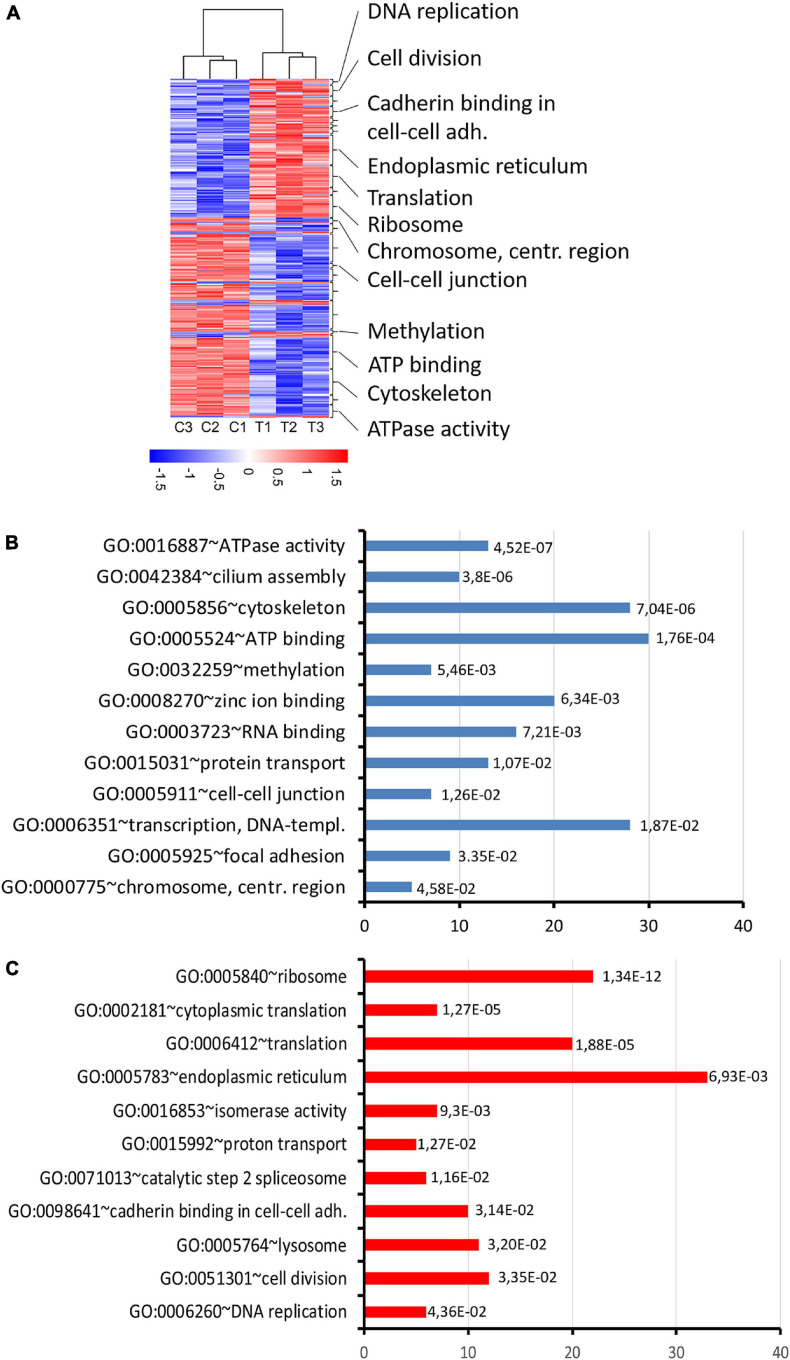
RNA expression changes in *thia-*derived adult males. **(A)** A heatmap of the selected differentially expressed genes. The hierarchical cluster analysis segregated the groups according to their expression levels in the testis fractions. Red indicates high expression and blue indicates low expression. **(B)** Gene ontology of down-regulated genes and **(C)** up-regulated genes. Gene ontology terms were ranked based on *p*-value, abscissa axis shows the number of the genes in the cluster.

To reveal the molecular mechanisms of *thia* action, we performed the transcriptomic analysis of mRNA using paired-ends stranded RNA sequencing using three biological replicates for *thia*-treated and control test. To reveal *thia* effects, we performed analyses on RNA extracted from testes of mice exposed to highest dose of 6 mg/kg/day and compared it to RNA extracted from the control group. We identified 598 differential transcripts that correspond to 560 genes (FC > 1.5 and FDR < 0.05) (Figure 8A, Supplementary Table 3, and Supplementary Figure 8). Among differentially expressed genes (DEGs) we identified 235 genes that were down-regulated and 363 that were up-regulated. We performed the functional annotation of the down-regulated and up-regulated genes separately by using the gene ontology program DAVID as described in the section “Materials and Methods.” We noted that among down-regulated genes the strongest enrichment was found in gene clusters related to ATPase activity, ATP-binding, and cilium and cytoskeleton functions (Figure 8B and Supplementary Table 4). Most of ATPase activity-associated genes are ABC transporters (*Abcb9, Abcb8, Abca9, Abca17, Abca15*). ABC transporters use ATP for energy to translocate substrates across membranes. The ATP-binding gene cassette combines a wide number of genes (Chen et al., 2016). In our study we identified that among down-regulated DEGs, there are also several ATP-binding genes: chromatin remodeling factor, *Smarca2*, dyneins (*Dnah6, Dnah10, Dnah17, Dync2h1, Dync1h1*) and tubulins (*Tll4, Tll5, Tll8*). The decrease in expression of genes requiring ATP for their function suggests that energy production in the form of ATP was affected by *thia* exposure. We also determined that several centromere-related genes (*Phf2, Ppp2r5c, Cenpf, Tnks, Cenpe*), which are important for the proper formation of the mitotic spindle, were downregulated. The up-regulated genes are overrepresented in the cluster related to ribosome and protein translation functions (Figure 8C and Supplementary Table 5), suggesting that protein translation was affected in *thia*-exposed mice. Notably, genes associated with DNA replication (*Ppld44, Cdc45, Blm, Nol8, E4f1, Rpa3*) and cell division (*Ccne2, Ppp1ca, Gnai2, Fbxo5, Cetn2, Ube2i, Pmf1, Ccna2, Sept9, Terf1*) were among up-regulated genes.

Next, we performed the functional annotation of all DEGs using the Kyoto Encyclopedia of Genes and Genomes (KEGG) pathway database. This analysis revealed that the genes implicated in Parkinson’s disease (*Atp5a1, Ndufa7, Ndufb2, Ndufs8, Park7, Cox7a2, Cox7c, Cox6b1, Cycs, Uchl1*) were upregulated in *thia*-exposed mice (*p*-value = 4.2E-3) (Supplementary Table 6). Most of these genes encode for the proteins implicated in the mitochondrial respiratory chain, which produces energy in the form of ATP. Specifically, *thia* affects the respiratory complex 1 enzyme, NADH dehydrogenase, which consists of large number of subunits and is encoded by several genes, including, *Ndufb2, Ndufs8*, which were up regulated in our study. *Thia* also affects the terminal complex IV enzyme, Cytochrome C Oxidase, which is composed of subunits that are encoded by several genes, including *Coxa7a2, Cox7c, Cox6b1*, which were up-regulated in our study. Our analysis suggests that *thia* exposure activates enzymes of the respiratory chain, which could impact numerous cellular processes.

We observed changes in the genes encoding for proteins essential for the chromatin structure regulation. Specifically, the genes controlling histone H3 trimethylation at lysine 4, such as *Setd1b1* were down-regulated and the genes implicated in histone SUMOylation (*Cdkn2a* and *Sumo3*) were up-regulated. Notably, genes encoding telomere-maintenance proteins, *Blm, Tinf2, Cdc45, Ccne2*, and *Terf1*, were also up-regulated, suggesting that *thia* exposure affects telomere function as well.

We performed GSEA analysis (Supplementary Figure 9). GSEA analysis revealed several significant gene ontology terms in our RNA-seq data. Among upregulated genes there is enrichment in gene ontology term known as “ciliary function” and many downregulate genes were combined by gene ontology term “chemical homeostasis.”

Finally, to reveal the origin of DEGs, we performed comparison of our DEGs with genes preferentially expressed in each testis cell type. Our analysis revealed that the majority (51.8%) of the DEGs are expressed in several cell types of the germline (Supplementary Figure 10). However, 26.8% of the DEGs are normally highly expressed in spermatogonia (*Sox3*, *Uchl1, Cited1, Gadd45g, Sohlh1, Ezh1, Taf8, Phb2*), suggesting that spermatogonia are strongly affected by exposure to *thia*. These genes encode for transcription factors implicated in cell differentiation.

Gene marker analysis showed that there is a possible effect of *thia* on the increase in spermatocyte population. However, the most of differentially expressed genes identified by RNA-seq analysis are highly expressed in spermatogonia, implying that cell composition does not dramatically contribute to RNA-seq data alteration.

To conclude, gestational exposure leads to a global change in the expression of protein translation, ATP-dependent and chromatin-modifying genes.

### Effects of *thia* Exposure on H3K9me3 and H3K4me3

**FIGURE 9:**
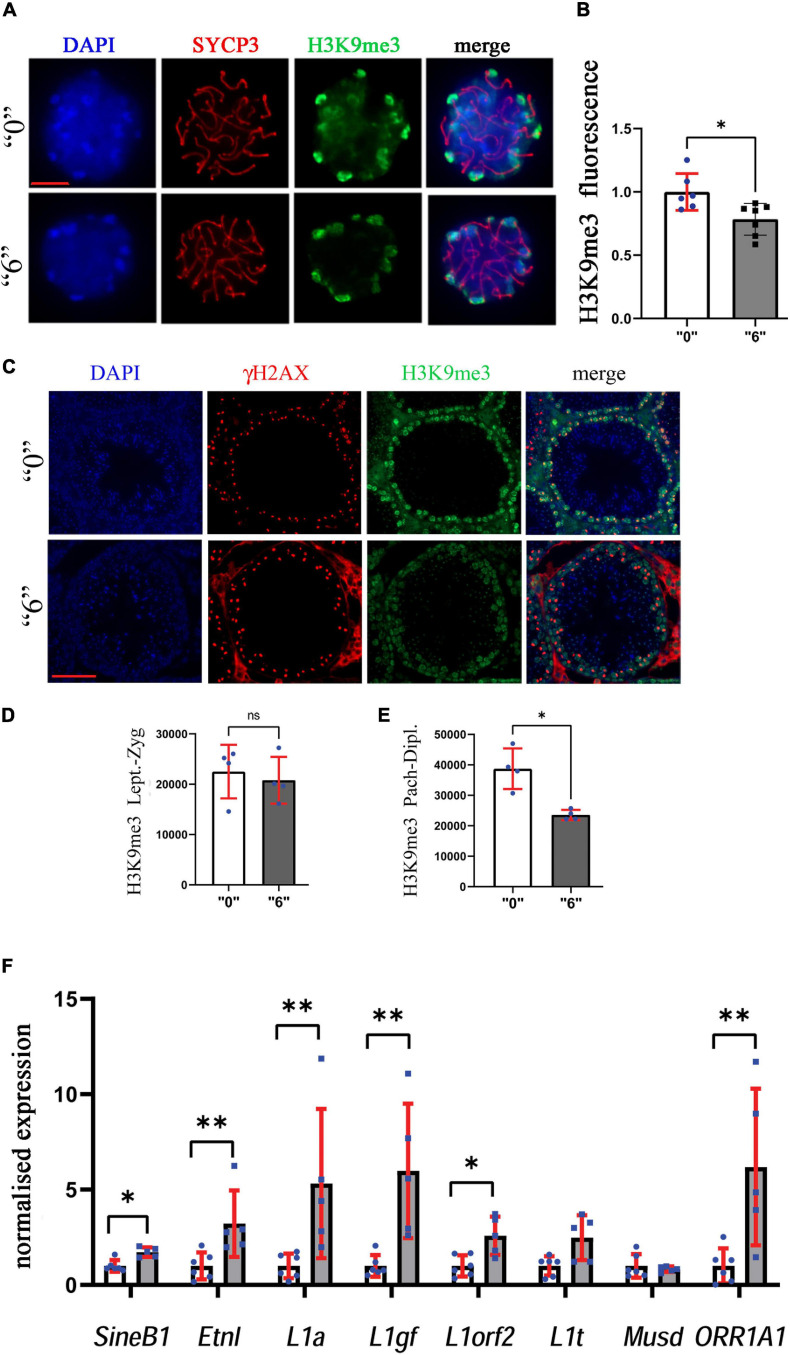
Heterochromatin organization is perturbed in exposed mice. **(A)** The structurally preserved nuclei (SPN) were immunostained by using antibodies against H3K9me3 (green) and SYCP3 (red), scale bar represents 10 μm. We counted at least 15 cells for each replicate four independent biological replicates for “0” and treated group “6.” **(B)** The analyses showed a significant decrease between the “0” group (*n* = 7) and *thia* dose “6” treatment group (*n* = 7), ^∗^*p* < 0.05, non-parametric Mann–Whitney test, error bars represent standard deviation. **(C)** H3K9me3 (green) was also analyzed in testis sections of 35-days-old mice to identify differences in meiotic cell-specific expression pattern by co-staining them with γH2AX (red, 40X magnification, scale bar 50 mm). We counted minimum 20 cells in four sections for four biological replicates per group. Quantitative analysis of H3K9me3 in **(D)** *leptotene-zygotene* and **(E)** *pachytene-diplotene* stages. **(F)** Quantitative analysis of retroelement gene expression in *thia*-exposed male mice. mRNA was extracted and analyzed by RT-qPCR using primers (Supplementary Table 5) for retroelements. Gene expression was normalized to the housekeeping gene, *Rpl37a*. Six biological replicates were used for each group, ^∗^*p* < 0.05, ^∗∗^*p* < 0.01, non-parametric Mann–Whitney test, error bars represent standard deviation.

Since we observed alterations in histone modifying enzymes in our RNA-Seq data, we decided to evaluate the effects of *thia* on epigenetic marks important for meiosis such as H3K9me3. To this end, we prepared structurally preserved nuclei, immunostained them against H3K9me3 and SYCP3, and performed z-stack acquisition followed by deconvolution of the images (Figure 9A). The normalized values for H3K9me3 were 22% lower in exposed mice compared to control mice (Figure 9B). In cells from control animals, H3K9me3 was visible at pericentromeric regions; conversely, in treated cells, H3K9me3 staining appeared lower in intensity.

To confirm our analysis and to reveal the global effects of H3K9me3 on testis, we performed immunofluorescence analysis on testis sections (Figure 9C). To distinguish meiotic cell types, we co-stained the sections against γH2AX, and performed the analysis in *leptotene-zygotene* and *pachytene-diplotene* cells. We did not observe significant changes in the *leptotene-zygotene* stage (Figure 9D), but we detected a decrease in H3K9me3 intensity in *pachytene-diplotene* cells by 38% (Figure 9E). This change is consistent with the changes that we observed in structurally preserved nuclei.

We analyzed H3K9me3 histone fractions as well. We chose also to analyze H3K4me3 as this mark has an opposite effect compared to H3K9me3 and is associated with gene transcription activation. Western blotting of purified histone proteins from whole testis revealed no significant alteration in the protein levels of H3K9me3 and a decrease in H3K4me3 in the *thia*-treated group when compared to the control (Supplementary Figured 11A,B).

Since H3K9me3 is essential for the control of endogenous retrovirus activity, we analyzed the transcription activity of major retroelements. To this end, we extracted mRNA from adult male testes and analyzed the RNA expression of major classes of retroelements in 35d.o. males. We found that expressions of retroelements increased dramatically, up to 5.3 times for L1a, 6 times for L1gf, 6.2 times for ORR1A1 and 3.2 times for EtnI (Figure 9F). Our data suggest that retrotransposons were activated in *thia*-exposed male testis.

Our data show that the chromatin structure is altered upon *thia* treatment in adult testis tissue, which could lead to changes in meiotic progression and in the gene expression.

## Discussion

*Neonics* insecticides have been increasingly used in recent years due to their perceived favorable safety profiles. However, *neonics* are generally considered to be developmental neurotoxicants. In this study, we aimed to reveal the effects of a widely used insecticide, *thiacloprid*, on male meiosis. We chose to expose mice to *thia* gestationally since this developmental period is crucial for germline epigenetic reprogramming. We tested several low doses of *thia*. Doses were chosen based on log_10_ increases from the daily authorized dose (0.06 mg/kg/day) of another *neonic*, imidacloprid, established by the French Agency for Food, Environmental and Occupational Health and Safety (ANSES). According to the EPA the no-observed-adverse-effect-level (NOAEL) dose for *thiacloprid* is 27.3 mg/kg/day for females and 102.6 milligram/kilogram/day (mg/kg/day) for male mice, suggesting that the dose we used was below the NOAEL. However, the exposure during embryonic time could be more toxic even with a low dose. The highest dose used in our study was more than 4 times lower than NOAEL dose for females. For *thia*, we found that all tested doses cause a reduction in the spermatozoa number. The decrease in spermatozoa number could be explained by the vulnerability of germline cells following gestational exposure. In our previous study, where we assessed the effects of an endocrine-disrupting compound, *chlordecone*, we observed that a low dose of 0.1 mg/kg/day causes a dramatic decrease in spermatozoa; this dose was also below NOAEL (Gely-Pernot et al., 2018). While the mechanism of action of *thiacloprid* and *chlordecone* are different, both compounds have endocrine-disrupting capacity; so gestational exposure to these compounds even at low doses could lead to dramatic changes later in life. Endocrine-disrupting chemicals (EDCs) affect nuclear receptors, which, in turn, regulate many target genes. Indeed, *thia* affects the thyroid hormonal system (Sekeroglu et al., 2014), which also plays an essential role in testis development. It is suggested that thyroid hormone receptors detected in embryonic testes could act directly on testis cells via regulation of steroidogenesis in Leidig cells (Wagner et al., 2008). Thus, exposure to *thia* could lead to a decrease of spermatozoa also via hormonal pathways.

We observed the density of cells to be higher in testis, with an increase in both meiotic and post meiotic cells. We believe that the observed synapsing meiotic defects could lead to delay in further progression and the accumulation of pachytene cells in seminiferous tubules. The increase in cell fraction could be due to alteration in microtubule motor activity. We observed a low expression of dynein encoding genes in all cell fractions. Dynein proteins are involved in the movement of chromosomes and positioning the mitotic spindles during cell division (Banks and Heald, 2001), thus their decreased expression could impact both meiotic and post meiotic cells.

In adult males, we found that many ATP-dependent genes were down-regulated. This suggests that *thia* exposure could perturb the electron transfer and the production of ATP, which could affect every process where ATP is required, including protein translation, transport, DNA repair and cell cycle.

In our study, many cellular processes being affected including cell cycle, mitotic spindle formation, and DNA repair could explain the decrease in spermatozoa number.

Notably, we identified genes that are implicated in neurogenerative diseases such as in Parkinson and Alzheimer diseases. We suggest that the deregulation of acetylcholine receptor network could be one of the possible reasons for the alterations in the expression of genes implicated in these diseases. However, future studies are required to confirm this.

We also detected that the most DEGs are normally highly expressed in spermatogonia. The molecular network in spermatogonia begin to establish after cell lineage specification during embryonic development. We believe that exposure to *thia* during critical window could affect embryonic gonocytes and those alterations could be preserved until adulthood, ultimately leading to alterations of spermatogenesis.

We observed that *thia* exposure during development affects the DNA chromatin organization in adults. The altered epigenetic organization of heterochromatin coupled with activation of retroelements suggests that changes initiated in embryos could play a deleterious role in adult male meiosis. Several studies suggest that mechanisms establishing H3K9me3 marks are vulnerable. Indeed, it has been shown that environmental factors could affect H3K9me3 marks. For example, under anoxia conditions, the enzymes introducing these marks were up-regulated in freshwater turtles (Wijenayake et al., 2018). Moreover, arsenic exposure significantly increased the H3K9me3 levels in rat testes (Alamdar et al., 2019). Temperature-induced expression of endogenous repressed repeats lead to altered H3K9me3 and these changes were inherited for 14 generations in *C. elegans* (Klosin et al., 2017). The different results for H3K9me3 by Western blotting and immunofluorescence could be explained by the analyzing only spermatocytes by immunofluorescence and entire testis for Western blotting. Furthermore, we noted that several genes encoding epigenetic histone modifying enzymes were deregulated, including genes associated with histone H3K4 trimethylation and SUMOylation, suggesting that there are global effects of *thia* exposure on epigenetic modifications. Further work will be required to address the direct role of *thia* in these mechanisms.

Apart from this, we observed an increased expression of retroelements in adult males. In embryonic gonads most of the epigenetic methylation and histone modification marks are erased during somatic-to-germline-transition. *De novo methylation* in males is reestablished at embryonic days 14.5-17.5 *post coitum* (pc) and continues after birth (Kota and Feil, 2010). DNA methylation depends upon the activities of DNA methyltransferases (DNMTs). Loss of function of methyltransferase DNMT3L leads to infertility due to an “inappropriate euchromatin” state during meiosis, including transcriptional activation of retrotransposons (Bourc’his and Bestor, 2004). Moreover, LINE1 expression by itself could introduce a high level of double strand breaks (Gasior et al., 2006), suggesting that endonuclease activity of expressed LINE elements could contribute to DSB formation and repair in germline cells. We observed the decrease in H3K9me3 in spermatocytes and, since DNA methylation and histone methylation are functionally linked (Cheng, 2014), it is conceivable that both mechanisms could be deregulated and lead to overexpression of retroelements. Further work is required to establish the direct connection.

## Conclusion

Our study revealed that gestational exposure to *thiacloprid* induces an alteration in chromatin organization, causes meiotic defects and affects gene expression, which altogether leads to a reduction in spermatozoa numbers. Since *thiacloprid* is in use in many countries, and the current daily acceptable dose causes undesirable effects if exposed gestationally, our data is important for further evaluation of *thiacloprid* safety.

## Data Availability Statement

The datasets presented in this study can be found in online repositories. The names of the repository/repositories and accession number(s) can be found below: Gene Expression Omnibus, accession: GSE171985.

## Ethics Statement

The animal study was reviewed and approved by Ethics Committee of the Ministry of the Research of France, agreement number: 17473-2018110914399411.

## Author Contributions

FS: conceptualization, supervision, methodology, investigation, and writing-original draft preparation. CH: formal analysis, investigation, and writing-review and editing. LL: software, validation, formal analysis, investigation, and writing-review and editing. SD’C: conceptualization, supervision, methodology, investigation, and writing-review and editing. MC and P-YK: investigation. GL: software, validation, and formal analysis. ST: writing-review and editing. All authors contributed to the article and approved the submitted version.

## Conflict of Interest

The authors declare that the research was conducted in the absence of any commercial or financial relationships that could be construed as a potential conflict of interest.

## Footnotes

1 http://www.bioinformatics.babraham.ac.uk/projects/fastqc/
