# Supplementary material for: Epigenetic Effects Promoted by Neonicotinoid Thiacloprid Exposure
Source: Front Cell Dev Biol. 2021 Jul 6;9:691060. doi: 10.3389/fcell.2021.691060 (PMC8290843; doi:10.3389/fcell.2021.691060)
Supplement: Supplementary file 1 [file Data_Sheet_1.pdf]

Colin Hartman[1]*, Louis Legoff[1]*, Martina Capriati[1]*, Gwendoline Lecuyer[1], Pierre-Yves Kernanec[1], Sergei Tevosian[2], Shereen Cynthia D'Cruz[1#] and Fatima Smagulova[1#]

[1] Univ. Rennes, EHESP, Inserm, Irset (Institut de recherche en santé, environnement et travail) - UMR_S 1085, F-35000, Rennes, France
[2] University of Florida, Department of Physiological Sciences Box 100144, 1333 Center Drive, 32610, Gainesville, FL, USA

***These authors equally contributed**
# Corresponding authors

Shereen Cynthia D'Cruz shereen-cynthia.d-cruz-benard@inserm.fr, Irset-Inserm UMR 1085, 9 avenue du Prof. Léon Bernard, 35000 Rennes, France.

Fatima Smagulova, fatima.smagulova@inserm.fr, Irset-Inserm UMR 1085, 9 avenue du Prof. Léon Bernard, 35000 Rennes, France.

Epigenetic effects promoted by neonicotinoid thiacloprid exposure.

Supplementary information

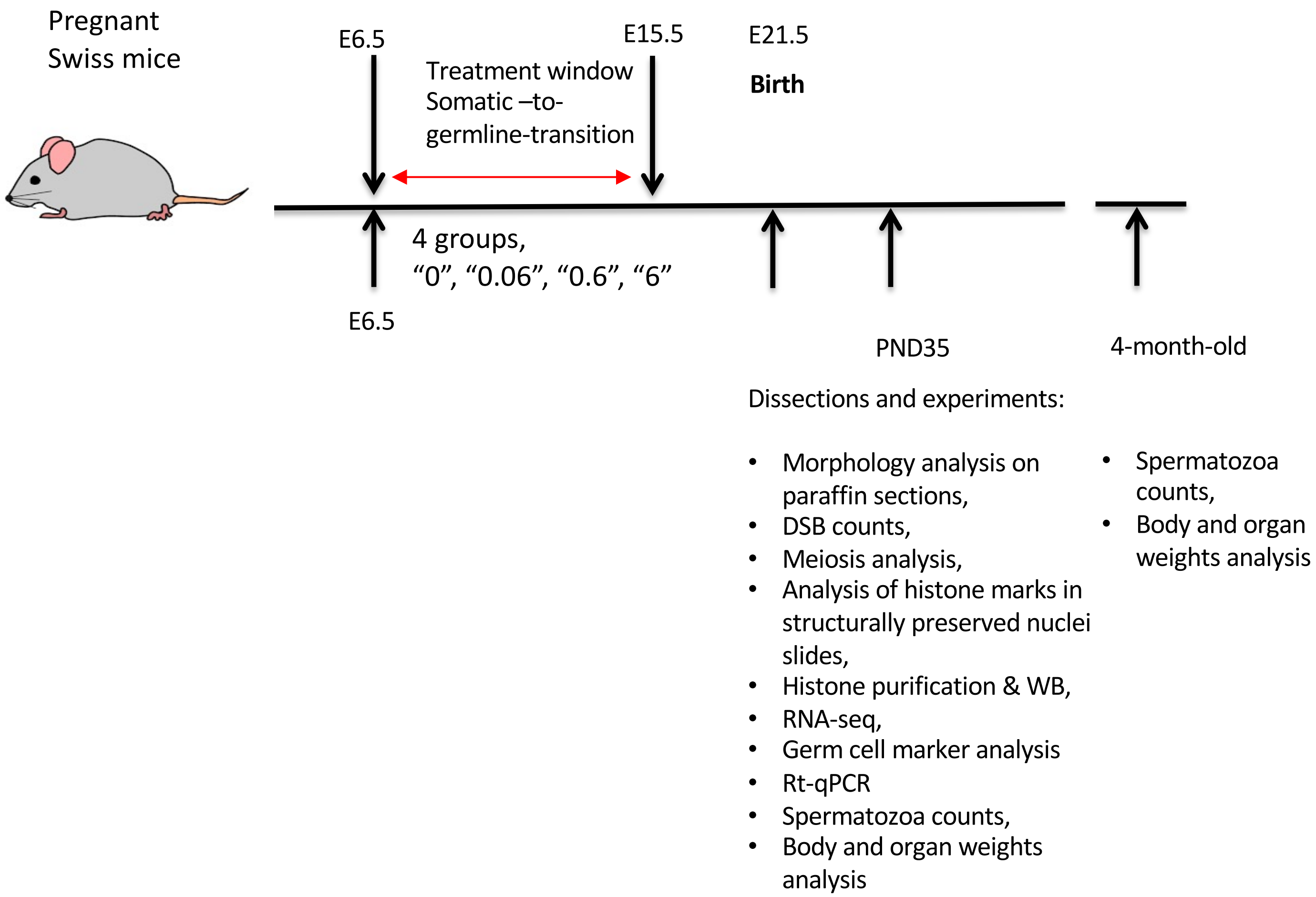

Figure S1. Schematic presentation of experiments. Pregnant outbred Swiss mice were treated with *thia* doses 0, "0.06", "0.6", "0.6", "6" mg/kg/day, control mice received only vehicle (oil). Mice were sacrificed at postnatal day 35 and at age of 4 months.

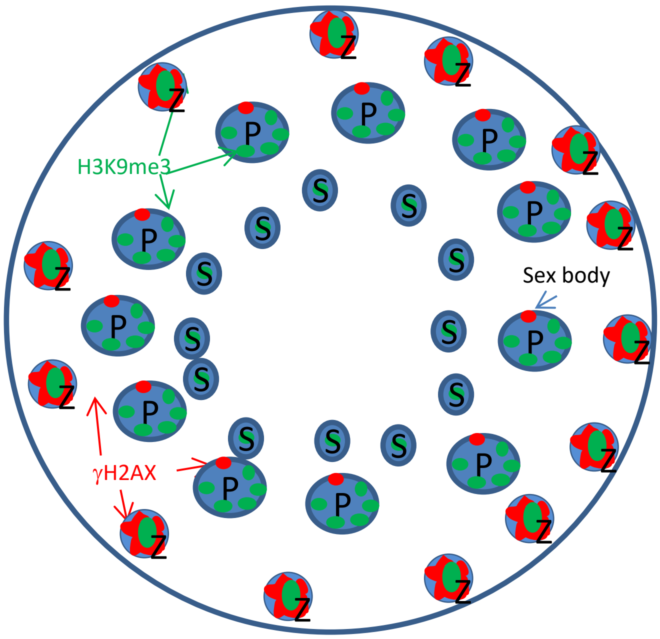

Figure S2. Schematic representation of germ cells in seminiferous tubule, Z, *leptotene-zygotene*, P, *pachytene-diplotene,* S, spermatids. Red color indicates pattern of gamma γH2AX staining, a marker of double-strand DNA breaks. γH2AX appears as strong staining all over the nucleus in *leptotene-zygotene* cells whereas in *pachytene-diplotene* stages, γH2AX stays as dots only on sex chromosomes.

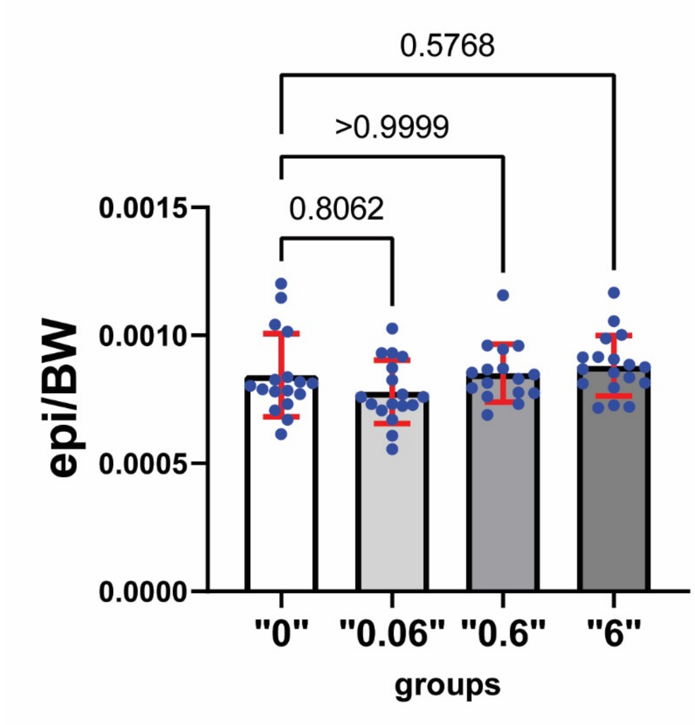

Figure S3. Epididymis-to-body weight ratio in 35-day-old mice. No significant changes in relative epididymis-to-body weight were observed, n=10 for each group, *p<0.05, Kruskal-Wallis test. Error bars represent standard deviation.

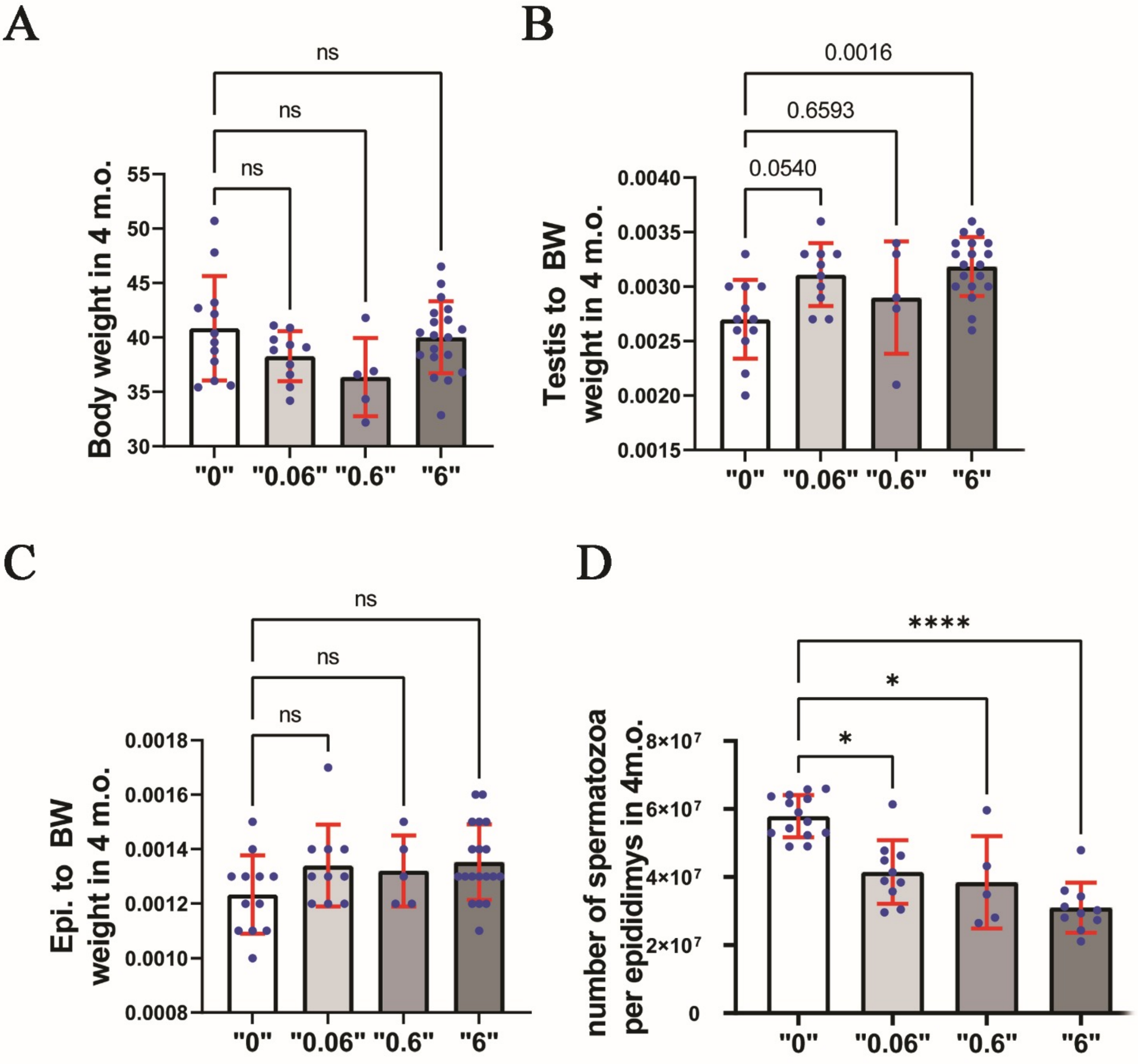

Figure S4. Body weight (BW) (A), Testis-to-BW ratio, (C) Epididymis-to-BW ratio, (D) spermatozoa numbers in 4-month-old mice. No significant changes in body weight and relative epididymis-to-body weight were observed. Relative testis to body weight showed a significant decrease at '0.06' and '6' doses of thia compared to contol. We observed a significant reduction in spermatozoa number at all the *thia* doses tested. (*p<0.05, **p<0.01, ***p < 0.001, Kruskal-Wallis test, Mann-Whitney pairwise comparisons). n= 10, Error bars represent standard deviation.

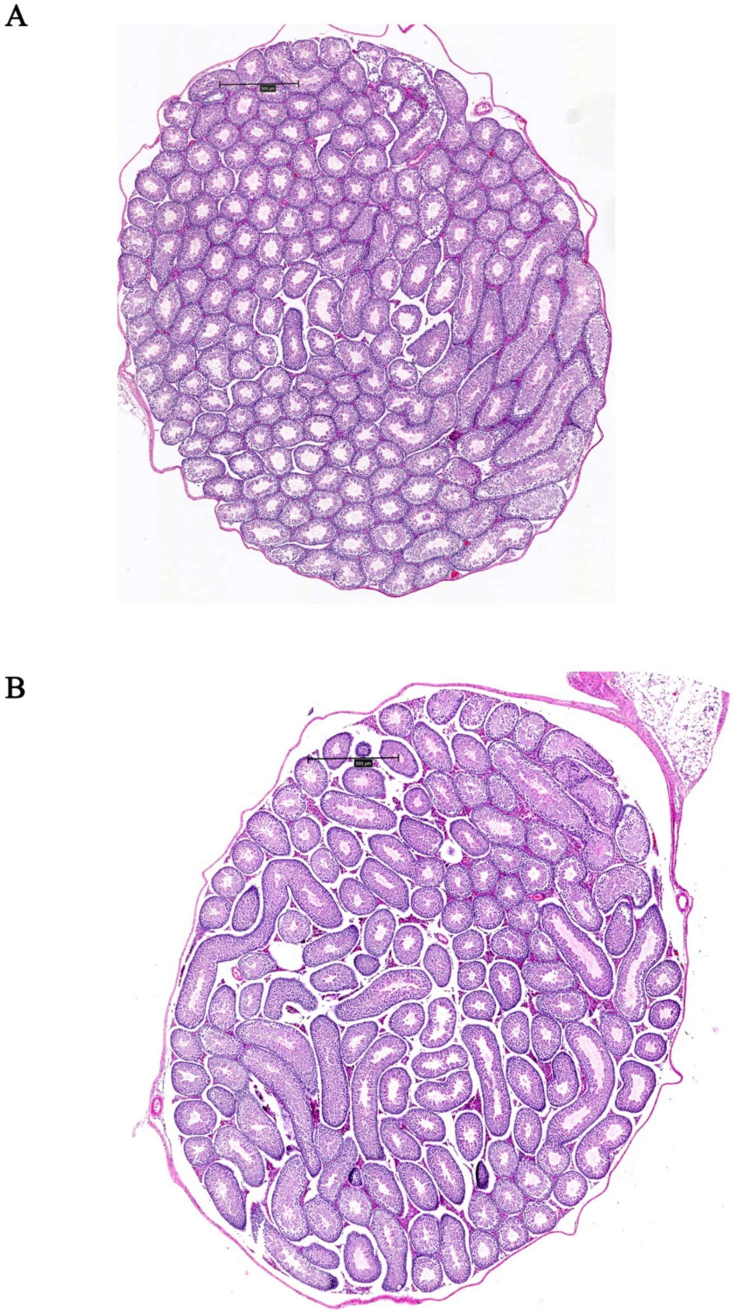

Figure S5. Representative H&E stained images from (A) control and *thia* “6” testis at 5X magnification. Scale bar 500 μm. Image shows elongated as well and circular tubules in both groups.

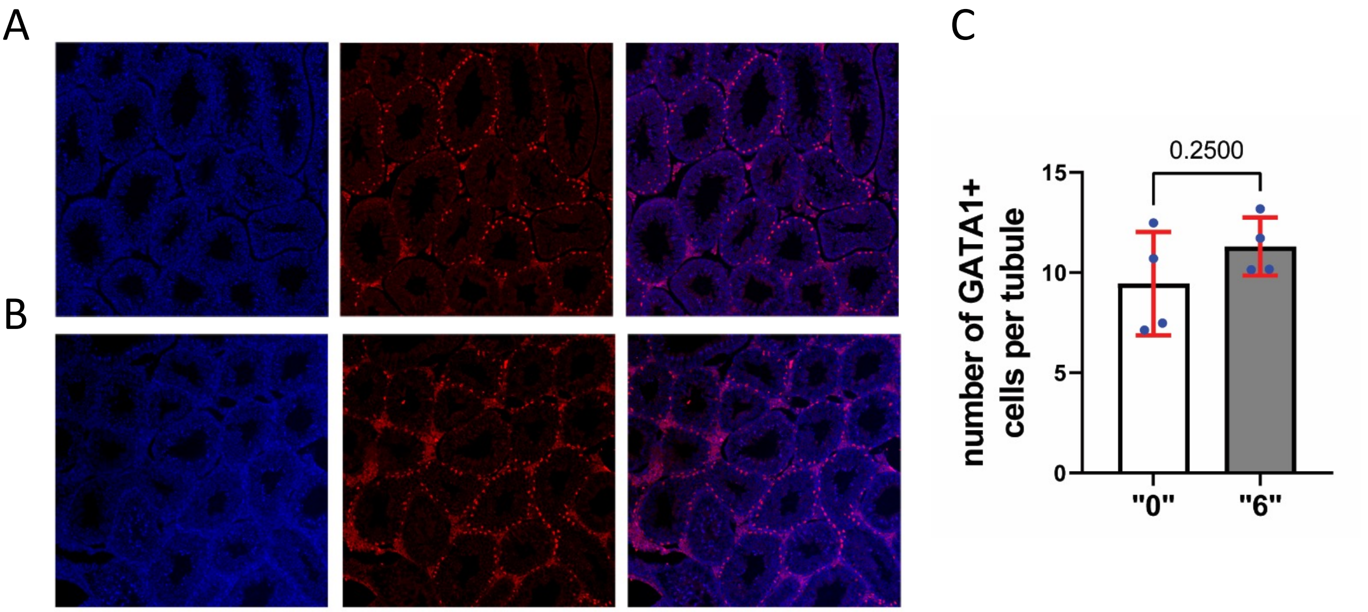

Figure S6. Immunofluorescence analysis of GATA+ positive cells (red) in “0” (A) and *thia ”6”* (B) treated testes, (C) The numbers of GATA+ positive cells per tubules. We counted a minimum of 20 sections for the analyses, 4 biological replicate per group. *p<0.05, Mann-Whitney test. Error bars represent standard deviation.

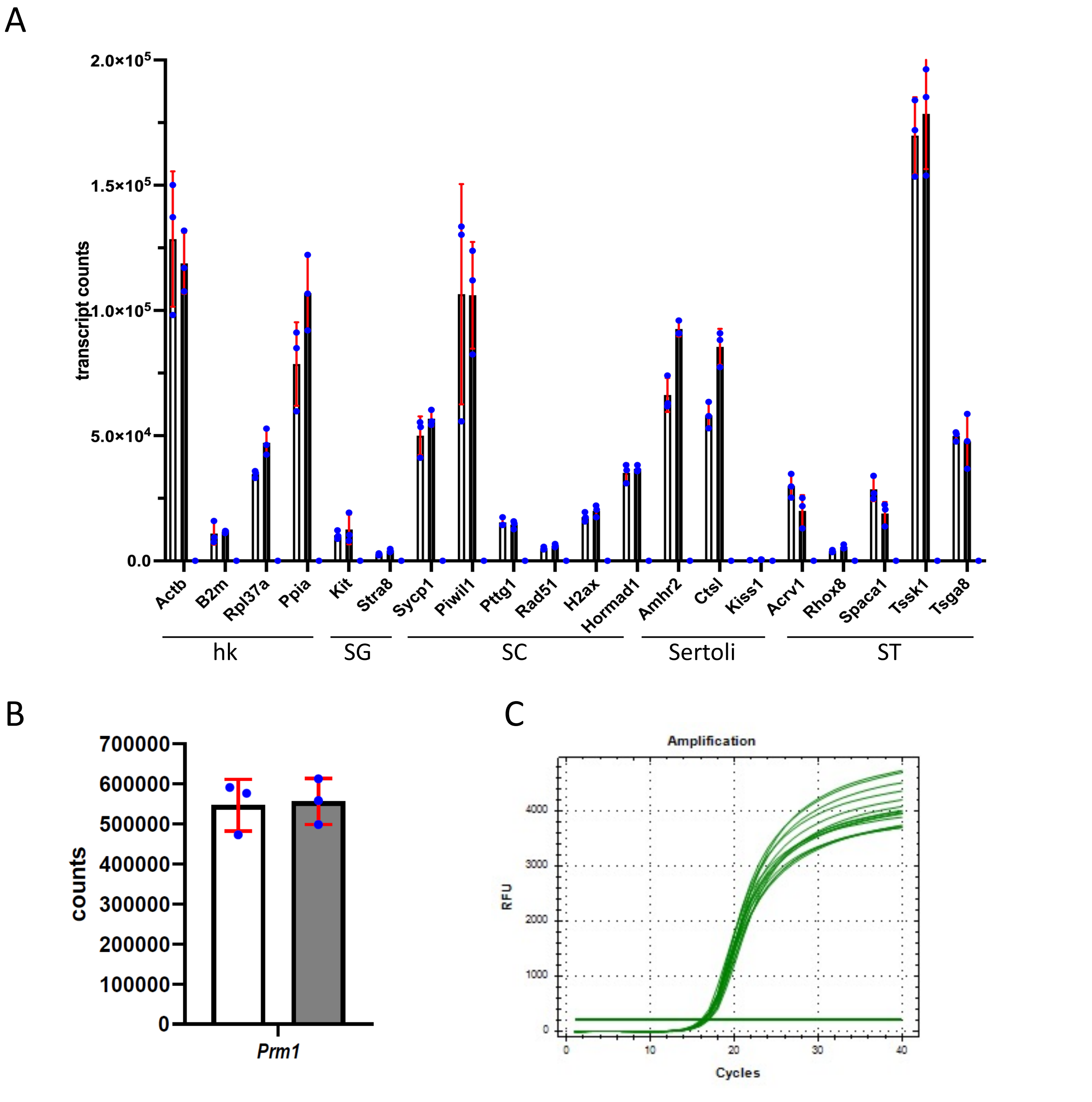

Figure S7. Transcript counts from RNA-seq analysis. Transcript counts were extracted from feature counts RNA-seq analysis. Counts for control are presented in white column and for *thia* "6" in grey. HK, housekeeping genes, SG, spermatogonia, SC, spermatocyte, ST, spermatid, (B) Counts for *Prm1* are presented separately due to very high number of counts, 3 replicates were used for each group. Most of the tested genes passed the RNA-seq cutoff FC>1.5. (C) Rt-qPCR amplification curve for *Rpl37a* gene, from control and *thia* "6" showed close Cq (cycle quantification) values. 6 replicates were used for each group.

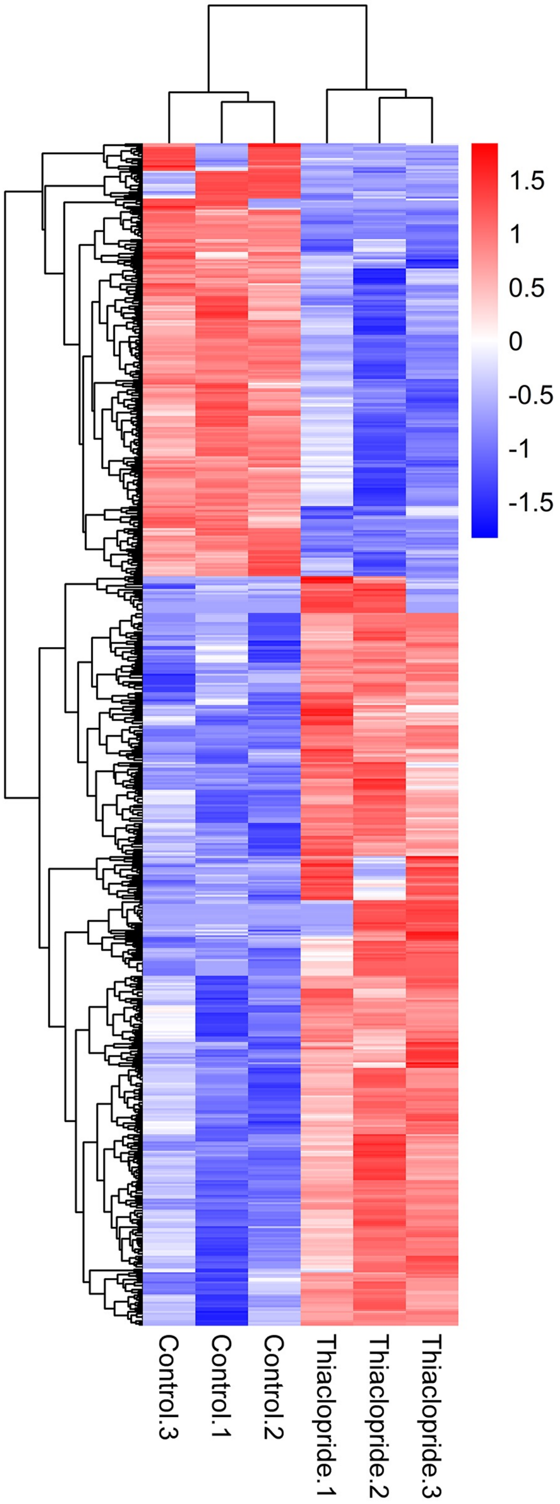

Figure S8. Heatmap of all DEGs (FC > 1.5; FDR < 0.05). Clustering was done using Euclidian distance and complete hierarchical clustering.

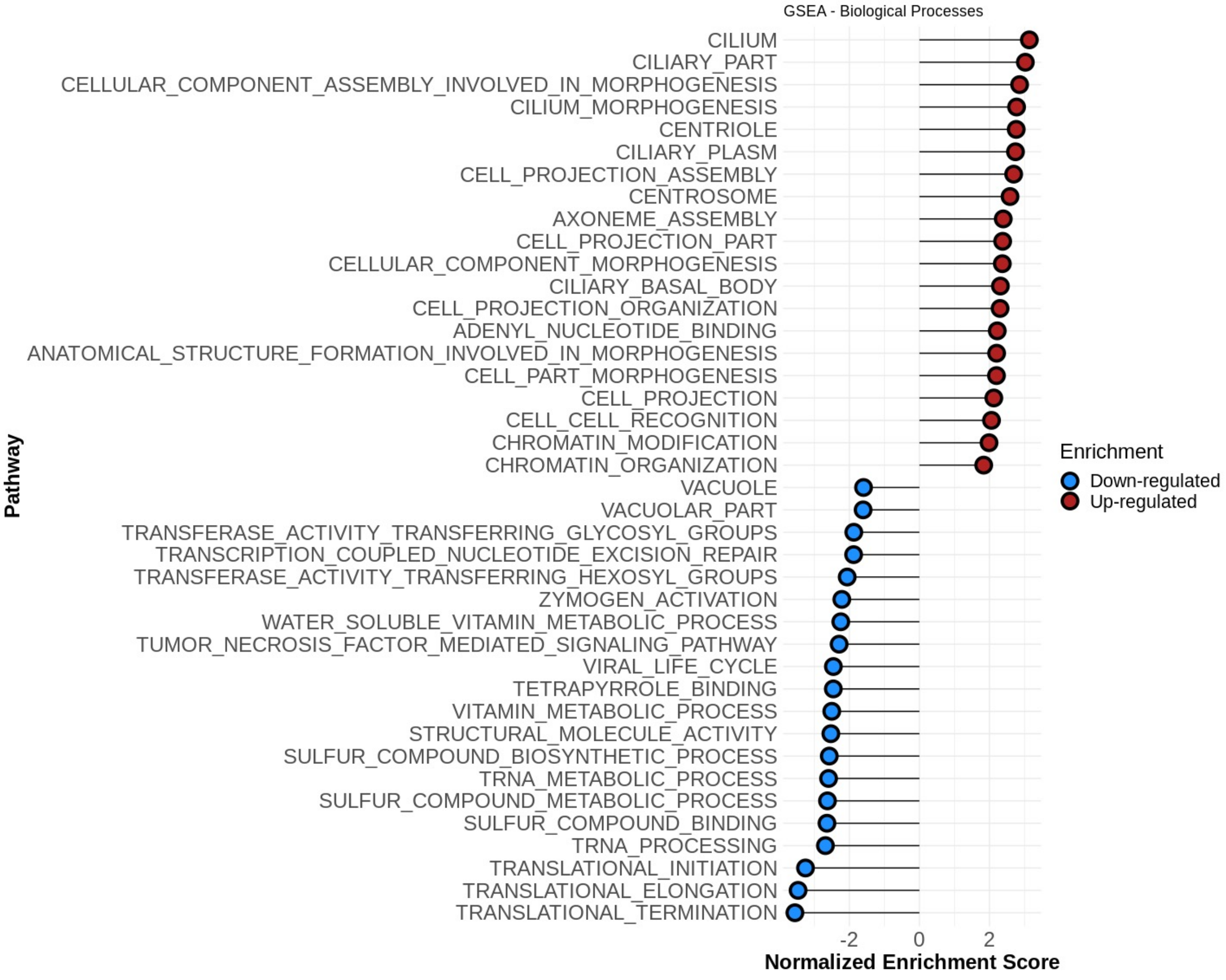

Figure S9. Gene Set Enrichment Analysis was performed with fGSEA tool and gage tool (R package) on genes dataset from “0” and *thia* “6” groups (adj p-val = 0.05) in 35 day-old murine testis tissue. The genes are ranked by FC and we selected GO process found commonly by both tools. The graph represent the set of GO process enriched genes and the normalized enrichment score (NES) for each GO process. The upregulated pathways are presented in red and the downregulated pathways are presented in blue.

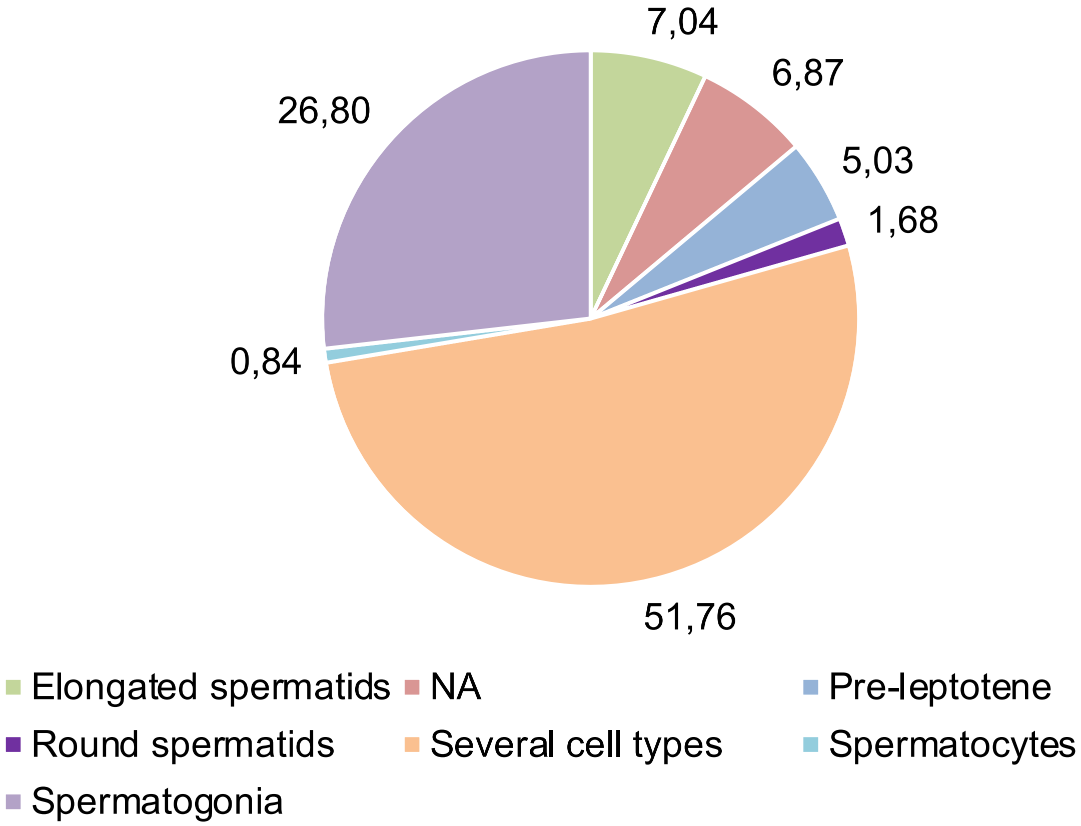

Figure S10. DEGs distribution in testis cell types revealed that majority of DEGs are normally expressed in spermatogonia.

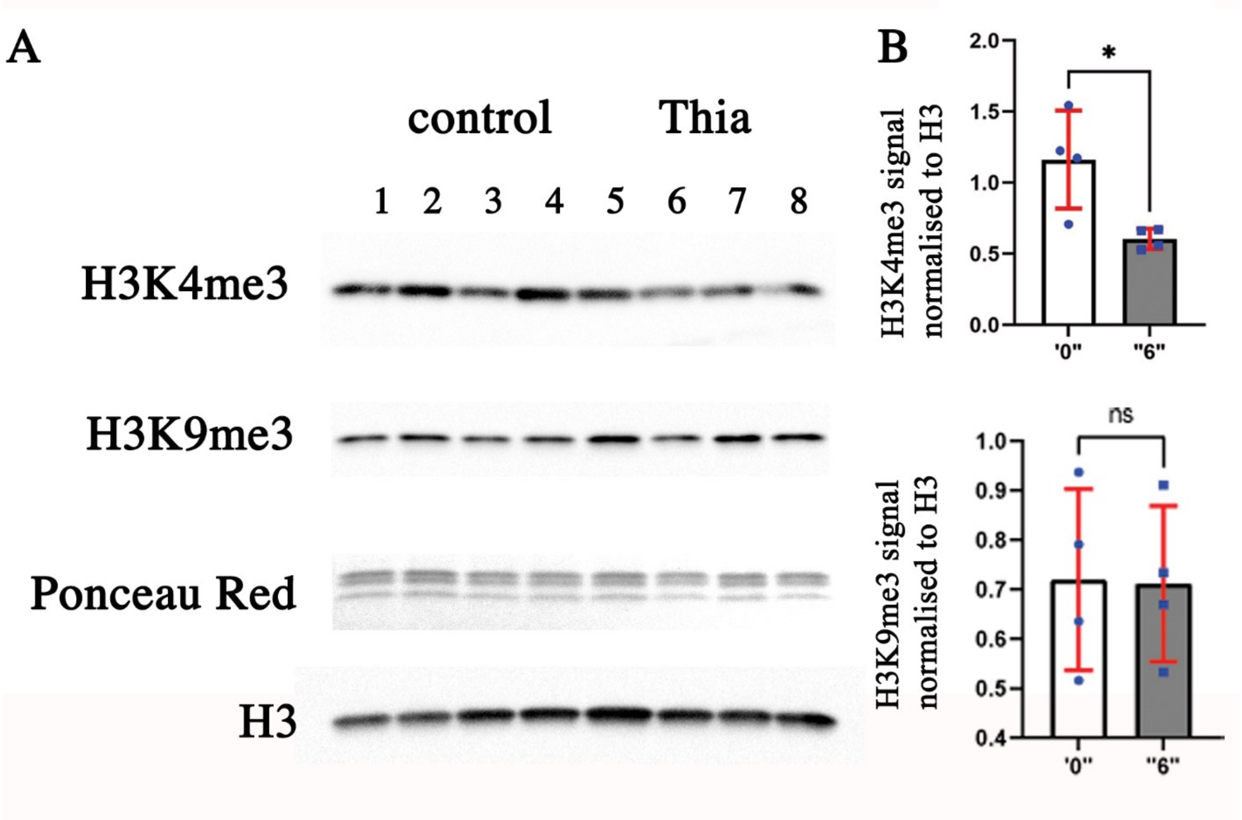

Figure S11. The gestational exposure to *thia* leads to changes in global levels of histones H3K4me3 and H3K9me3 levels in testis. Histones were extracted from testes and equal amounts were loaded on the gel and the proteins were transferred to membranes. Membranes were blotted against H3K9me3 and H3K4me3 antibodies. The signal intensities were calculated by using Fiji program, H3K4me3 and H3K9me3 levels were normalized to total histones stained by unmodified histone H3, n=4 for each group, *$p<0.05$, nonparametric Mann-Whitney test. Error bars represent standard deviation.

Supplementary Table 1. Primers used in this study for RT-qPCR

gene	forward	reverse	
Stra8	GGAGAAAAAGGCCAGACTCC	CCACGTCAAAAGCATCTTCA	SG
Kit	AGCGTCTTCCGGCACAACGG	AGCGTCTTCCGGCACAACGG	SG
Hormad1	ATAGCATCCAAGCATCCACTTCAC	AGAGACCAGGATAAGAAACGCAG	SC
H2afx	GGCCATCCGCAACGACG	TTCTTGCCGACCGCCG	SC
Piwil1	ACGACGATCAGGGAGTGACC	CACCGTCTCCTGACCTCGTG	SC
Pttg1	GGTGGCGCAGTCTTCGAGTA	ACCAGTGCCCAGCTTCAACC	SC
Sycp1	GGAAGATGTGGAAAAGAATAATGATA	AATAACATGGATTGAAGAGACTTTCG	SC
Rad51	ATTGGTTCCAATGGGTTTCA	GGCATGTAACAGCCAACGTA	SC
Tsga8	TGTTCCGACTTCATCCACCGA	TGGTCTTCGCACCCTTTCCAC	RS
Prm1	GCCAGCACCATGGCCAGATA	GACGGCAGCATCTTCGCCTC	RS
Spaca1	AGCTGAGCTCTATGAGGTGCG	TCCTGTGCCTACCAACCACC	RS/ES
Tssk1	GATGACGCTGCCGTCCTCAA	TCTGAGGGGGCTTTCTTGCG	ES
Rhox8	CAGGAGCACCGTCAACAGCAT	CATGCAGCTGCGTCATCGCTT	Sertoli
Ctsl	GCTAGCCGCCTCAGGTGTTT	TTCCACTGGTGCCACTCTGC	Sertoli
Amhr2	GCACCTTCTTCCAAGGAAGCCT	CCGCAGGAAGCAGTGTCCAA	Sertoli
Kiss1	CGGACCCCAGGAACTCGTTA	GGCATGGCGACGACCTAC	Sertoli
Rpl37a	TGGGGCCTGGACCTACAA	GCAGGGCTTCTACTGGTCTT	house-keeping
Etnl[1]	GTTAAACCCGAGCGCTGGTTC	GCTATAAGGCCCAGAGAGAAATTT	retro
SineB1[1]	TGGTGGTGCATGCCTTTAAT	CCTGGTGTCCTGGAACTCACT	retro
ORR1A1[1]	CTTTAGTTGATGGCCCAGGA	CCAACTCTGCCCTCTGTAGC	retro
MusD1[1]	GTGGTATCTCAGGARGAGTGC	GGGCAGCTCCTCTATCTGAGTG	retro
L1-A[2]	GGATTCCACACGTGATCCTAA	TCCTCTATGAGCAGACCTGGA	retro
L1-T[2]	CAGCGGTCGCCATCTTG	CACCCTCTCACCTGTTCAGACTAA	retro
L1-Gf[2]	CTCCTTGGCTCCGGGACT	CAGGAAGGTGGCCGGTTGT	retro
L1-ORF2[2]	GGAGGGACACTTCATTCTCATCA	GCTGCTCTTGTATTTGGAGCATAGA	retro

1. Hao C, Gely-Pernot A, Kervarrec C, et al. Exposure to the widely used herbicide atrazine results in deregulation of global tissue-specific RNA transcription in the third generation and is associated with a global decrease of histone trimethylation in mice. Nucleic Acids Res. 2016;44(20):9784-9802.
2. Barau J, Teissandier A, Zamudio N, et al. The DNA methyltransferase DNMT3C protects male germ cells from transposon activity. *Science*. 2016;354(6314):909-912.

Supplementary Table 2. Number of sequencing reads per sample in RNA-seq data

Testis RNA "0"-1	177766240
Testis RNA "0"-2	221434816
Testis RNA "0"-3	216378446
Testis RNA "6"-1	210538858
Testis RNA "6"-2	254563194
Testis RNA "6"-3	221968566

Supplementary Table 3. All differentially expressed genes

Chr	Start	End	Strand	Transcript ID	Gene Name	baseMean	log2FoldChange	pvalue	padj	Origin
chr1	5083110	5095728	+	ENSMUST00000194676.5	*Atp6v1h*	874,99	0,71	3,02E-04	1,14E-02	Multiple
chr1	6214645	6276648	+	ENSMUST00000027040.12	*Rb1cc1*	13761,90	-1,55	4,29E-27	6,27E-24	Multiple
chr1	15805664	15843470	+	ENSMUST00000027057.7	*Terf1*	1081,99	13,44	5,84E-04	1,86E-02	Pre-leptotene
chr1	16665210	16678275	+	ENSMUST00000065373.5	*Tmem70*	3816,23	1,07	1,64E-03	3,89E-02	Multiple
chr1	20996299	21079229	-	ENSMUST00000037998.5	*Tram2*	3066,55	-0,78	4,01E-07	4,53E-05	Multiple
chr1	34436670	34439672	-	ENSMUST00000042493.9	*Ccdc115*	5564,16	1,21	2,52E-10	5,17E-08	Multiple
chr1	36422065	36445271	-	ENSMUST00000125304.7	*Lman2l*	1863,75	-0,69	3,15E-05	1,91E-03	Pre-leptotene
chr1	36792191	36939527	-	ENSMUST00000027290.11	*Tmem131*	7596,15	2,13	1,47E-03	3,60E-02	Pre-leptotene
chr1	37430139	37438984	+	ENSMUST00000114925.9	*Unc50*	2444,71	0,76	2,75E-04	1,06E-02	Pre-leptotene
chr1	39367842	39371911	+	ENSMUST00000086535.11	*Rpl31*	14875,74	0,76	2,53E-04	9,95E-03	Elongated Spermatids
chr1	43123290	43196984	-	ENSMUST00000185893.1	*Fhl2*	3245,61	1,04	1,00E-09	1,86E-07	Multiple
chr1	43746966	43827800	-	ENSMUST00000126008.7	*Uxs1*	4864,33	-1,49	1,13E-07	1,47E-05	Spermatogonia
chr1	44055952	44061936	-	ENSMUST00000168641.1	*Gm8251*	5180,70	-1,12	9,56E-04	2,66E-02	Elongated Spermatids
chr1	46425592	46807476	+	ENSMUST00000189749.6	*Dnah7c*	32344,65	-0,71	1,68E-04	7,28E-03	Multiple
chr1	74661745	74703730	+	ENSMUST00000042125.14	*Ttll4*	3423,80	-1,37	1,06E-13	4,13E-11	Multiple
chr1	82724919	82752387	+	ENSMUST00000073025.11	*Mff*	3389,75	1,44	5,18E-05	2,86E-03	Multiple
chr1	82735470	82752158	+	ENSMUST00000160744.1	*Mff*	3442,14	-15,31	1,33E-37	4,04E-34	Multiple
chr1	83254139	83408200	-	ENSMUST00000160953.7	*Sphkap*	8145,02	-0,59	2,15E-03	4,66E-02	Multiple
chr1	87327003	87450796	+	ENSMUST00000027475.14	*Gigyf2*	5924,56	-2,27	5,13E-04	1,70E-02	Multiple
chr1	93685660	93695764	+	ENSMUST00000027499.12	*Bok*	1754,17	0,68	5,38E-04	1,75E-02	Spermatogonia
chr1	105990689	106034067	+	ENSMUST00000119166.7	*Zcchc2*	30409,42	-0,76	1,81E-12	5,37E-10	Multiple
chr1	106171752	106394250	+	ENSMUST00000061047.6	*Phlpp1*	62212,74	-0,62	2,10E-03	4,58E-02	Multiple
chr1	132112237	132139684	-	ENSMUST00000027697.11	*Cdk18*	1766,26	-0,94	2,78E-04	1,07E-02	Elongated Spermatids
chr1	134415456	134433348	+	ENSMUST00000112237.1	*Adipor1*	19795,86	0,59	1,31E-03	3,31E-02	Multiple
chr1	135729147	135752232	+	ENSMUST00000027677.7	*Csrp1*	8290,84	0,80	8,50E-04	2,45E-02	Spermatogonia
chr1	136269160	136345698	-	ENSMUST00000192001.5	*Camsap2*	14684,31	-0,63	2,32E-04	9,28E-03	Multiple
chr1	150361313	150392762	-	ENSMUST00000097547.9	*Odr4*	919,59	13,20	1,54E-10	3,25E-08	NA
chr1	152836995	152902622	-	ENSMUST00000073441.12	*Smg7*	2323,08	-5,31	6,64E-06	5,24E-04	Multiple
chr1	153503015	153550045	-	ENSMUST00000041874.8	*Npl*	4644,97	0,72	5,35E-04	1,75E-02	Multiple
chr1	155844964	155973255	-	ENSMUST00000138762.7	*Cep350*	52240,19	-0,95	1,23E-05	8,81E-04	Multiple
chr1	171217802	171222184	-	ENSMUST00000111327.7	*Tomm40l*	2043,20	0,62	1,52E-04	6,73E-03	Pre-leptotene
chr1	187215743	187350723	+	ENSMUST00000160471.7	*Gpatch2*	4700,05	-1,01	5,83E-09	9,77E-07	Multiple
chr1	189640606	189688086	-	ENSMUST00000171929.7	*Cenpf*	32096,32	-0,84	5,07E-06	4,13E-04	Multiple
chr1	191978251	191986391	+	ENSMUST00000175680.2	*Rd3*	1773,03	-0,72	5,89E-05	3,16E-03	Multiple
chr1	193153111	193172023	+	ENSMUST00000076521.6	*Irf6*	2305,43	-0,62	1,40E-03	3,49E-02	Round Spermatids
chr1	194619825	194816869	+	ENSMUST00000027952.11	*Plxna2*	6856,33	-0,70	2,09E-04	8,62E-03	Multiple
chr2	25842995	25847248	-	ENSMUST00000076989.6	*Sohlh1*	1573,47	0,72	1,13E-08	1,78E-06	Spermatogonia
chr2	28468066	28471178	+	ENSMUST00000038600.3	*Mrps2*	2093,93	-0,59	2,27E-03	4,83E-02	Spermatogonia
chr2	29889895	29931746	+	ENSMUST00000046571.13	*Odf2*	5114,18	-0,62	7,43E-05	3,84E-03	Multiple
chr2	30441853	30447131	+	ENSMUST00000131476.7	*Ptpa*	543,69	1,04	5,99E-11	1,35E-08	NA
chr2	32095528	32110820	+	ENSMUST00000057423.5	*Plpp7*	1375,95	0,77	7,51E-04	2,23E-02	NA
chr2	32308471	32353263	-	ENSMUST00000113352.8	*Dnm1*	3453,66	-0,65	7,44E-06	5,77E-04	Pre-leptotene
chr2	32363198	32378297	+	ENSMUST00000048964.13	*Ciz1*	4379,89	-1,47	4,03E-08	5,71E-06	Multiple
chr2	35109492	35178110	+	ENSMUST00000156933.7	*Cntrl*	5609,38	-4,56	4,32E-20	3,69E-17	Multiple
chr2	35109492	35130727	+	ENSMUST00000028235.10	*Cntrl*	1450,92	-2,67	1,49E-10	3,17E-08	Multiple
chr2	53134704	53191225	-	ENSMUST00000076313.12	*Prpf40a*	10670,50	0,96	6,24E-10	1,21E-07	Multiple
chr2	53138238	53191284	-	ENSMUST00000210789.1	*Prpf40a*	3411,89	-9,01	1,53E-119	4,17E-115	Multiple
chr2	59903185	59912536	-	ENSMUST00000130637.1	*Baz2b*	965,74	-1,09	2,26E-04	9,09E-03	Multiple
chr2	60751953	60881438	-	ENSMUST00000164147.7	*Rbms1*	1026,97	-3,89	1,59E-22	1,79E-19	Multiple
chr2	74697727	74700208	+	ENSMUST00000059272.9	*Hoxd9*	1367,89	0,65	1,23E-04	5,67E-03	Pre-leptotene
chr2	101562124	101621046	-	ENSMUST00000090513.10	*B230118H07Rik*	311,86	-11,85	1,74E-03	4,03E-02	Multiple
chr2	104721784	104742878	-	ENSMUST00000028595.7	*Depdc7*	974,01	0,65	1,32E-03	3,34E-02	Spermatogonia
chr2	120567671	120601255	+	ENSMUST00000110711.8	*Snap23*	899,22	-2,29	1,87E-03	4,25E-02	Multiple
chr2	121449383	121451054	+	ENSMUST00000099475.11	*Serf2*	9454,60	0,93	4,33E-05	2,48E-03	Multiple
chr2	122781999	122809553	+	ENSMUST00000110506.8	*Sqor*	8230,64	7,91	1,93E-07	2,37E-05	NA
chr2	125086568	125123626	-	ENSMUST00000152367.7	*Myef2*	2842,95	4,54	2,82E-35	6,79E-32	Multiple
chr2	125087742	125123660	-	ENSMUST00000067780.9	*Myef2*	5005,04	-3,46	5,00E-09	8,60E-07	Multiple
chr2	125087742	125123661	-	ENSMUST00000147105.7	*Myef2*	6839,37	-0,87	3,37E-04	1,24E-02	Multiple
chr2	130667610	130681614	+	ENSMUST00000103193.4	*Itpa*	8794,14	0,69	3,44E-04	1,26E-02	Spermatogonia
chr2	143947395	144011263	-	ENSMUST00000016072.11	*Rrbp1*	30703,31	-0,59	1,78E-03	4,09E-02	Multiple
chr2	144594054	144595366	+	ENSMUST00000136628.1	*Smim26*	367,12	0,60	1,62E-03	3,86E-02	NA
chr2	148871722	148875576	-	ENSMUST00000028938.6	*Cst3*	38698,58	0,73	8,92E-06	6,69E-04	Multiple
chr2	152576086	152580312	-	ENSMUST00000053180.3	*Defb19*	25873,61	0,71	6,83E-04	2,08E-02	Multiple
chr2	152604327	152612729	+	ENSMUST00000058086.5	*Defb36*	6539,24	0,79	2,50E-06	2,24E-04	Multiple
chr2	155819832	155826925	-	ENSMUST00000029142.14	*Eif6*	16494,17	0,64	1,89E-03	4,29E-02	Spermatogonia
chr2	158409951	158497646	+	ENSMUST00000109485.8	*Ralgapb*	4262,96	28,50	2,97E-13	1,07E-10	Spermatogonia
chr2	164354070	164356507	-	ENSMUST00000109367.9	*Slpi*	243,51	-5,25	4,67E-04	1,59E-02	NA
chr2	164613522	164618212	+	ENSMUST00000094346.2	*Wfdc6b*	1138,98	0,68	1,67E-03	3,92E-02	Multiple
chr2	164656046	164657368	+	ENSMUST00000094344.5	*Wfdc10*	7225,20	0,78	1,82E-04	7,75E-03	Multiple

Supplementary Table 3. All differentially expressed genes (*continued*)

Chr	Start	End	Strand	Transcript ID	Gene Name	baseMean	log2FoldChange	pvalue	padj	Origin
chr2	164832873	164840019	+	ENSMUST00000103093.9	*Ctsa*	3875,99	1,07	6,68E-05	3,51E-03	Spermatogonia
chr2	165280786	165287835	-	ENSMUST00000156134.7	*Slc35c2*	259,44	-4,14	1,77E-04	7,58E-03	Spermatogonia
chr2	180036374	180042433	-	ENSMUST00000029082.8	*Psma7*	6987,71	0,77	3,60E-04	1,31E-02	Spermatogonia
chr2	180257377	180258445	+	ENSMUST00000059080.6	*Rps21*	14526,85	0,75	7,61E-04	2,25E-02	Spermatogonia
chr2	181357833	181365404	-	ENSMUST00000127988.7	*Arfrp1*	1130,95	1,32	2,07E-03	4,54E-02	Spermatogonia
chr3	34077279	34081321	-	ENSMUST00000108195.9	*Dnajc19*	1686,23	0,98	2,28E-03	4,85E-02	Multiple
chr3	36564865	36572150	-	ENSMUST00000029270.9	*Ccna2*	3254,98	0,60	5,27E-06	4,27E-04	Spermatogonia
chr3	36870028	36992727	+	ENSMUST00000211820.1	*4932438A13Rik*	9714,63	-0,75	8,99E-12	2,29E-09	Pre-leptotene
chr3	51416614	51442863	+	ENSMUST00000192419.5	*Naa15*	758,89	-1,33	1,11E-09	2,08E-07	Multiple
chr3	84444035	84479491	-	ENSMUST00000091002.7	*Fhdc1*	4609,00	-3,40	4,49E-04	1,54E-02	Elongated Spermatids
chr3	88394143	88410331	-	ENSMUST00000056370.12	*Pmf1*	3296,25	0,72	1,60E-04	7,02E-03	Spermatogonia
chr3	89266552	89267077	+	ENSMUST00000040824.1	*Dpm3*	1199,09	0,87	2,05E-03	4,51E-02	Spermatogonia
chr3	90006220	90052472	-	ENSMUST00000195995.4	*Ubap2l*	1253,30	2,15	1,33E-11	3,32E-09	Multiple
chr3	90511034	90514392	-	ENSMUST00000060738.8	*S100a1*	1396,13	1,08	1,06E-03	2,87E-02	Pre-leptotene
chr3	93555080	93564643	+	ENSMUST00000045756.13	*S100a10*	3189,53	0,94	3,50E-05	2,09E-03	Pre-leptotene
chr3	94612759	94658872	-	ENSMUST00000196733.4	*Tuft1*	999,29	-11,08	7,13E-06	5,58E-04	Multiple
chr3	95988429	95996001	-	ENSMUST00000015889.9	*Plekho1*	1715,36	-3,99	5,48E-04	1,77E-02	Elongated Spermatids
chr3	98805505	98811108	-	ENSMUST00000170847.1	*Hsd3b6*	6389,83	6,77	2,19E-06	2,00E-04	Multiple
chr3	104789034	104794344	+	ENSMUST00000106787.7	*Rhoc*	439,03	12,13	1,90E-03	4,29E-02	Spermatogonia
chr3	107926334	107931817	-	ENSMUST00000004137.10	*Gstm7*	15072,36	0,62	7,36E-04	2,19E-02	Elongated Spermatids
chr3	116594982	116621602	+	ENSMUST00000198386.4	*Sass6*	2584,91	-0,77	5,17E-04	1,71E-02	Multiple
chr3	117781871	117868880	-	ENSMUST00000169812.7	*Snx7*	661,22	0,75	7,11E-04	2,13E-02	Multiple
chr3	122538725	122619715	-	ENSMUST00000162409.7	*Fnbp1l*	11088,14	-1,20	4,58E-04	1,57E-02	Spermatogonia
chr3	135212537	135273611	+	ENSMUST00000062893.11	*Cenpe*	22877,19	-0,78	7,72E-05	3,95E-03	Multiple
chr3	135439211	135466675	+	ENSMUST00000197134.2	*Ube2d3*	1030,98	10,47	1,22E-06	1,19E-04	Multiple
chr3	145118620	145153889	+	ENSMUST00000029920.14	*Odf2l*	800,37	-1,96	2,94E-04	1,12E-02	Pre-leptotene
chr3	146457202	146493845	-	ENSMUST00000197980.4	*Spata1*	672,59	-1,57	1,80E-03	4,13E-02	Multiple
chr4	3938888	3943523	+	ENSMUST00000108386.7	*Chchd7*	341,76	0,64	2,27E-04	9,12E-03	Multiple
chr4	11191354	11204675	+	ENSMUST00000170901.7	*Ccne2*	5480,08	0,97	8,68E-06	6,55E-04	Spermatogonia
chr4	41194313	41275144	-	ENSMUST00000108068.7	*Ubap2*	15840,16	3,68	1,93E-03	4,34E-02	Multiple
chr4	41638144	41640324	-	ENSMUST00000127306.1	*Enho*	844,54	0,70	1,29E-03	3,27E-02	Spermatogonia
chr4	41657498	41695442	-	ENSMUST00000102961.9	*Cntfr*	1390,34	-4,38	2,67E-04	1,04E-02	Multiple
chr4	42294267	42294855	+	ENSMUST00000108002.3	*Gm21953*	723,60	7,22	4,78E-09	8,21E-07	NA
chr4	42318334	42323929	+	ENSMUST00000178192.1	*Fam205a3*	4860,67	-1,39	1,15E-03	3,02E-02	NA
chr4	43381979	43427088	+	ENSMUST00000035645.11	*Rusc2*	2936,75	-2,46	3,82E-04	1,37E-02	Multiple
chr4	43493362	43495921	+	ENSMUST00000030181.11	*Ccdc107*	1951,61	0,64	2,11E-03	4,60E-02	Pre-leptotene
chr4	43562332	43567060	+	ENSMUST00000102944.10	*Creb3*	6647,63	0,68	4,01E-05	2,33E-03	Multiple
chr4	46450902	46472657	+	ENSMUST00000102926.4	*Anp32b*	9901,09	4,99	6,54E-19	4,91E-16	Spermatogonia
chr4	47474658	47483242	+	ENSMUST00000065678.5	*Sec61b*	4805,20	0,88	1,88E-03	4,28E-02	Spermatogonia
chr4	59581645	59618688	+	ENSMUST00000107528.7	*Hsdl2*	7205,40	-11,25	7,42E-09	1,21E-06	Multiple
chr4	62190995	62207889	-	ENSMUST00000220873.1	*Zfp37*	3366,30	-2,20	1,29E-21	1,34E-18	Multiple
chr4	70216856	70410443	-	ENSMUST00000144099.7	*Cdk5rap2*	13372,65	3,94	7,92E-21	7,29E-18	Multiple
chr4	70238350	70264680	-	ENSMUST00000138561.7	*Cdk5rap2*	1429,47	2,13	1,41E-07	1,79E-05	Multiple
chr4	72157580	72200862	-	ENSMUST00000107337.7	*Tle1*	1384,01	0,67	9,19E-04	2,59E-02	Spermatogonia
chr4	89137122	89181081	+	ENSMUST00000058030.9	*Mtap*	2246,90	0,59	8,42E-04	2,43E-02	Spermatogonia
chr4	89274541	89294653	-	ENSMUST00000107131.1	*Cdkn2a*	1287,66	0,82	1,95E-04	8,17E-03	Spermatogonia
chr4	107233514	107253532	-	ENSMUST00000030360.10	*Lrrc42*	6543,06	1,78	4,59E-20	3,88E-17	Spermatogonia
chr4	107879755	107887424	+	ENSMUST00000030348.5	*Magoh*	4907,85	0,69	5,34E-05	2,93E-03	Spermatogonia
chr4	109000655	109061777	+	ENSMUST00000102736.8	*Nrd1*	4798,78	28,65	2,24E-13	8,39E-11	Multiple
chr4	109000770	109061161	+	ENSMUST00000106644.8	*Nrd1*	110860,61	-0,88	3,71E-06	3,12E-04	Multiple
chr4	109451098	109476675	-	ENSMUST00000030284.9	*Rnf11*	5198,63	0,95	2,65E-04	1,03E-02	Spermatogonia
chr4	115000159	115043196	+	ENSMUST00000030490.12	*Stil*	8636,95	-0,62	2,28E-13	8,47E-11	Multiple
chr4	116033907	116053380	-	ENSMUST00000165493.7	*Nsun4*	38902,58	-1,01	2,74E-04	1,06E-02	Elongated Spermatids
chr4	116075269	116097043	+	ENSMUST00000030471.8	*Lrrc41*	13022,40	-3,70	5,46E-06	4,39E-04	Multiple
chr4	116819904	116821707	-	ENSMUST00000055436.4	*Hpdl*	939,54	0,91	1,90E-03	4,29E-02	Spermatogonia
chr4	117884326	117887329	-	ENSMUST00000036380.13	*Atp6v0b*	4598,63	0,70	9,36E-07	9,48E-05	Pre-leptotene
chr4	119418530	119422420	-	ENSMUST00000030385.12	*Ppcs*	1748,48	0,77	5,76E-05	3,10E-03	Multiple
chr4	124802678	124804740	+	ENSMUST00000175875.1	*Mtf1*	905,69	-0,65	3,77E-04	1,36E-02	Multiple
chr4	129489014	129491022	-	ENSMUST00000000421.5	*Tssk3*	3274,58	-8,01	1,66E-24	2,05E-21	Elongated Spermatids
chr4	131921771	131923140	+	ENSMUST00000094666.3	*Tmem200b*	3224,72	0,71	1,18E-03	3,08E-02	Spermatocytes
chr4	133266045	133277792	-	ENSMUST00000105907.8	*Tmem222*	7250,47	0,64	9,77E-07	9,83E-05	Multiple
chr4	133480132	133487938	+	ENSMUST00000051676.6	*Tent5b*	3979,90	-2,68	5,77E-30	1,05E-26	NA
chr4	133584362	133591735	+	ENSMUST00000030661.13	*Gpn2*	3810,30	0,59	3,66E-04	1,33E-02	Multiple
chr4	136540354	136549318	+	ENSMUST00000001116.4	*Luzp1*	3758,45	-11,78	3,89E-92	5,32E-88	Multiple
chr4	136880129	136886187	-	ENSMUST00000046384.8	*C1qb*	5850,41	0,73	3,38E-04	1,25E-02	Elongated Spermatids
chr4	136895917	136898803	-	ENSMUST00000046285.5	*C1qa*	6038,60	0,88	3,95E-07	4,47E-05	Spermatocytes
chr4	141467890	141538597	-	ENSMUST00000105786.2	*Spen*	11584,96	-1,14	4,76E-04	1,61E-02	Multiple
chr4	141467890	141538449	-	ENSMUST00000078886.9	*Spen*	22067,27	-0,94	1,82E-05	1,22E-03	Multiple
chr4	143107391	143212705	-	ENSMUST00000105778.7	*Prdm2*	24134,66	-0,61	1,68E-05	1,15E-03	Multiple

Supplementary Table 3. All differentially expressed genes (*continued*)

Chr	Start	End	Strand	Transcript ID	Gene Name	baseMean	log2FoldChange	pvalue	padj	Origin
chr4	147873599	147904704	-	ENSMUST00000030884.9	*Mfn2*	2563,27	-4,32	2,20E-05	1,43E-03	Multiple
chr4	148145788	148152059	-	ENSMUST00000056965.11	*Fbxo6*	1549,10	2,33	1,86E-03	4,23E-02	Multiple
chr4	149649171	149676044	-	ENSMUST00000118704.7	*Pik3cd*	11470,38	-0,62	2,43E-04	9,66E-03	Multiple
chr4	149897967	149909816	-	ENSMUST00000105685.1	*Spsb1*	2303,33	-2,73	5,25E-04	1,72E-02	Round Spermatids
chr4	150897133	150909926	-	ENSMUST00000030805.13	*Park7*	4437,16	0,97	2,91E-05	1,80E-03	Multiple
chr4	152178134	152271852	+	ENSMUST00000075363.9	*Acot7*	37490,51	-5,55	2,79E-05	1,74E-03	Multiple
chr4	152325867	152334071	+	ENSMUST00000139685.7	*Rpl22*	2460,69	-7,59	2,65E-07	3,14E-05	Spermatogonia
chr4	155989466	155992649	-	ENSMUST00000052185.4	*B3galt6*	1753,17	0,78	4,66E-04	1,59E-02	Multiple
chr4_GL456350_random	144079	149877	-	ENSMUST00000115912.2	*AC087559,3*	2739,25	-1,00	1,16E-09	2,15E-07	NA
chr5	8056527	8069151	+	ENSMUST00000148633.3	*Sri*	1282,57	0,93	6,10E-04	1,91E-02	Spermatogonia
chr5	24393676	24410054	+	ENSMUST00000115077.7	*Abcb8*	2198,79	-7,08	5,53E-65	6,48E-61	Multiple
chr5	24394155	24409945	+	ENSMUST00000073076.11	*Abcb8*	5934,43	1,42	2,53E-04	9,95E-03	Multiple
chr5	24802823	24842624	-	ENSMUST00000030787.8	*Rheb*	4664,63	0,95	3,69E-06	3,11E-04	Spermatogonia
chr5	33983433	33985013	+	ENSMUST00000094869.11	*Gm1673*	1891,83	0,71	7,97E-05	4,04E-03	Multiple
chr5	33983470	33985009	+	ENSMUST00000114383.7	*Gm1673*	4140,19	0,63	1,60E-03	3,81E-02	Multiple
chr5	65388378	65390986	-	ENSMUST00000196667.4	*Rpl9*	693,89	1,44	3,36E-04	1,24E-02	Multiple
chr5	66260312	66298015	+	ENSMUST00000031109.6	*Nsun7*	8690,06	-0,65	1,09E-04	5,18E-03	Multiple
chr5	66676091	66687234	+	ENSMUST00000031131.10	*Uchl1*	8617,55	0,65	1,94E-05	1,29E-03	Spermatogonia
chr5	73021211	73148136	-	ENSMUST00000094700.10	*Fryl*	5051,92	-0,96	7,72E-05	3,95E-03	Pre-leptotene
chr5	81660303	81796634	+	ENSMUST00000124117.1	*Adgrl3*	3376,41	-1,09	8,77E-05	4,34E-03	Round Spermatids
chr5	90227166	90366577	-	ENSMUST00000014421.14	*Ankrd17*	10982,79	-4,53	7,01E-06	5,50E-04	Spermatogonia
chr5	90460897	90476602	+	ENSMUST00000031314.9	*Alb*	1454,90	7,95	8,85E-06	6,65E-04	NA
chr5	92284010	92310024	-	ENSMUST00000031364.4	*Sdad1*	23465,88	-0,74	2,14E-14	9,32E-12	Multiple
chr5	92603051	92606579	+	ENSMUST00000050952.3	*Stbd1*	1084,25	0,83	3,30E-06	2,84E-04	Spermatogonia
chr5	92608253	92675125	-	ENSMUST00000060930.9	*Ccdc158*	7700,89	-0,84	3,59E-04	1,31E-02	Multiple
chr5	100553725	100572245	-	ENSMUST00000031264.11	*Plac8*	353,45	1,79	2,19E-03	4,71E-02	Multiple
chr5	108433244	108434448	-	ENSMUST00000049628.15	*Atp5k*	7625,69	0,62	1,86E-04	7,87E-03	Spermatogonia
chr5	110339812	110342955	-	ENSMUST00000112478.7	*P2rx2*	1724,13	-1,58	3,49E-12	1,00E-09	Elongated Spermatids
chr5	110339812	110343212	-	ENSMUST00000195985.4	*P2rx2*	999,77	1,57	1,23E-03	3,17E-02	Elongated Spermatids
chr5	110339813	110342976	-	ENSMUST00000058016.15	*P2rx2*	2317,73	1,87	1,56E-07	1,94E-05	Elongated Spermatids
chr5	112910482	113015514	-	ENSMUST00000065167.8	*Grk3*	13081,57	-0,76	3,31E-05	1,99E-03	NA
chr5	115341225	115343569	+	ENSMUST00000031508.4	*Triap1*	2032,06	0,70	5,63E-04	1,81E-02	Spermatogonia
chr5	115506676	115555987	+	ENSMUST00000067268.14	*Pxn*	1709,65	-3,78	1,81E-48	8,24E-45	Multiple
chr5	115559467	115563727	+	ENSMUST00000086519.11	*Rplp0*	218586,50	0,71	7,78E-04	2,29E-02	Multiple
chr5	123142957	123167410	+	ENSMUST00000056053.8	*Setd1b*	9314,84	-0,87	7,51E-04	2,23E-02	Pre-leptotene
chr5	124061530	124095798	-	ENSMUST00000031354.10	*Abcb9*	5905,45	-1,52	1,93E-03	4,34E-02	Multiple
chr5	124725085	124834308	+	ENSMUST00000058440.11	*Dnah10*	22164,71	-1,21	1,90E-04	8,01E-03	Multiple
chr5	134656039	134688598	-	ENSMUST00000015137.9	*Limk1*	2389,34	-0,91	2,04E-03	4,49E-02	Spermatogonia
chr5	134656040	134678248	-	ENSMUST00000111233.7	*Limk1*	1083,59	13,44	1,19E-27	1,84E-24	Spermatogonia
chr5	135248938	135251230	-	ENSMUST00000062572.2	*Fzd9*	1647,95	0,61	9,43E-08	1,24E-05	Spermatogonia
chr5	139252370	139267141	+	ENSMUST00000130326.7	*Get4*	5284,55	0,61	1,29E-03	3,27E-02	Spermatogonia
chr5	144255234	144264574	+	ENSMUST00000056578.6	*Bri3*	6026,86	0,89	7,52E-05	3,87E-03	Round Spermatids
chr5	147430161	147548502	+	ENSMUST00000031651.14	*Pan3*	5170,99	-1,04	2,96E-04	1,13E-02	Multiple
chr5	147430453	147548496	+	ENSMUST00000176600.7	*Pan3*	8160,35	-0,93	4,34E-05	2,48E-03	Multiple
chr6	5725812	6028039	+	ENSMUST00000115554.3	*Dync1i1*	1588,27	0,88	1,16E-04	5,44E-03	Pre-leptotene
chr6	8255936	8259173	-	ENSMUST00000012627.4	*Rpa3*	1127,20	0,88	1,23E-03	3,18E-02	Spermatogonia
chr6	8509600	8597548	+	ENSMUST00000064285.14	*Glcci1*	3814,39	3,69	1,46E-03	3,59E-02	Multiple
chr6	28423560	28426602	+	ENSMUST00000020717.11	*Arf5*	11754,92	0,92	6,99E-06	5,49E-04	Multiple
chr6	29396685	29426994	+	ENSMUST00000096084.11	*Ccdc136*	10372,99	-2,62	1,85E-23	2,13E-20	Multiple
chr6	29410105	29426988	+	ENSMUST00000202726.1	*Ccdc136*	257,46	-11,57	1,02E-08	1,62E-06	Multiple
chr6	35022066	35133694	-	ENSMUST00000114989.8	*Cnot4*	2004,54	-0,91	2,53E-04	9,95E-03	Multiple
chr6	39592574	39603382	+	ENSMUST00000135671.7	*Ndufb2*	2744,35	0,76	9,25E-05	4,56E-03	Multiple
chr6	40921784	40929740	-	ENSMUST00000136499.1	*1700074P13Rik*	2440,35	-0,76	1,98E-03	4,40E-02	Multiple
chr6	49073795	49086751	+	ENSMUST00000128616.5	*Malsu1*	1439,56	0,60	1,25E-03	3,21E-02	Spermatogonia
chr6	50562563	50566538	-	ENSMUST00000161401.1	*Cycs*	12191,07	0,74	1,33E-03	3,36E-02	Spermatogonia
chr6	54326883	54330201	+	ENSMUST00000059138.5	*Prr15*	2114,19	0,68	1,85E-05	1,24E-03	Round Spermatids
chr6	56081923	56369583	-	ENSMUST00000170774.7	*Pde1c*	7804,05	1,16	1,96E-05	1,29E-03	Multiple
chr6	71271677	71285319	+	ENSMUST00000204436.2	*Krcc1*	4689,86	-0,79	2,36E-13	8,76E-11	Multiple
chr6	73017742	73212663	-	ENSMUST00000204053.1	*Dnah6*	30917,59	-1,14	7,07E-07	7,42E-05	Multiple
chr6	83119044	83121559	-	ENSMUST00000113936.9	*Wbp1*	409,79	3,20	1,39E-04	6,28E-03	Multiple
chr6	83119044	83121559	-	ENSMUST00000032111.10	*Wbp1*	394,34	3,72	2,38E-13	8,80E-11	Multiple
chr6	85133687	85137756	-	ENSMUST00000174769.1	*Spr*	672,60	1,31	1,10E-03	2,93E-02	Spermatogonia
chr6	86365646	86370058	+	ENSMUST00000071492.8	*Fam136a*	4382,73	0,78	2,31E-03	4,89E-02	Spermatogonia
chr6	86736840	86765910	-	ENSMUST00000113675.7	*Anxa4*	540,08	9,99	6,35E-07	6,80E-05	Spermatogonia
chr6	86849532	86935512	+	ENSMUST00000204414.2	*Aak1*	619,01	-0,86	1,23E-07	1,58E-05	Spermatogonia
chr6	88193891	88207032	+	ENSMUST00000170089.7	*Gata2*	773,56	10,51	1,59E-12	4,81E-10	Round Spermatids
chr6	90462576	90475238	+	ENSMUST00000032174.11	*Klf15*	3125,23	1,20	1,36E-03	3,42E-02	Multiple
chr6	91516048	91522620	+	ENSMUST00000206947.1	*Lsm3*	7413,59	0,63	1,28E-05	9,14E-04	Spermatogonia
chr6	120509494	120531319	-	ENSMUST00000075303.6	*Hdhd5*	5836,51	1,91	1,84E-15	9,37E-13	NA

Supplementary Table 3. All differentially expressed genes (*continued*)

Chr	Start	End	Strand	Transcript ID	Gene Name	baseMean	log2FoldChange	pvalue	padj	Origin
chr6	124712336	124716950	+	ENSMUST00000004375.15	*Phb2*	5919,17	15,89	3,36E-38	1,06E-34	Spermatogonia
chr6	129533376	129541434	+	ENSMUST00000204956.1	*Gabarapl1*	23165,94	0,81	1,07E-07	1,40E-05	Elongated Spermatids
chr6	135137522	135168215	-	ENSMUST00000045855.8	*Hebp1*	1076,57	1,00	8,81E-05	4,36E-03	Pre-leptotene
chr6	135197977	135236242	+	ENSMUST00000111915.7	*Fam234b*	2844,57	-4,26	1,02E-04	4,93E-03	NA
chr6	136808244	136810074	+	ENSMUST00000074556.6	*H2afj*	4133,24	0,88	2,02E-03	4,46E-02	Multiple
chr6	136872435	136875823	-	ENSMUST00000032342.2	*Mgp*	4879,56	0,86	1,34E-03	3,37E-02	Multiple
chr6	138141370	138156559	+	ENSMUST00000120230.7	*Mgst1*	1925,73	7,47	4,69E-06	3,83E-04	Multiple
chr6	145397210	145434925	-	ENSMUST00000111708.8	*Lmntd1*	565,56	-12,71	1,04E-03	2,83E-02	Multiple
chr6	149141564	149150398	+	ENSMUST00000134306.7	*Etfbkmt*	417,85	-1,21	2,54E-25	3,41E-22	NA
chr7	3677789	3693523	-	ENSMUST00000038608.13	*Mboat7*	2882,47	0,60	8,26E-04	2,40E-02	Spermatogonia
chr7	4677707	4684661	-	ENSMUST00000206946.1	*Hspbp1*	2298,28	1,61	1,57E-03	3,76E-02	Multiple
chr7	16061814	16096334	+	ENSMUST00000209369.1	*Zfp541*	2331,18	-0,75	2,43E-12	7,06E-10	Multiple
chr7	19131693	19137812	+	ENSMUST00000165913.1	*Fbxo46*	3911,14	-0,62	1,73E-03	4,02E-02	Elongated Spermatids
chr7	19581035	19604486	-	ENSMUST00000086041.6	*Clasrp*	746,07	-7,42	2,19E-05	1,43E-03	Multiple
chr7	19724161	19749533	-	ENSMUST00000108450.4	*Nectin2*	7745,63	0,68	2,37E-04	9,46E-03	NA
chr7	24510483	24512455	-	ENSMUST00000234781.1	*Zfp428*	31756,10	-0,67	9,65E-04	2,68E-02	Multiple
chr7	24884651	24888950	+	ENSMUST00000153451.8	*Rps19*	1246,12	1,69	5,34E-10	1,05E-07	Spermatogonia
chr7	25516042	25539857	-	ENSMUST00000064862.12	*Ceacam2*	3147,14	-0,76	8,47E-07	8,66E-05	Multiple
chr7	27305136	27337692	-	ENSMUST00000038618.12	*Ltbp4*	1268,45	-8,50	1,93E-06	1,79E-04	Multiple
chr7	27486910	27490316	+	ENSMUST00000008528.7	*Sertad1*	2227,94	0,67	7,98E-04	2,34E-02	Multiple
chr7	28305516	28312072	-	ENSMUST00000081946.4	*Timm50*	1409,69	1,39	1,07E-03	2,88E-02	Spermatogonia
chr7	30616861	30626151	-	ENSMUST00000075738.5	*Cox6b1*	7416,04	0,71	6,68E-04	2,04E-02	Spermatogonia
chr7	34109543	34133268	-	ENSMUST00000038537.8	*Wtip*	2229,62	0,66	4,72E-04	1,60E-02	Spermatogonia
chr7	38216972	38228016	-	ENSMUST00000098513.5	*Plekhf1*	2409,17	0,72	1,48E-06	1,41E-04	Spermatogonia
chr7	44246722	44252319	+	ENSMUST00000055858.13	*2410002F23Rik*	1352,31	1,27	1,51E-10	3,20E-08	Spermatogonia
chr7	44246789	44252312	+	ENSMUST00000107950.8	*2410002F23Rik*	1132,57	-3,22	1,15E-04	5,42E-03	Spermatogonia
chr7	44261447	44261891	+	ENSMUST00000124863.3	*Gm15517*	3967,83	2,65	1,03E-04	4,96E-03	NA
chr7	44549616	44553949	-	ENSMUST00000073488.11	*Nr1h2*	1910,24	0,76	4,68E-05	2,64E-03	Multiple
chr7	44849224	44855421	+	ENSMUST00000054343.14	*Akt1s1*	997,53	-7,30	5,72E-06	4,57E-04	Multiple
chr7	44975989	44986492	-	ENSMUST00000207370.1	*Prmt1*	5246,45	0,75	2,79E-04	1,07E-02	Spermatogonia
chr7	45125558	45128761	-	ENSMUST00000150350.8	*Rpl13a*	44535,87	1,00	1,80E-06	1,68E-04	Spermatogonia
chr7	45282873	45288993	+	ENSMUST00000085364.3	*Slc6a21*	10722,84	-0,76	1,81E-03	4,14E-02	NA
chr7	45461867	45466831	-	ENSMUST00000210392.1	*Bax*	1523,87	0,64	1,69E-04	7,32E-03	Spermatogonia
chr7	45718157	45720828	+	ENSMUST00000209287.1	*Rpl18*	2918,05	0,66	1,40E-06	1,34E-04	Multiple
chr7	45825227	45830789	-	ENSMUST00000107723.8	*Grwd1*	1828,62	6,98	3,51E-08	5,05E-06	Multiple
chr7	49778346	49858265	+	ENSMUST00000032715.12	*Prmt3*	1604,99	0,84	2,91E-04	1,11E-02	Spermatogonia
chr7	52011685	52015703	-	ENSMUST00000185758.1	*1700015G11Rik*	1394,20	0,75	8,12E-04	2,37E-02	Elongated Spermatids
chr7	64392645	64412125	+	ENSMUST00000037205.10	*Mcee*	682,84	0,79	6,01E-04	1,89E-02	Spermatogonia
chr7	80454993	80535119	-	ENSMUST00000081314.10	*Blm*	1566,36	2,93	2,16E-08	3,24E-06	Multiple
chr7	82648614	82777852	+	ENSMUST00000179489.7	*Efl1*	4262,61	-0,76	1,33E-03	3,35E-02	NA
chr7	99837569	99858977	-	ENSMUST00000036274.7	*Spcs2*	4000,44	2,54	9,88E-04	2,73E-02	Multiple
chr7	101321113	101346308	+	ENSMUST00000032927.13	*Stard10*	2854,32	1,15	1,76E-03	4,07E-02	Multiple
chr7	101905904	101911903	+	ENSMUST00000033131.11	*Lamtor1*	8411,95	0,65	3,84E-04	1,38E-02	Spermatogonia
chr7	104218795	104235152	+	ENSMUST00000098180.9	*Trim6*	1448,37	1,11	5,17E-07	5,66E-05	Spermatogonia
chr7	107370790	107548656	+	ENSMUST00000073459.11	*Syt9*	1293,03	1,58	1,40E-03	3,49E-02	Spermatogonia
chr7	112023506	112110483	+	ENSMUST00000215510.1	*Usp47*	50376,24	-0,69	1,54E-08	2,36E-06	Multiple
chr7	112023565	112111661	+	ENSMUST00000210309.1	*Usp47*	3469,71	-0,72	9,12E-04	2,58E-02	Multiple
chr7	118843790	118855992	-	ENSMUST00000126792.8	*Knop1*	845,04	26,24	1,85E-11	4,47E-09	Multiple
chr7	120328684	120407687	+	ENSMUST00000076272.4	*Abca15*	7758,00	-0,74	3,91E-04	1,39E-02	Multiple
chr7	120740047	120805541	+	ENSMUST00000167213.8	*Vwa3a*	6330,75	-29,83	2,23E-14	9,67E-12	Elongated Spermatids
chr7	126672870	126676357	-	ENSMUST00000106372.10	*Sult1a1*	790,36	2,12	1,97E-03	4,39E-02	Pre-leptotene
chr7	126695436	126697058	+	ENSMUST00000130498.1	*Bola2*	3404,64	0,62	2,26E-04	9,10E-03	Spermatogonia
chr7	127256959	127260709	-	ENSMUST00000035276.4	*Dctpp1*	3011,02	0,82	4,18E-04	1,47E-02	Spermatogonia
chr7	127876796	127880188	+	ENSMUST00000131000.1	*Zfp646*	4421,19	-0,64	1,23E-03	3,18E-02	Multiple
chr7	133637543	133672971	+	ENSMUST00000051169.12	*Edrf1*	2142,40	-0,79	7,42E-04	2,21E-02	Spermatogonia
chr7	139600951	139683817	-	ENSMUST00000129990.8	*Cfap46*	14473,27	-0,72	4,40E-04	1,52E-02	Pre-leptotene
chr7	140954837	140955987	-	ENSMUST00000081649.9	*Ifitm2*	3084,59	0,66	2,05E-04	8,47E-03	Spermatogonia
chr7	141278338	141279131	-	ENSMUST00000167790.2	*Sct*	270,77	1,09	4,10E-04	1,44E-02	Spermatogonia
chr7	141447850	141451350	+	ENSMUST00000106003.1	*Rplp2*	235,90	0,65	5,43E-04	1,76E-02	Spermatogonia
chr7	142375917	142387857	-	ENSMUST00000066401.6	*Ctsd*	3314,51	28,15	5,73E-13	1,94E-10	Spermatogonia
chr8	3621602	3623372	+	ENSMUST00000207389.1	*Pet100*	1173,77	0,82	2,25E-04	9,07E-03	Multiple
chr8	21970596	21974041	+	ENSMUST00000017193.1	*Ccdc70*	7576,89	-3,52	5,48E-04	1,77E-02	Multiple
chr8	24677225	24725852	-	ENSMUST00000033958.14	*Adam3*	137079,13	-0,78	2,42E-06	2,17E-04	Multiple
chr8	34826460	34965690	-	ENSMUST00000033929.5	*Tnks*	47936,13	-0,69	4,91E-07	5,42E-05	Multiple
chr8	41239773	41332344	+	ENSMUST00000045218.8	*Pcm1*	3215,58	-0,90	3,08E-05	1,89E-03	Multiple
chr8	45934619	45944145	+	ENSMUST00000095326.9	*Ccdc110*	28323,77	-0,74	3,54E-06	3,00E-04	Multiple
chr8	46492832	46534480	+	ENSMUST00000110371.7	*Acsl1*	11534,97	-1,36	4,44E-05	2,53E-03	Elongated Spermatids
chr8	53511727	53523334	+	ENSMUST00000211424.1	*Aga*	656,84	6,78	4,00E-04	1,41E-02	Multiple
chr8	70504299	70506737	-	ENSMUST00000136913.1	*Rex1bd*	816,66	1,50	1,08E-03	2,90E-02	NA

Supplementary Table 3. All differentially expressed genes (*continued*)

Chr	Start	End	Strand	Transcript ID	Gene Name	baseMean	log2FoldChange	pvalue	padj	Origin
chr8	70527747	70535326	+	ENSMUST00000075491.13	*Fkbp8*	26999,95	0,64	1,00E-03	2,75E-02	Multiple
chr8	70703457	70717650	+	ENSMUST00000212436.1	*Iqcn*	12715,26	-1,14	2,18E-07	2,65E-05	NA
chr8	72723290	72739903	+	ENSMUST00000212095.1	*Sin3b*	23247,99	0,73	4,21E-06	3,48E-04	Multiple
chr8	83652677	83664694	+	ENSMUST00000019577.9	*Gipc1*	3986,19	0,59	5,35E-04	1,75E-02	Multiple
chr8	84689272	84695301	+	ENSMUST00000152301.8	*Trmt1*	637,83	-0,68	5,91E-07	6,40E-05	Pre-leptotene
chr8	84976910	84978718	-	ENSMUST00000064922.6	*Junb*	1614,84	0,65	6,61E-04	2,03E-02	Multiple
chr8	94172664	94173568	+	ENSMUST00000034214.7	*Mt2*	5102,12	3,18	2,24E-03	4,79E-02	Multiple
chr8	95055103	95078141	+	ENSMUST00000058479.6	*Drc7*	6724,80	3,26	1,91E-29	3,19E-26	Multiple
chr8	95743174	95761431	-	ENSMUST00000211973.1	*Cnot1*	1372,48	-1,06	2,32E-09	4,20E-07	Multiple
chr8	105768308	105827350	-	ENSMUST00000041400.5	*Ranbp10*	5363,53	-1,48	4,95E-13	1,71E-10	Multiple
chr8	106572966	106573461	-	ENSMUST00000073722.5	*Gm10073*	2066,86	0,84	1,12E-03	2,97E-02	Spermatogonia
chr8	107580381	107588464	-	ENSMUST00000044106.5	*Psmd7*	4912,01	0,66	1,47E-03	3,60E-02	Multiple
chr8	111643537	111670682	+	ENSMUST00000211926.1	*Zfp1*	659,34	0,96	1,52E-03	3,69E-02	Spermatogonia
chr8	122775505	122817880	+	ENSMUST00000015160.5	*Acsf3*	1654,67	-2,48	7,36E-07	7,68E-05	Multiple
chr8	122775578	122817880	+	ENSMUST00000212790.1	*Acsf3*	1071,06	1,42	8,09E-05	4,08E-03	Multiple
chr9	6262733	6269846	-	ENSMUST00000051706.5	*Ddi1*	3909,84	-5,06	2,54E-32	5,66E-29	Multiple
chr9	6928503	7184446	-	ENSMUST00000140466.7	*Dync2h1*	42314,11	-0,81	4,26E-10	8,45E-08	Multiple
chr9	8076461	8134294	-	ENSMUST00000037397.7	*Cep126*	14444,74	-0,61	1,49E-04	6,63E-03	Multiple
chr9	15306264	15311798	+	ENSMUST00000164079.8	*Taf1d*	349,11	-2,03	1,50E-03	3,64E-02	Spermatogonia
chr9	20652095	20666584	+	ENSMUST00000034689.7	*Pin1*	13006,82	0,66	2,29E-04	9,19E-03	Multiple
chr9	21104070	21131232	-	ENSMUST00000216874.1	*Tyk2*	2543,35	10,57	3,25E-07	3,76E-05	Multiple
chr9	21592722	21595970	+	ENSMUST00000062125.10	*Timm29*	2716,86	5,68	1,84E-03	4,20E-02	NA
chr9	35124408	35176061	-	ENSMUST00000034539.11	*Dcps*	4563,90	0,85	1,83E-06	1,71E-04	Spermatogonia
chr9	50605240	50617464	-	ENSMUST00000131351.7	*Nkapd1*	1433,76	-3,50	2,19E-03	4,71E-02	NA
chr9	54764748	54773110	+	ENSMUST00000034830.8	*Crabp1*	1790,60	0,76	5,57E-04	1,79E-02	Spermatogonia
chr9	55208925	55224433	+	ENSMUST00000034859.14	*Fbxo22*	1759,41	0,91	2,45E-05	1,57E-03	Spermatogonia
chr9	57537563	57543184	+	ENSMUST00000114200.9	*Fam219b*	1424,88	0,76	6,66E-05	3,50E-03	Spermatogonia
chr9	57708540	57752499	+	ENSMUST00000043990.13	*Edc3*	2935,53	3,26	8,06E-04	2,36E-02	Spermatogonia
chr9	59750896	59928866	+	ENSMUST00000128341.1	*Myo9a*	33268,32	-0,82	5,23E-11	1,18E-08	Multiple
chr9	61913284	61914542	-	ENSMUST00000008036.8	*Rplp1*	71425,53	0,74	6,98E-04	2,10E-02	Multiple
chr9	63421620	63602448	-	ENSMUST00000163982.7	*Iqch*	9214,50	-0,82	1,18E-07	1,53E-05	Multiple
chr9	63421662	63602483	-	ENSMUST00000080527.11	*Iqch*	4236,23	-0,89	1,52E-07	1,91E-05	Multiple
chr9	65909088	66040440	+	ENSMUST00000206594.1	*Csnk1g1*	1199,24	1,46	1,72E-03	4,01E-02	Multiple
chr9	66946091	66949514	+	ENSMUST00000127896.7	*Rps27l*	1721,92	0,88	4,82E-04	1,62E-02	Multiple
chr9	69453620	69491795	+	ENSMUST00000034756.14	*Anxa2*	7963,95	1,21	2,79E-09	4,93E-07	Multiple
chr9	75625741	75637771	+	ENSMUST00000034702.5	*Lysmd2*	833,27	0,64	1,08E-04	5,16E-03	Spermatogonia
chr9	79755361	79759878	-	ENSMUST00000034881.7	*Cox7a2*	331,85	3,68	7,01E-04	2,11E-02	Multiple
chr9	97349562	97369958	-	ENSMUST00000035026.4	*Trim42*	8863,64	-0,82	2,06E-03	4,53E-02	Elongated Spermatids
chr9	100495003	100506856	-	ENSMUST00000112874.3	*Nck1*	856,93	1,33	7,11E-05	3,71E-03	Multiple
chr9	107327084	107338350	-	ENSMUST00000035196.13	*Hemk1*	3825,07	0,72	5,08E-04	1,69E-02	Multiple
chr9	107614125	107635367	-	ENSMUST00000055704.11	*Gnai2*	22608,06	1,08	1,96E-03	4,38E-02	Multiple
chr9	107787813	107791928	-	ENSMUST00000194436.1	*Rbm6*	280,97	11,49	3,51E-05	2,09E-03	Multiple
chr9	108783796	108797989	+	ENSMUST00000192307.5	*Ip6k2*	506,26	0,76	4,16E-04	1,46E-02	Multiple
chr9	109816627	109826628	+	ENSMUST00000118732.3	*Spink8*	281,13	2,02	4,72E-05	2,66E-03	Multiple
chr9	110062591	110083170	+	ENSMUST00000163979.6	*Map4*	4816,47	-4,44	1,87E-04	7,89E-03	Multiple
chr9	110131980	110240178	+	ENSMUST00000088716.11	*Smarcc1*	2681,48	5,36	6,65E-07	7,06E-05	Spermatogonia
chr9	119894895	119921958	+	ENSMUST00000217472.1	*Wdr48*	1185,59	3,60	2,17E-03	4,68E-02	Multiple
chr9	119971165	119977335	-	ENSMUST00000215916.1	*Csrnp1*	1564,43	0,77	7,30E-06	5,69E-04	Elongated Spermatids
chr9	120571444	120574654	+	ENSMUST00000165532.2	*Rpl14*	15591,03	0,89	3,53E-05	2,09E-03	Spermatogonia
chr9	123150946	123157432	+	ENSMUST00000026890.5	*Clec3b*	3399,99	1,06	5,30E-06	4,29E-04	Multiple
chr9	123828512	123851881	-	ENSMUST00000184082.2	*Fyco1*	10695,60	-0,82	1,29E-07	1,65E-05	Multiple
chr9	124422623	124423583	-	ENSMUST00000188509.1	*Ppp2r3d*	658,47	0,76	5,60E-18	3,64E-15	Round Spermatids
chr10	5799160	5805600	-	ENSMUST00000019907.7	*Fbxo5*	843,95	0,63	1,66E-03	3,91E-02	Spermatogonia
chr10	13524142	13553120	-	ENSMUST00000105539.1	*Pex3*	4802,39	-6,30	2,77E-07	3,28E-05	Multiple
chr10	20952547	21080429	+	ENSMUST00000105525.11	*Ahi1*	34547,54	-0,59	4,45E-05	2,53E-03	Multiple
chr10	23894688	23905343	+	ENSMUST00000041416.7	*Vnn1*	1240,20	1,39	1,21E-05	8,70E-04	Multiple
chr10	58255530	58305242	+	ENSMUST00000162041.7	*Gcc2*	4017,48	-3,74	2,80E-53	2,09E-49	Multiple
chr10	62315476	62379852	-	ENSMUST00000133429.7	*Hk1*	654,11	-0,69	1,83E-03	4,19E-02	Multiple
chr10	67127258	67256318	+	ENSMUST00000174408.7	*Jmjd1c*	5750,56	-4,92	1,18E-05	8,57E-04	Multiple
chr10	75783813	75798584	-	ENSMUST00000001713.9	*Gstt1*	580,73	1,23	1,32E-05	9,33E-04	Spermatogonia
chr10	77606225	77616643	+	ENSMUST00000141228.8	*Sumo3*	368,23	11,88	1,80E-03	4,13E-02	Multiple
chr10	77606571	77617422	+	ENSMUST00000099538.5	*Sumo3*	2725,98	-4,34	2,28E-07	2,75E-05	Multiple
chr10	79669369	79676127	+	ENSMUST00000020552.6	*Tpgs1*	7274,81	0,62	2,20E-05	1,43E-03	Multiple
chr10	79854668	79864359	+	ENSMUST00000095457.10	*Ptbp1*	11326,33	-1,01	3,97E-05	2,31E-03	Multiple
chr10	80318254	80320537	-	ENSMUST00000020341.8	*2310011J03Rik*	4176,62	0,74	1,35E-03	3,39E-02	Multiple
chr10	80329953	80336441	+	ENSMUST00000105358.7	*Reep6*	696,77	2,75	9,48E-04	2,65E-02	Elongated Spermatids
chr10	80796099	80798626	-	ENSMUST00000036805.6	*Plekhj1*	2511,70	0,62	5,70E-06	4,56E-04	Multiple
chr10	80899450	80900969	-	ENSMUST00000020440.6	*Timm13*	5208,93	0,70	2,34E-03	4,94E-02	Multiple
chr10	81384428	81388352	+	ENSMUST00000072751.12	*Dohh*	4625,78	1,34	9,33E-04	2,62E-02	Spermatogonia

Supplementary Table 3. All differentially expressed genes (*continued*)

Chr	Start	End	Strand	Transcript ID	Gene Name	baseMean	log2FoldChange	pvalue	padj	Origin
chr10	82728349	82741395	+	ENSMUST00000160681.1	*Hcfc2*	1335,21	4,50	3,73E-05	2,20E-03	Multiple
chr10	84838148	84906538	+	ENSMUST00000095388.4	*Rfx4*	2471,14	-7,98	1,36E-04	6,16E-03	Multiple
chr10	89638721	89686285	-	ENSMUST00000174252.7	*Scyl2*	1600,63	4,30	4,98E-04	1,66E-02	Multiple
chr10	92775619	93081618	-	ENSMUST00000168110.7	*Cfap54*	13069,26	-0,62	3,08E-05	1,89E-03	Multiple
chr10	92776179	93081596	-	ENSMUST00000212902.1	*Cfap54*	4175,27	-0,94	1,87E-08	2,83E-06	Multiple
chr10	116111667	116113702	-	ENSMUST00000220165.1	*4933416C03Rik*	2045,05	8,27	1,39E-06	1,34E-04	Multiple
chr10	128490861	128493857	-	ENSMUST00000218127.1	*Myl6*	4423,05	0,82	1,27E-03	3,24E-02	Multiple
chr10	128919914	128923524	-	ENSMUST00000026405.9	*Bloc1s1*	6120,55	0,71	1,07E-03	2,88E-02	Multiple
chr11	6389364	6406158	+	ENSMUST00000012612.10	*Zmiz2*	4011,50	-0,77	1,20E-08	1,87E-06	Multiple
chr11	6389471	6406158	+	ENSMUST00000109786.7	*Zmiz2*	3492,88	-0,91	1,29E-05	9,19E-04	Multiple
chr11	6620997	6626067	-	ENSMUST00000136682.7	*Tbrg4*	323,52	1,42	6,69E-05	3,51E-03	Spermatogonia
chr11	20543334	20653021	+	ENSMUST00000093292.10	*Sertad2*	10331,30	-1,00	3,24E-06	2,79E-04	Multiple
chr11	29547950	29578367	+	ENSMUST00000020753.3	*Clhc1*	1010,17	2,96	9,53E-04	2,66E-02	Spermatocytes
chr11	46404730	46407982	+	ENSMUST00000063166.5	*Fam71b*	13820,14	-0,84	2,36E-06	2,14E-04	Elongated Spermatids
chr11	50940711	50943765	-	ENSMUST00000109123.1	*4933414I15Rik*	4518,07	1,62	1,07E-19	8,54E-17	Multiple
chr11	59662507	59776223	+	ENSMUST00000066330.14	*Mprip*	34840,23	-1,04	3,62E-06	3,07E-04	Multiple
chr11	62029253	62221129	+	ENSMUST00000201364.3	*Specc1*	2194,30	-3,16	2,37E-04	9,44E-03	Multiple
chr11	69810965	69815298	-	ENSMUST00000210714.1	*Gm39566*	48927,45	-1,10	8,14E-05	4,10E-03	NA
chr11	69916720	69921386	-	ENSMUST00000043419.9	*Eif5a*	1320,54	3,62	1,22E-49	6,67E-46	Multiple
chr11	77090185	77151925	-	ENSMUST00000130901.1	*Efcab5*	5812,26	-0,86	8,31E-07	8,52E-05	Multiple
chr11	77216287	77460220	+	ENSMUST00000037912.11	*Ssh2*	90491,39	-0,79	6,54E-08	8,89E-06	Multiple
chr11	78343482	78349164	+	ENSMUST00000002127.13	*Unc119*	4730,60	0,86	3,66E-05	2,16E-03	Spermatogonia
chr11	78522850	78536332	-	ENSMUST00000108277.2	*Tnfaip1*	1303,80	-1,37	1,25E-03	3,20E-02	Spermatogonia
chr11	82803822	82871210	-	ENSMUST00000108173.9	*Rffl*	1203,93	2,53	7,38E-24	8,64E-21	Multiple
chr11	87127077	87220683	+	ENSMUST00000041282.12	*Trim37*	130822,37	-0,67	2,32E-04	9,28E-03	Multiple
chr11	89037582	89060748	-	ENSMUST00000000285.8	*Dgke*	1717,64	-1,57	6,06E-05	3,23E-03	Multiple
chr11	95666957	95680773	+	ENSMUST00000125172.7	*Phb*	15587,85	0,67	1,56E-04	6,89E-03	Spermatogonia
chr11	97030134	97032276	+	ENSMUST00000153482.1	*Scrn2*	635,73	0,66	1,18E-03	3,08E-02	Spermatogonia
chr11	97206933	97280432	-	ENSMUST00000165216.7	*Npepps*	904,62	-13,39	6,07E-04	1,90E-02	Multiple
chr11	100408456	100414836	-	ENSMUST00000066489.12	*P3h4*	4146,16	0,73	1,68E-04	7,31E-03	Spermatogonia
chr11	101197389	101226426	-	ENSMUST00000100417.2	*Ezh1*	1486,36	-2,02	2,29E-35	5,68E-32	Spermatogonia
chr11	101468175	101471853	+	ENSMUST00000001347.6	*Rnd2*	3409,33	0,80	8,38E-04	2,42E-02	Spermatogonia
chr11	102189620	102194081	+	ENSMUST00000070334.9	*G6pc3*	1177,36	13,56	5,20E-04	1,71E-02	Multiple
chr11	102393403	102402555	+	ENSMUST00000006750.7	*Rundc3a*	3324,11	4,37	7,90E-05	4,01E-03	Elongated Spermatids
chr11	103451955	103504597	-	ENSMUST00000153273.1	*Lrrc37a*	11204,11	-1,43	2,64E-05	1,67E-03	Round Spermatids
chr11	104685707	105117394	+	ENSMUST00000212287.1	*Gm11639*	23364,79	-1,18	4,52E-05	2,57E-03	Round Spermatids
chr11	105396695	105408707	-	ENSMUST00000151305.1	*March10*	305,92	-0,95	1,55E-11	3,84E-09	NA
chr11	105933702	105944412	-	ENSMUST00000019734.10	*Cyb561*	1846,18	-2,73	5,45E-04	1,77E-02	Spermatogonia
chr11	109362831	109401369	+	ENSMUST00000020930.13	*Gna13*	3387,56	-3,37	1,27E-04	5,84E-03	Multiple
chr11	110100749	110168196	-	ENSMUST00000044850.3	*Abca9*	3802,98	-0,65	2,04E-03	4,49E-02	Elongated Spermatids
chr11	113776374	114066218	-	ENSMUST00000041627.13	*Sdk2*	4722,76	-0,69	2,11E-03	4,60E-02	Elongated Spermatids
chr11	115603925	115608036	+	ENSMUST00000058109.8	*Mrps7*	6504,18	0,60	1,24E-03	3,18E-02	Spermatogonia
chr11	115644045	115699534	-	ENSMUST00000106497.7	*Grb2*	1623,68	2,71	4,96E-04	1,66E-02	Spermatogonia
chr11	116030322	116061214	+	ENSMUST00000021116.11	*Unk*	1326,55	2,14	2,14E-03	4,64E-02	Multiple
chr11	116288001	116306734	-	ENSMUST00000106411.9	*Exoc7*	1987,74	-4,86	1,72E-04	7,41E-03	Multiple
chr11	117115172	117159265	+	ENSMUST00000021177.14	*Sec14l1*	18872,32	-1,44	3,30E-12	9,34E-10	Multiple
chr11	117115238	117157559	+	ENSMUST00000103026.9	*Sec14l1*	8802,60	-1,40	1,49E-08	2,29E-06	Multiple
chr11	117332345	117362324	+	ENSMUST00000106349.1	*sept-09*	2441,38	12,82	8,16E-05	4,10E-03	NA
chr11	117815526	117826092	-	ENSMUST00000026661.3	*Tk1*	583,41	0,75	1,66E-04	7,22E-03	Spermatogonia
chr11	118023076	118130634	-	ENSMUST00000132685.8	*Dnah17*	139310,66	-0,99	8,29E-05	4,15E-03	Multiple
chr11	118332360	118342500	-	ENSMUST00000103024.3	*Cep295nl*	4668,20	-0,59	1,86E-04	7,87E-03	NA
chr11	119288363	119300089	-	ENSMUST00000026667.14	*Eif4a3*	3435,39	0,60	4,08E-12	1,12E-09	Multiple
chr11	120484613	120489065	+	ENSMUST00000043627.7	*Mrpl12*	15292,80	0,99	6,06E-11	1,36E-08	Multiple
chr11	121146143	121148319	+	ENSMUST00000039088.8	*Tex19,1*	4537,76	0,60	7,58E-05	3,89E-03	NA
chr11	121177591	121204711	-	ENSMUST00000026169.6	*Ogfod3*	1143,94	0,63	1,56E-04	6,87E-03	Spermatogonia
chr12	8674134	8751454	+	ENSMUST00000111123.8	*Pum2*	1527,98	26,95	5,22E-12	1,40E-09	Multiple
chr12	10390772	10395562	+	ENSMUST00000020947.6	*Rdh14*	4257,13	0,69	1,56E-04	6,87E-03	Multiple
chr12	21286358	21315056	+	ENSMUST00000067284.9	*Cpsf3*	5536,56	3,04	9,12E-22	9,58E-19	Multiple
chr12	21286399	21308236	+	ENSMUST00000222968.1	*Cpsf3*	861,13	3,74	8,51E-06	6,47E-04	Multiple
chr12	28751803	28869865	+	ENSMUST00000221877.1	*Eipr1*	2997,94	0,94	1,43E-03	3,54E-02	NA
chr12	35047186	35147479	+	ENSMUST00000048519.16	*Snx13*	3943,62	-0,65	1,51E-11	3,76E-09	Multiple
chr12	65062424	65074007	-	ENSMUST00000021332.9	*Fkbp3*	3127,17	1,06	4,15E-08	5,84E-06	Spermatogonia
chr12	69310522	69357165	-	ENSMUST00000021368.9	*Nemf*	7465,19	-0,96	4,88E-07	5,41E-05	Multiple
chr12	76022300	76027815	+	ENSMUST00000148825.1	*Syne2*	448,57	-1,07	6,56E-09	1,09E-06	Multiple
chr12	80335848	80436601	-	ENSMUST00000054145.7	*Dcaf5*	6051,03	3,00	3,90E-09	6,79E-07	Elongated Spermatids
chr12	81376991	81379464	-	ENSMUST00000062182.7	*Gm4787*	28956,44	-0,61	1,15E-08	1,80E-06	Elongated Spermatids
chr12	83632252	83683123	+	ENSMUST00000048155.15	*Rbm25*	8770,94	-2,56	1,47E-06	1,41E-04	Multiple
chr12	85824985	85843787	+	ENSMUST00000142411.8	*Ttll5*	304,63	-0,69	1,56E-03	3,75E-02	Multiple
chr12	87200543	87213541	+	ENSMUST00000182869.1	*Samd15*	2077,43	-0,63	5,04E-04	1,68E-02	Multiple

Supplementary Table 3. All differentially expressed genes (*continued*)

Chr	Start	End	Strand	Transcript ID	Gene Name	baseMean	log2FoldChange	pvalue	padj	Origin
chr12	103434259	103440239	+	ENSMUST00000085065.11	*Ifi27*	2081,37	1,51	3,94E-04	1,40E-02	Multiple
chr12	104112780	104121808	+	ENSMUST00000185595.6	*Serpina3a*	1418,85	0,63	1,03E-04	4,98E-03	Pre-leptotene
chr12	104115970	104121518	+	ENSMUST00000021496.7	*Serpina3a*	11491,88	0,65	2,88E-04	1,10E-02	Pre-leptotene
chr12	110485739	110583062	+	ENSMUST00000109832.2	*Ppp2r5c*	45847,80	-0,64	1,05E-03	2,85E-02	Multiple
chr12	110601452	110666945	+	ENSMUST00000018851.13	*Dync1h1*	186999,63	-0,86	5,00E-06	4,07E-04	Multiple
chr12	112644679	112649145	+	ENSMUST00000021728.11	*Siva1*	2787,26	0,94	4,04E-04	1,42E-02	Spermatogonia
chr12	113014508	113072138	+	ENSMUST00000223502.1	*Pacs2*	9172,72	-9,60	1,41E-04	6,34E-03	Multiple
chr13	3538130	3565820	+	ENSMUST00000059515.7	*Gdi2*	2240,37	27,60	1,61E-12	4,84E-10	Multiple
chr13	21810465	21810944	+	ENSMUST00000091745.5	*Hist1h2ao*	1691,56	0,98	2,10E-04	8,64E-03	NA
chr13	21981520	21984479	+	ENSMUST00000017126.5	*Pom121l2*	54486,93	-0,68	1,44E-03	3,55E-02	Multiple
chr13	28257528	28258134	+	ENSMUST00000223428.1	*Gm11361*	12472,03	0,68	1,02E-04	4,94E-03	Spermatogonia
chr13	34162964	34178168	+	ENSMUST00000124996.7	*Psmg4*	980,77	1,50	5,05E-04	1,68E-02	Spermatogonia
chr13	48801750	48871119	-	ENSMUST00000035540.8	*Phf2*	24879,22	-0,60	3,33E-06	2,85E-04	Multiple
chr13	49653078	49678738	+	ENSMUST00000222197.1	*Nol8*	15461,51	3,47	2,20E-03	4,73E-02	Multiple
chr13	51846678	51848468	+	ENSMUST00000021903.2	*Gadd45g*	656,96	2,86	4,04E-06	3,36E-04	Spermatogonia
chr13	54503779	54551290	+	ENSMUST00000121401.7	*Simc1*	3061,05	2,13	3,19E-06	2,75E-04	Multiple
chr13	58379817	58385225	-	ENSMUST00000022032.6	*2210016F16Rik*	4443,71	0,67	1,22E-03	3,15E-02	Spermatogonia
chr13	59700083	59706181	-	ENSMUST00000066510.7	*Spata31d1a*	19750,79	-0,81	3,48E-05	2,07E-03	Elongated Spermatids
chr13	59712284	59719295	+	ENSMUST00000165133.2	*Spata31d1b*	38548,67	-0,74	4,21E-04	1,47E-02	Elongated Spermatids
chr13	64363214	64370306	-	ENSMUST00000021933.7	*Ctsl*	71059,22	0,63	2,79E-05	1,74E-03	Multiple
chr13	69612244	69635780	+	ENSMUST00000022087.6	*Nsun2*	6042,97	-1,43	2,13E-03	4,62E-02	Multiple
chr13	69802417	69813747	+	ENSMUST00000222387.1	*Med10*	1999,60	-0,65	2,32E-03	4,91E-02	Multiple
chr13	86044816	86046904	-	ENSMUST00000131011.1	*Cox7c*	17621,10	0,62	2,17E-03	4,68E-02	Multiple
chr13	93304799	93403681	+	ENSMUST00000080127.11	*Homer1*	1478,25	1,29	2,83E-04	1,09E-02	Spermatogonia
chr13	97176331	97198357	-	ENSMUST00000022169.9	*Hexb*	3464,09	0,66	2,29E-04	9,19E-03	Spermatogonia
chr13	97760072	97760632	-	ENSMUST00000074072.4	*Gm10260*	30540,59	0,61	4,74E-04	1,61E-02	Multiple
chr13	100497763	100552718	-	ENSMUST00000022140.11	*Ocln*	1180,49	-1,84	6,53E-17	3,82E-14	Spermatogonia
chr14	8080367	8091834	+	ENSMUST00000023924.3	*Rpp14*	1546,09	0,66	1,50E-03	3,64E-02	Spermatogonia
chr14	18268960	18270970	-	ENSMUST00000100799.8	*Rpl15*	1035,04	1,77	6,14E-07	6,60E-05	Spermatogonia
chr14	18268960	18271029	-	ENSMUST00000079419.11	*Rpl15*	3066,01	2,00	6,10E-08	8,37E-06	Spermatogonia
chr14	18269000	18270626	-	ENSMUST00000112598.8	*Rpl15*	1864,36	3,49	7,19E-08	9,65E-06	Spermatogonia
chr14	20389304	20393634	-	ENSMUST00000061444.4	*Mrps16*	3563,99	0,68	1,38E-04	6,24E-03	Spermatogonia
chr14	40906445	40966807	-	ENSMUST00000047652.5	*Tspan14*	1008,76	-1,59	4,70E-04	1,60E-02	Spermatogonia
chr14	42792568	42797987	-	ENSMUST00000100695.3	*Gm10377*	1988,31	-28,08	6,61E-13	2,21E-10	NA
chr14	44163169	44171371	-	ENSMUST00000100688.2	*4930503E14Rik*	624,22	2,07	1,05E-03	2,86E-02	Spermatocytes
chr14	54259232	54269164	+	ENSMUST00000041197.11	*Abhd4*	5889,02	1,27	8,19E-04	2,39E-02	Multiple
chr14	54426909	54429756	+	ENSMUST00000010550.11	*Mrpl52*	4035,90	0,64	1,20E-05	8,65E-04	Multiple
chr14	54426930	54429749	+	ENSMUST00000199195.2	*Mrpl52*	215,42	0,90	3,45E-05	2,06E-03	Multiple
chr14	55680410	55681804	-	ENSMUST00000227873.1	*Tinf2*	319,52	0,90	2,99E-05	1,84E-03	Spermatogonia
chr14	57689662	57746123	-	ENSMUST00000022531.13	*Lats2*	11660,91	-1,81	2,82E-21	2,81E-18	Multiple
chr14	60205282	60251479	-	ENSMUST00000225311.1	*Gm49336*	5933,95	-0,67	1,49E-03	3,63E-02	NA
chr14	69717000	69732534	-	ENSMUST00000036381.9	*Chmp7*	11359,87	0,63	1,33E-03	3,35E-02	Spermatogonia
chr14	70164362	70167839	-	ENSMUST00000129174.7	*Pdlim2*	635,61	1,35	1,08E-03	2,90E-02	Elongated Spermatids
chr14	78495776	78522650	-	ENSMUST00000022593.6	*Akap11*	4365,87	-1,11	1,95E-05	1,29E-03	Multiple
chr14	120911224	120947999	+	ENSMUST00000032898.8	*Ipo5*	21151,65	-1,70	2,96E-05	1,83E-03	Multiple
chr15	25940826	25973668	+	ENSMUST00000226438.1	*Retreg1*	1576,29	9,94	1,58E-06	1,50E-04	NA
chr15	34484021	34495255	-	ENSMUST00000022946.5	*Rida*	573,24	0,78	1,33E-03	3,35E-02	NA
chr15	37968293	38078425	-	ENSMUST00000226414.1	*Ubr5*	25640,87	-0,85	3,85E-04	1,38E-02	Multiple
chr15	52040107	52045722	+	ENSMUST00000090025.4	*Aard*	53851,74	0,77	2,81E-04	1,08E-02	Multiple
chr15	57912199	57970061	+	ENSMUST00000022992.12	*Tbc1d31*	1449,29	1,01	1,66E-05	1,14E-03	Multiple
chr15	58022259	58034315	-	ENSMUST00000100655.4	*9130401M01Rik*	2188,13	0,70	5,92E-04	1,87E-02	Spermatogonia
chr15	76533213	76534931	-	ENSMUST00000230604.1	*Tmem249*	997,85	-0,89	8,60E-06	6,51E-04	NA
chr15	76899910	76901305	+	ENSMUST00000068407.5	*Commd5*	2023,98	0,77	2,09E-10	4,34E-08	Multiple
chr15	78926725	78930465	+	ENSMUST00000089377.5	*Lgals1*	5938,90	0,71	6,52E-04	2,01E-02	Spermatogonia
chr15	79724070	79742534	-	ENSMUST00000089311.10	*Sun2*	1438,52	1,39	9,52E-04	2,66E-02	Spermatogonia
chr15	82059151	82066540	+	ENSMUST00000100396.3	*4930407I10Rik*	50278,50	-0,99	1,55E-04	6,82E-03	Elongated Spermatids
chr15	84923449	84940007	+	ENSMUST00000230411.1	*Nup50*	2475,94	-0,73	2,36E-03	4,96E-02	Multiple
chr15	85814670	85821734	-	ENSMUST00000064370.5	*Pkdrej*	17354,60	-0,80	1,04E-04	5,01E-03	Multiple
chr15	88913898	88954410	-	ENSMUST00000109371.7	*Ttll8*	7104,18	-0,97	2,20E-04	8,92E-03	Multiple
chr15	89155549	89170688	-	ENSMUST00000060808.9	*Plxnb2*	5007,27	-2,87	1,13E-04	5,31E-03	Pre-leptotene
chr15	97964229	97970274	+	ENSMUST00000229428.1	*Tmem106c*	4768,65	0,67	2,77E-04	1,07E-02	Multiple
chr15	98949837	98953703	-	ENSMUST00000097014.6	*Tuba1a*	16464,80	1,73	1,74E-04	7,49E-03	Multiple
chr15	99725618	99728136	+	ENSMUST00000023761.2	*Cox14*	4563,95	0,76	1,49E-03	3,63E-02	Spermatogonia
chr15	100761869	100775084	+	ENSMUST00000161564.1	*Slc4a8*	213,82	0,86	1,23E-03	3,17E-02	Multiple
chr15	101266846	101274795	+	ENSMUST00000023779.7	*Nr4a1*	7629,67	-0,69	1,58E-03	3,78E-02	Multiple
chr15	101284272	101290945	+	ENSMUST00000048393.7	*Atg101*	3250,74	0,64	2,14E-03	4,63E-02	Spermatogonia
chr15	101996698	102004482	-	ENSMUST00000023952.9	*Krt8*	1278,09	1,51	1,71E-04	7,40E-03	Multiple
chr15	102144362	102149511	+	ENSMUST00000023807.6	*Igfbp6*	15188,59	0,76	7,67E-04	2,27E-02	Spermatogonia
chr15	102472737	102500061	+	ENSMUST00000078404.14	*Pcbp2*	7346,50	2,29	5,71E-05	3,09E-03	Multiple

Supplementary Table 3. All differentially expressed genes (*continued*)

Chr	Start	End	Strand	Transcript ID	Gene Name	baseMean	log2FoldChange	pvalue	padj	Origin
chr16	4756903	4766245	+	ENSMUST00000118885.7	*Hmox2*	3661,10	2,47	4,41E-04	1,52E-02	Multiple
chr16	4756994	4765090	+	ENSMUST00000121529.1	*Hmox2*	1430,23	13,84	2,75E-10	5,59E-08	Multiple
chr16	17208603	17213982	+	ENSMUST00000169803.4	*Rimbp3*	154653,73	-0,65	1,01E-04	4,89E-03	Multiple
chr16	18780453	18811626	-	ENSMUST00000096990.9	*Cdc45*	4635,51	0,72	1,55E-04	6,83E-03	Spermatogonia
chr16	18812294	18835261	+	ENSMUST00000005394.12	*Ufd1*	732,08	7,91	5,01E-05	2,79E-03	NA
chr16	22009484	22049269	+	ENSMUST00000023561.7	*Senp2*	9776,32	-1,24	7,79E-04	2,29E-02	Multiple
chr16	22813220	22857667	-	ENSMUST00000232075.1	*Tbccd1*	1173,31	1,65	1,91E-04	8,06E-03	Multiple
chr16	33380836	33504363	+	ENSMUST00000165418.8	*Zfp148*	5086,46	-1,12	2,28E-03	4,84E-02	Spermatogonia
chr16	35397312	35490873	-	ENSMUST00000023550.8	*Pdia5*	9122,56	0,63	2,34E-07	2,81E-05	Spermatogonia
chr16	36875168	36933085	+	ENSMUST00000114812.8	*Golgb1*	15366,10	-0,65	1,24E-03	3,19E-02	Multiple
chr16	38297754	38342143	-	ENSMUST00000023501.15	*Maats1*	14961,74	-0,65	1,14E-03	3,02E-02	Multiple
chr16	38396117	38433145	-	ENSMUST00000002926.7	*Pla1a*	1306,40	0,78	1,28E-03	3,27E-02	Elongated Spermatids
chr16	44401421	44482094	+	ENSMUST00000120049.7	*Cfap44*	22282,59	-0,88	2,20E-04	8,90E-03	Multiple
chr16	50191844	50432390	-	ENSMUST00000138166.7	*Bbx*	4951,86	-10,40	2,24E-08	3,33E-06	Multiple
chr16	50195780	50432340	-	ENSMUST00000066037.12	*Bbx*	5458,82	-4,91	1,83E-06	1,71E-04	Multiple
chr16	64477810	64602408	-	ENSMUST00000209382.2	*Csnka2ip*	3980,19	-0,92	7,30E-05	3,78E-03	Elongated Spermatids
chr16	77013706	77116780	+	ENSMUST00000023580.7	*Usp25*	31522,43	-0,72	1,17E-03	3,08E-02	Multiple
chr16	94364447	94370694	-	ENSMUST00000113910.7	*Pigp*	745,75	0,84	9,17E-04	2,59E-02	Spermatogonia
chr16	96158407	96192250	-	ENSMUST00000113804.7	*Lca5l*	6988,41	-1,89	3,23E-96	5,29E-92	Multiple
chr17	8311102	8327442	+	ENSMUST00000154553.1	*Sft2d1*	1581,39	1,20	1,56E-03	3,74E-02	Multiple
chr17	13716436	13761601	-	ENSMUST00000127032.7	*Tcte2*	2026,06	-1,97	4,34E-05	2,48E-03	Multiple
chr17	13716436	13761825	-	ENSMUST00000148430.1	*Tcte2*	11850,47	-1,20	2,48E-16	1,42E-13	Multiple
chr17	23776916	23786081	-	ENSMUST00000024704.9	*Flywch2*	1355,00	0,63	6,84E-20	5,66E-17	Multiple
chr17	24264279	24347252	-	ENSMUST00000039324.6	*Abca17*	19535,95	-0,62	4,45E-04	1,53E-02	Pre-leptotene
chr17	24414675	24425895	+	ENSMUST00000163717.1	*Rnps1*	1842,02	0,72	3,31E-04	1,23E-02	Spermatogonia
chr17	24473555	24479060	-	ENSMUST00000234543.1	*E4f1*	993,73	13,31	6,56E-04	2,02E-02	Multiple
chr17	24693190	24695706	-	ENSMUST00000234859.1	*Rab26*	4406,18	0,69	3,96E-04	1,41E-02	Spermatogonia
chr17	24718116	24721720	+	ENSMUST00000152407.7	*Rps2*	1793,21	27,09	4,11E-12	1,13E-09	Elongated Spermatids
chr17	25262393	25274622	-	ENSMUST00000174001.7	*Ube2i*	450,95	11,21	2,40E-04	9,56E-03	Multiple
chr17	26091734	26095508	-	ENSMUST00000025007.5	*Nme4*	498,45	0,90	6,31E-08	8,60E-06	Spermatogonia
chr17	26676396	26699644	+	ENSMUST00000015719.14	*Atp6v0e*	8858,31	0,72	1,66E-03	3,92E-02	Multiple
chr17	27630465	27634475	-	ENSMUST00000178774.2	*Rps10*	9152,67	0,71	2,28E-04	9,14E-03	Spermatogonia
chr17	27840087	27851965	+	ENSMUST00000071006.8	*Snrpc*	5768,11	-7,20	2,08E-07	2,53E-05	Multiple
chr17	27840135	27851968	+	ENSMUST00000233752.1	*Snrpc*	1529,18	0,74	2,32E-06	2,11E-04	Multiple
chr17	29492196	29495442	+	ENSMUST00000234478.1	*BC004004*	480,19	9,64	2,12E-03	4,61E-02	Multiple
chr17	29549785	29606895	+	ENSMUST00000048677.8	*Tbc1d22b*	7304,46	-0,89	4,76E-04	1,61E-02	Multiple
chr17	33646235	33685392	-	ENSMUST00000114385.8	*Hnrnpm*	800,94	4,43	1,99E-03	4,41E-02	Multiple
chr17	33824572	33838317	+	ENSMUST00000048249.7	*Ndufa7*	3369,45	-12,84	2,07E-09	3,75E-07	Multiple
chr17	33996053	34000333	-	ENSMUST00000025181.16	*H2-K1*	908,94	9,45	5,34E-04	1,75E-02	Multiple
chr17	43615801	43630266	-	ENSMUST00000233077.1	*Tdrd6*	7638,26	-0,87	8,95E-06	6,70E-04	Multiple
chr17	46646250	46650131	+	ENSMUST00000113429.7	*Mrpl2*	5418,38	0,61	2,12E-03	4,60E-02	Spermatogonia
chr17	46800652	46832314	-	ENSMUST00000233537.1	*Bicral*	2497,07	-2,88	1,12E-09	2,09E-07	NA
chr17	47488050	47502292	-	ENSMUST00000067103.3	*Taf8*	1182,91	0,62	1,69E-04	7,32E-03	Spermatogonia
chr17	47611582	47624418	+	ENSMUST00000024778.2	*Med20*	3841,27	0,74	9,95E-07	9,98E-05	Multiple
chr17	56119678	56122001	-	ENSMUST00000041357.8	*Lrg1*	2147,86	1,21	2,31E-05	1,50E-03	Spermatocytes
chr17	56713936	56717680	-	ENSMUST00000164907.2	*Vmac*	1077,40	0,66	9,82E-04	2,72E-02	Spermatogonia
chr17	57234635	57247672	-	ENSMUST00000233568.1	*Gpr108*	1418,86	0,83	5,15E-08	7,16E-06	Multiple
chr17	65255999	65517229	-	ENSMUST00000233328.1	*Tmem232*	6394,92	-0,65	3,48E-06	2,96E-04	Multiple
chr17	66336988	66385910	-	ENSMUST00000145347.7	*Mtcl1*	1869,27	8,33	1,35E-05	9,47E-04	Multiple
chr17	74395621	74424009	+	ENSMUST00000179074.8	*Slc30a6*	725,31	-6,80	2,67E-04	1,04E-02	Spermatogonia
chr17	79051906	79090378	+	ENSMUST00000040789.5	*Qpct*	6252,86	0,66	3,40E-05	2,04E-03	Pre-leptotene
chr18	10617775	10628230	+	ENSMUST00000002551.4	*Snrpd1*	3327,35	0,63	1,32E-04	6,03E-03	Spermatogonia
chr18	31634383	31723061	+	ENSMUST00000025109.7	*Sap130*	9603,57	-0,65	2,12E-03	4,62E-02	Multiple
chr18	34598615	34624601	-	ENSMUST00000115766.7	*Brd8*	1614,37	1,63	3,49E-08	5,03E-06	Pre-leptotene
chr18	37720369	37841870	+	ENSMUST00000195363.1	*Pcdhgb4*	1535,00	5,72	1,63E-04	7,12E-03	NA
chr18	37765580	37841873	+	ENSMUST00000044851.7	*Pcdhga12*	4020,76	-0,65	2,93E-05	1,81E-03	Multiple
chr18	53681724	53744547	-	ENSMUST00000049811.6	*Cep120*	36521,67	-0,65	8,19E-04	2,39E-02	Multiple
chr18	56524722	56572951	-	ENSMUST00000174518.7	*Aldh7a1*	9703,65	1,65	3,94E-08	5,61E-06	Spermatogonia
chr18	65800549	65817665	+	ENSMUST00000025394.12	*Sec11c*	859,02	0,79	3,13E-09	5,50E-07	Multiple
chr18	74283090	74359986	+	ENSMUST00000114895.3	*Cfap53*	6623,81	-0,65	1,16E-03	3,04E-02	Multiple
chr18	77773956	77782866	+	ENSMUST00000114748.1	*Atp5a1*	61321,95	0,88	3,01E-04	1,14E-02	Multiple
chr18	84011627	84086404	-	ENSMUST00000060303.9	*Tshz1*	1793,05	-3,94	1,57E-15	8,08E-13	Pre-leptotene
chr18	84851338	84880401	+	ENSMUST00000160180.7	*Cyb5a*	849,36	1,54	2,51E-05	1,60E-03	Multiple
chr19	3908870	3912717	-	ENSMUST00000075092.6	*Ndufs8*	869,29	0,98	1,70E-05	1,16E-03	Spermatogonia
chr19	4192158	4195419	+	ENSMUST00000046094.4	*Ppp1ca*	17231,60	0,71	1,25E-03	3,21E-02	Spermatogonia
chr19	4231899	4233631	+	ENSMUST00000025773.3	*Pold4*	628,25	1,01	9,67E-06	7,15E-04	Elongated Spermatids
chr19	5364641	5366645	-	ENSMUST00000025762.14	*Banf1*	9573,56	0,62	4,83E-05	2,70E-03	Multiple
chr19	5601873	5603439	+	ENSMUST00000025864.10	*Rnaseh2c*	583,13	1,44	1,52E-08	2,34E-06	Multiple
chr19	6080033	6080788	+	ENSMUST00000143303.1	*Tmem262*	1390,55	3,00	1,56E-03	3,75E-02	NA

Supplementary Table 3. All differentially expressed genes (*continued*)

Start	End	Strand	Transcript ID	Gene Name	baseMean	log2FoldChange	pvalue	padj	Origin
6363810	6376512	+	ENSMUST00000113488.7	*Sf1*	2287,78	-14,72	3,38E-11	7,88E-09	Multiple
6374129	6375780	+	ENSMUST00000162237.1	*Sf1*	453,21	-7,72	5,66E-50	3,31E-46	Multiple
6909747	6911028	+	ENSMUST00000116551.9	*Trmt112*	2687,22	0,65	9,59E-05	4,70E-03	Pre-leptotene
8967041	8978479	+	ENSMUST00000052248.7	*Eef1g*	95826,96	1,11	2,84E-05	1,77E-03	Multiple
11301249	11314454	-	ENSMUST00000187467.1	*Ms4a14*	61331,71	-1,06	1,58E-03	3,77E-02	Multiple
24875686	24876634	+	ENSMUST00000073080.5	*Gm10053*	3421,45	0,72	4,81E-04	1,62E-02	Spermatogonia
26605119	26778275	+	ENSMUST00000176030.7	*Smarca2*	11615,46	3,95	9,63E-05	4,71E-03	Multiple
26748402	26778275	+	ENSMUST00000209085.1	*Smarca2*	8873,30	-16,68	1,19E-44	4,43E-41	Multiple
46328184	46330446	+	ENSMUST00000026256.8	*Fbxl15*	1803,33	3,00	4,51E-08	6,31E-06	Elongated Spermatids
47737561	47837382	-	ENSMUST00000160247.1	*Cfap43*	63762,83	-0,71	1,38E-03	3,45E-02	Multiple
55298301	55316032	-	ENSMUST00000224897.1	*Zdhhc6*	615,06	3,52	6,87E-07	7,27E-05	Multiple
9459	9806	+	ENSMUST00000082411.1	*mt-Nd3*	20921,04	1,60	1,40E-03	3,49E-02	Pre-leptotene
16522872	16523688	+	ENSMUST00000024026.2	*Cypt1*	13375,65	0,64	7,27E-08	9,75E-06	Elongated Spermatids
20870166	20874733	+	ENSMUST00000115342.9	*Timp1*	386,54	1,53	1,33E-03	3,35E-02	Elongated Spermatids
38121219	38129967	+	ENSMUST00000152730.3	*Rhox13*	1349,50	0,84	7,10E-04	2,13E-02	Spermatogonia
39346267	39346963	+	ENSMUST00000115114.2	*Cypt15*	1611,36	0,66	5,80E-04	1,85E-02	Elongated Spermatids
39862919	39863604	-	ENSMUST00000179096.1	*Cypt14*	385,77	0,75	4,64E-06	3,80E-04	Elongated Spermatids
48695004	48710723	+	ENSMUST00000033433.2	*Rbmx2*	1126,60	0,76	1,79E-03	4,12E-02	Spermatogonia
53434918	53443576	+	ENSMUST00000074232.6	*Etd*	2100,65	0,72	4,40E-04	1,52E-02	Pre-leptotene
53724826	53738441	+	ENSMUST00000069209.1	*4930502E18Rik*	1937,71	0,63	1,50E-05	1,05E-03	Spermatogonia
56374586	56377799	+	ENSMUST00000068106.4	*Smim10l2a*	3448,19	0,75	1,91E-03	4,32E-02	Multiple
58911461	58920304	+	ENSMUST00000062542.4	*4930550L24Rik*	9914,06	0,69	1,69E-04	7,32E-03	Multiple
60891366	60893430	-	ENSMUST00000135107.3	*Sox3*	1532,37	0,68	3,67E-04	1,33E-02	Spermatogonia
66303361	66304224	-	ENSMUST00000033525.2	*4930447F04Rik*	596,88	0,98	4,34E-04	1,51E-02	Round Spermatids
70385877	70389417	+	ENSMUST00000053981.5	*1110012L19Rik*	915,07	0,99	3,32E-04	1,23E-02	Spermatogonia
71555918	71560676	+	ENSMUST00000072699.12	*Hmgb3*	1608,28	0,87	1,12E-04	5,28E-03	Spermatogonia
71816758	71824706	+	ENSMUST00000114576.8	*Vma21*	1919,74	-1,04	2,38E-04	9,48E-03	Spermatogonia
72913532	72918411	-	ENSMUST00000114551.9	*Cetn2*	1275,11	1,18	1,19E-03	3,09E-02	Spermatogonia
73639419	73643514	+	ENSMUST00000019701.8	*Dusp9*	1352,96	0,66	6,96E-07	7,33E-05	Spermatogonia
74223463	74246534	-	ENSMUST00000101454.8	*Flna*	3705,44	-4,09	4,20E-12	1,15E-09	Spermatogonia
96247203	96293438	-	ENSMUST00000050707.2	*Vsig4*	798,96	1,53	4,83E-05	2,70E-03	NA
102247381	102252181	-	ENSMUST00000050551.9	*Cited1*	2901,26	0,62	1,99E-03	4,42E-02	Spermatogonia
102247404	102251852	-	ENSMUST00000101336.9	*Cited1*	6617,36	0,61	7,13E-05	3,71E-03	Spermatogonia
102247714	102250946	-	ENSMUST00000134887.7	*Cited1*	424,16	0,86	1,73E-06	1,63E-04	Spermatogonia
102686292	102686972	-	ENSMUST00000122154.2	*Gm3880*	2082,38	0,77	9,97E-05	4,85E-03	Multiple
102706890	102707635	-	ENSMUST00000113610.2	*Gm9112*	3835,36	0,71	2,13E-04	8,72E-03	Elongated Spermatids
102908905	102909651	+	ENSMUST00000113602.1	*1700011M02Rik*	4328,64	0,64	8,63E-04	2,48E-02	Elongated Spermatids
102928372	102929094	+	ENSMUST00000211116.1	*1700018G05Rik*	3259,16	0,71	7,72E-04	2,28E-02	NA
105499772	105500639	+	ENSMUST00000041758.4	*Cypt2*	16666,02	0,60	7,73E-05	3,95E-03	Elongated Spermatids
134585654	134588056	+	ENSMUST00000113211.7	*Rpl36a*	2547,28	0,84	9,26E-04	2,60E-02	Spermatogonia
136213972	136215513	-	ENSMUST00000058125.8	*Bex1*	559,68	0,86	1,47E-03	3,61E-02	Spermatogonia
139333683	139338169	+	ENSMUST00000054534.3	*Trap1a*	829,14	0,59	2,49E-06	2,24E-04	Spermatogonia
142317993	142390535	-	ENSMUST00000033634.4	*Acsl4*	1954,94	-2,95	1,53E-03	3,70E-02	Spermatogonia
150806717	150812715	-	ENSMUST00000112697.9	*Maged2*	9528,00	0,60	1,08E-07	1,41E-05	Spermatogonia
3774805	3782910	+	ENSMUST00000180202.1	*Gm3376*	1400,04	0,61	3,10E-04	1,16E-02	Spermatogonia

Supplementary Table 4. Selected Gene Ontology term identified by DAVID (down-regulated genes)

Term	Count	PValue	Genes	Fold Enrichment
GO:0000775~chromosome, centr. region	5	4,58E-02	PHF2, PPP2R5C, CENPF, TNKS, CENPE	3,708691716
GO:0005925~focal adhesion	9	3,35E-02	GNA13, SYNE2, LIMK1, RPL22, SSH2, SNAP23, FLNA, PXN, MPRIP	2,407329814
GO:0006351~transcription, DNA-templ.	28	1,87E-02	ZFP541, TSHZ1, SETD1B, TAF1D, EZH1, NAA15, BBX, TTLL5, MYEF2, CNOT1, CNOT4, EDRF1, RB1CC1, SERTAD2, RFX4, SF1, NR4A1, ZFP148, SPEN, MED10, ZFP37, SENP2, PHF2, MTF1, SAP130, IRF6, JMJD1C, SMARCA2	1,561581642
GO:0005911~cell-cell junction	7	1,26E-02	PCDHGA12, OCLN, AHI1, SNAP23, CEACAM2, ADGRL3, FLNA	3,642267386
GO:0015031~protein transport	13	1,07E-02	SDAD1, ABCB9, EXOC7, RRBP1, LMAN2L, CENPF, GCC2, SENP2, TRAM2, IPO5, TNKS, SNAP23, SNX13	2,308552011
GO:0003723~RNA binding	16	7,21E-03	SETD1B, PTBP1, SF1, TRMT1, SPEN, CNOT4, NSUN7, ANKRD17, RPL22, UBR5, SNRPC, NSUN2, RBM25, RBMS1, PRPF40A, NSUN4	2,155823293
GO:0008270~zinc ion binding	20	6,34E-03	ZCCHC2, CIZ1, LIMK1, SF1, NRD1, NR4A1, NPEPPS, MARCH10, PXN, ZFP37, CNOT4, TRIM37, PHF2, TRIM42, ZMIZ2, UBR5, P2RX2, TNKS, SNRPC, BAZ2B	1,955281591
GO:0032259~methylation	7	5,46E-03	ETFBKMT, NSUN7, SETD1B, EZH1, TRMT1, NSUN2, NSUN4	4,354410348
GO:0005524~ATP binding	30	1,76E-04	DNAH10, ABCA9, CDK18, TTLL8, DNAH17, TTLL4, TTLL5, HK1, MYO9A, ACSF3, LATS2, DNAH6, ACSL1, DGKE, AAK1, DYNC2H1, ABCA17, ABCA15, DYNC1H1, ACSL4, ABCB9, PAN3, ABCB8, LIMK1, PIK3CD, CENPE, P2RX2, GRK3, TSSK3, SMARCA2	2,092164278
GO:0005856~cytoskeleton	28	7,04E-06	STIL, CEP120, TTLL8, CEP126, DNAH17, SSH2, TTLL4, TTLL5, AHI1, CENPE, CNTRL, PCM1, FLNA, PXN, LATS2, MPRIP, CFAP54, SASS6, SYNE2, FNBP1L, CAMSAP2, DYNC2H1, MAP4, TNKS, ODF2, DYNC1H1, NSUN2, DNM1	2,631071859
GO:0042384~cilium assembly	10	3,80E-06	CFAP54, CFAP53, TTLL8, RFX4, FNBP1L, CEP126, DYNC2H1, AHI1, PCM1, FLNA	8,149450153
GO:0016887~ATPase activity	13	4,52E-07	DNAH10, DNAH7C, ABCB9, ABCB8, ABCA9, DNAH17, CENPE, DNAH6, DYNC2H1, ABCA17, ABCA15, DYNC1H1, SMARCA2	6,83126506

Supplementary Table 5. Selected Gene Ontology term identified by DAVID (up-regulated genes)

Term	Count	PValue	Genes	Fold Enrichment
GO:0006260~DNA replication	6	4,36E-02	POLD4, CDC45, BLM, NOL8, E4F1, RPA3	3,11678014
GO:0051301~cell division	12	3,35E-02	CCNE2, PPP1CA, CDC45, GNAI2, FBXO5, CETN2, UBE2I, E4F1, PMF1, CCNA2, SEPT9, TERF1	2,05007464
GO:0005764~lysosome	11	3,20E-02	PLEKHF1, WDR48, AGA, CTSL, LAMTOR1, CCDC115, HEXB, BLOC1S1, CST3, CTSD, CTSA	2,16364218
GO:0098641~cadherin binding in cell-cell adh.	10	3,14E-02	RPL14, NCK1, RPL15, EEF1G, GIPC1, UBAP2, RPS2, PARK7, SEPT9, ANXA2	2,29049326
GO:0071013~catalytic step 2 spliceosome	6	1,61E-02	HNRNPM, EIF4A3, MAGOH, SNRPD1, RBMX2, LSM3	4,06912252
GO:0015992~proton transport	5	1,27E-02	ATP6V0E, ATP6V1H, ATP5A1, ATP6V0B, ATP5K	5,50810284
GO:0016853~isomerase activity	7	9,30E-03	FKBP8, HSD3B6, PTPA, MCEE, FKBP3, PDIA5, PIN1	3,88985507
GO:0005783~endoplasmic reticulum	33	6,93E-03	PLPP7, HSD3B6, UCHL1, EIF5A, PDIA5, G6PC3, HMOX2, ALB, BOK, H2-K1, AGA, REEP6, GABARAPL1, CREB3, SEC11C, ZDHHC6, CST3, S100A10, MGP, CYB5A, PIGP, PARK7, TMEM106C, SEC61B, BAX, NCK1, RHEB, EEF1G, DPM3, SPCS2, STBD1, RDH14, MGST1	1,62395819
GO:0006412~translation	20	1,88E-05	RPL18, EIF6, MRPL2, MRPL52, MRPS16, RPL36A, RPL14, RPL15, EIF5A, GM10260, RPS27L, MRPS7, RPS2, MRPL12, RPS19, RPL13A, RPL31, RPL9, EEF1G, RPS21	3,18673281
GO:0002181~cytoplasmic translation	7	1,27E-05	GM10073, RPL31, RPL9, RPLP0, RPLP1, RPL15, RPLP2	13,1546456
GO:0005840~ribosome	22	1,34E-12	RPL18, MRPL2, MRPL52, MRPS16, RPL36A, RPL14, RPL15, RPLP2, RPS27L, MRPS7, RPS2, PRMT3, MRPL12, RPS19, RPL13A, RPL31, RPL9, NCK1, RPLP0, RPLP1, RPS10, RPS21	7,61878258

Supplementary Table 6. KEGG pathway analysis

The functional annotation analysis revealed significant enrichment in KEGG Pathway – Parkinson’s disease (p-value = 4.2E-3)

**Parkinson's disease**	
Atp5a1	ATP synthase, H+ transporting, mitochondrial F1 complex, alpha subunit 1(Atp5a1)
Ndufa7	NADH dehydrogenase (ubiquinone) 1 alpha subcomplex, 7 (B14.5a)(Ndufa7)
Ndufb2	NADH dehydrogenase (ubiquinone) 1 beta subcomplex, 2(Ndufb2)
Ndufs8	NADH dehydrogenase (ubiquinone) Fe-S protein 8(Ndufs8)
Park7	Parkinson disease (autosomal recessive, early onset) 7(Park7)
Cox7a2	cytochrome c oxidase subunit VIIa 2(Cox7a2)
Cox7c	cytochrome c oxidase subunit VIIc(Cox7c)
Cox6b1	cytochrome c oxidase, subunit VIb polypeptide 1(Cox6b1)
Cycs	cytochrome c, somatic(Cycs)
Gnai2	guanine nucleotide binding protein (G protein), alpha inhibiting 2(Gnai2)
Uchl1	ubiquitin carboxy-terminal hydrolase L1(Uchl1)
